# Supplementary material for: Crystallographic fragment screening supports tool compound discovery and reveals conformational flexibility in human deoxyhypusine synthase
Source: Commun Chem. 2026 Jan 17;9:66. doi: 10.1038/s42004-026-01897-9 (PMC12868627; doi:10.1038/s42004-026-01897-9)
Supplement: Supplementary file 2 — Supplementary Files [file 42004_2026_1897_MOESM2_ESM.pdf]

Supplementary Files

Crystallographic fragment screening supports tool compound discovery and reveals conformational flexibility in human deoxyhypusine synthase.

Piotr Wilk[1], Elżbieta Wątor-Wilk[2], Damian Muszak[3], Paweł Kochanowski[1,4], Tobias Krojer[5], Przemysław Grudnik[1*]

[1] Małopolska Centre of Biotechnology, Jagiellonian University, Gronostajowa 7A, Kraków, Poland

[2] Jerzy Haber Institute of Catalysis and Surface Chemistry Polish Academy of Sciences, Niezapominajek 8, Kraków, Poland

[3] Faculty of Chemistry, Department of Organic Chemistry, Jagiellonian University, Gronostajowa 2, Krakow, Poland

[4] Doctoral School of Exact and Natural Sciences, Jagiellonian University, Krakow, Poland

[5] MAX IV Laboratory, Lund University, Fotongatan 2, 224 84 Lund, Sweden

Correspondence: przemyslaw.grudnik@uj.edu.pl

Supplementary Figures:

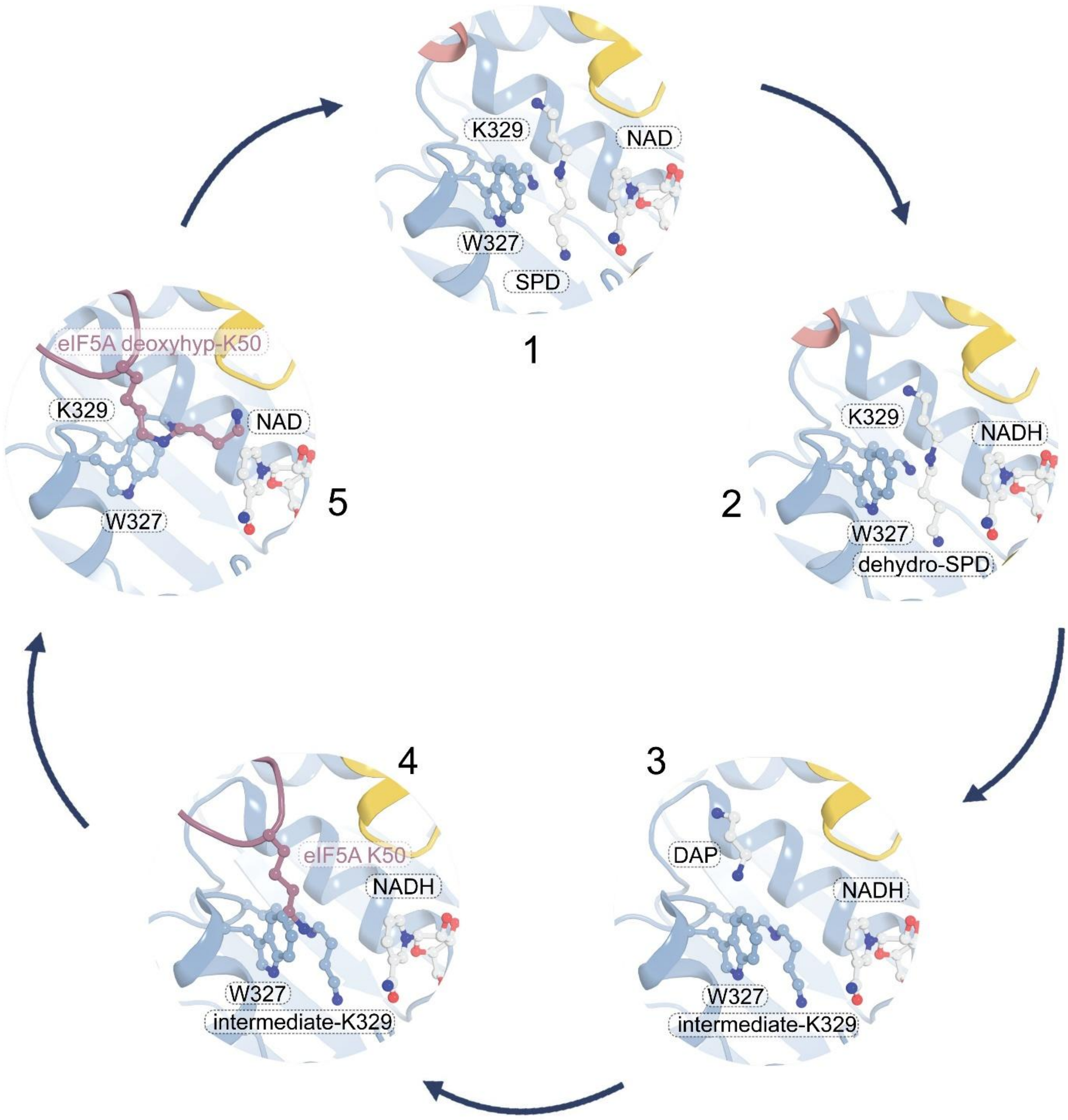

Fig. S1. Mechanism of reaction catalyzed by DHS. (1) Spermidine (SPD) is bound in the enzymes' active site between NAD cofactor and catalytic K329 and is shielded from the bulk solvent by W327. (2) Upon hydride transfer with a concomitant reduction of NAD a dehydrogenated spermidine is formed which can subsequently bind to the Nε of the catalytic lysine forming an intermediate product (3) and releasing a diaminopropane (DAP) as a side product. The entrance to the active site is opened allowing for binding of the hypusine loop of a target substrate - eIF5A (4). The 4-aminobutyl moiety is transferred from the transiently modified DHS^{K329} to the final acceptor $eIF5A^{K50}$ and with oxidation of NADH is finally reduced to a stable deoxyhypusine (5).

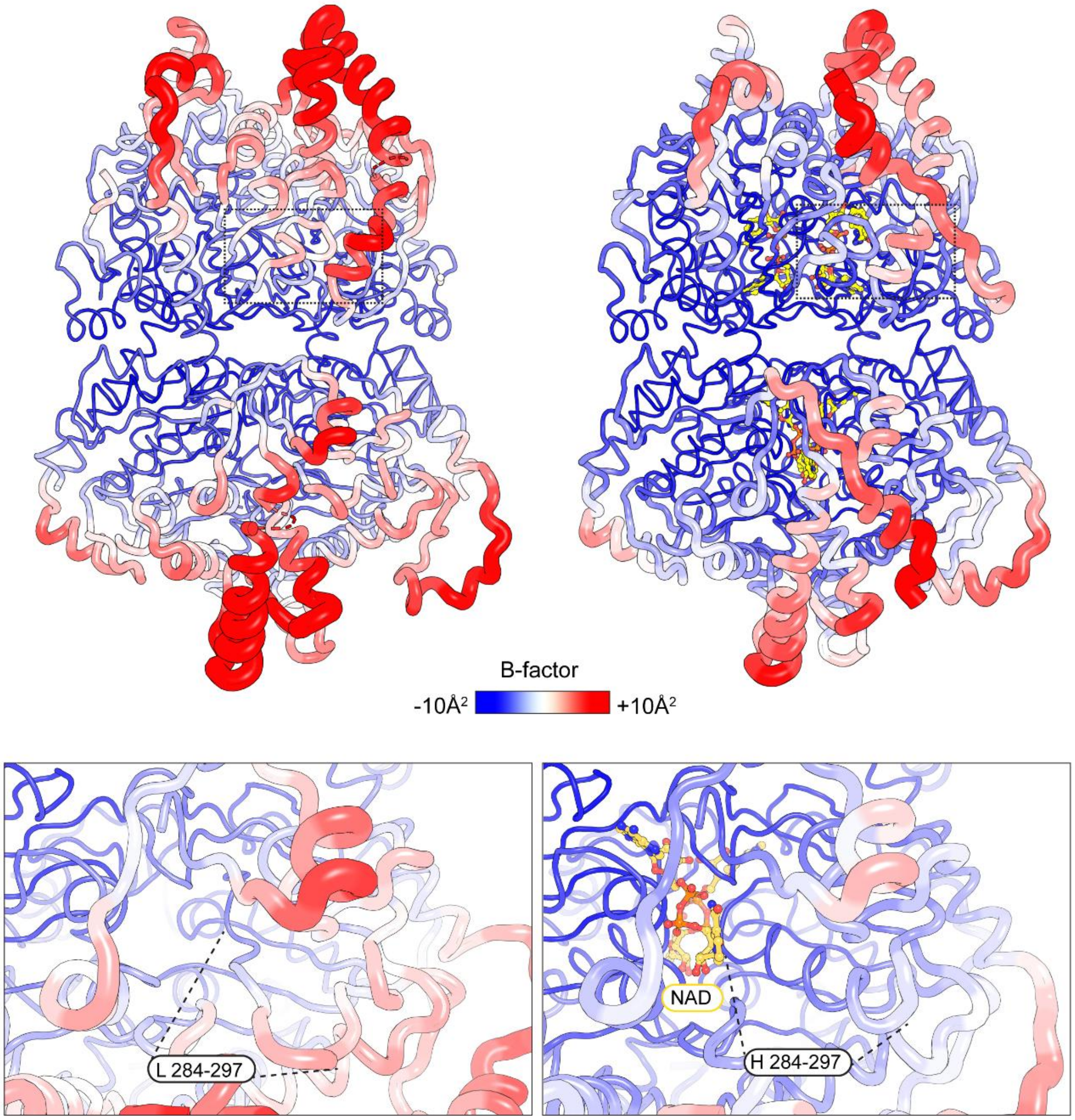

Fig. S2. NAD binding stabilizes the conformation of DHS, particularly in loop 285–305. The overall DHS structure is colored based on deviation of local B-factors from the average, and the thickness of the cartoon tube reflects the local B-factor of the main chain. Note that the ball-and-chain motif, although highly flexible in the NAD-bound structure (right), is absent in the apo form (left). Insets below each structure provide close-up views of the region of interest. NAD is shown as a ball-and-stick model with carbon atoms colored yellow.

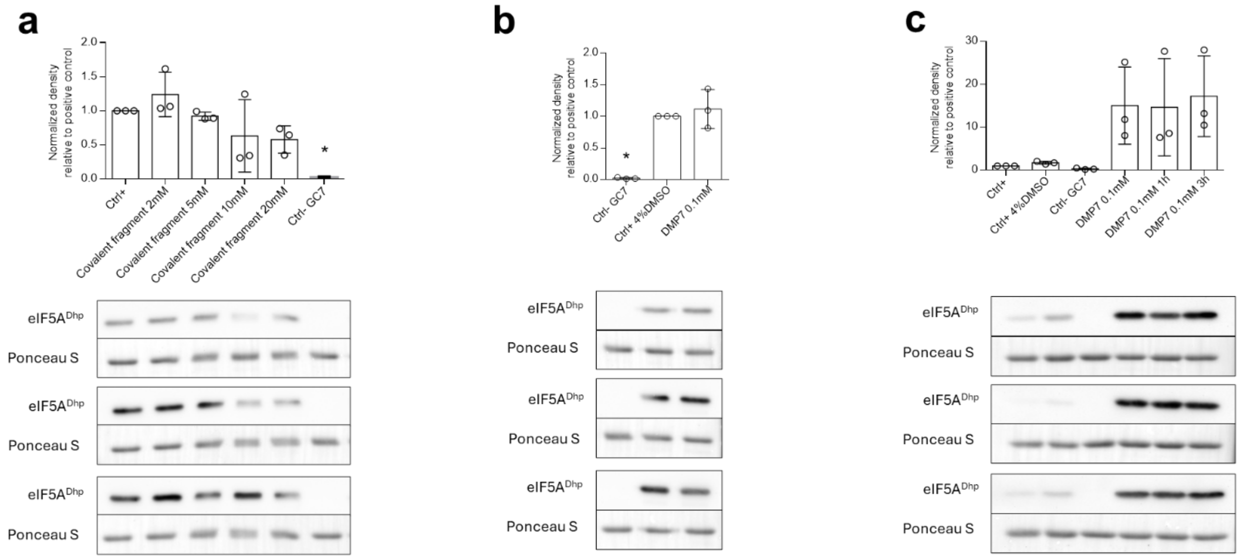

		P-value
ANOVA		0.002
Dunnett's multiple comparisons test	Ctrl+ vs. Covalent fragment 2mM	0.719
	Ctrl+ vs. Covalent fragment 5mM	0.995
	Ctrl+ vs. Covalent fragment 10mM	0.362
	Ctrl+ vs. Covalent fragment 20mM	0.254
	Ctrl+ vs. Ctrl- GC7	0.003

		P-value
ANOVA		0.001
Dunnett's multiple comparisons test	Ctrl+ 4%DMSO vs. Ctrl- GC7	0.001
	Ctrl+ 4%DMSO vs. DMP7 0.1mM	0.669

		P-value
ANOVA		0.024
Dunnett's multiple comparisons test	Ctrl+ vs. Ctrl+ 4%DMSO	1.000
	Ctrl+ vs. Ctrl- GC7	1.000
	Ctrl+ vs. DMP7 0.1mM	0.111
	Ctrl+ vs. DMP7 0.1mM 1h	0.124
	Ctrl+ vs. DMP7 0.1mM 3h	0.058

Fig. S3. Effect of ligands on eIF5a deoxyhypusination with (a) VT00065, (b) DMP7 and (c) DMP7 preincubated with DHS. The top line shows plots depicting normalized intensity ± SD. Middle panel shows biological triplicates for all three assays with rabbit FabHpu98 western blot and Ponceau S staining as a loading control. Bottom panels tabularize statistical significance test for each assay.

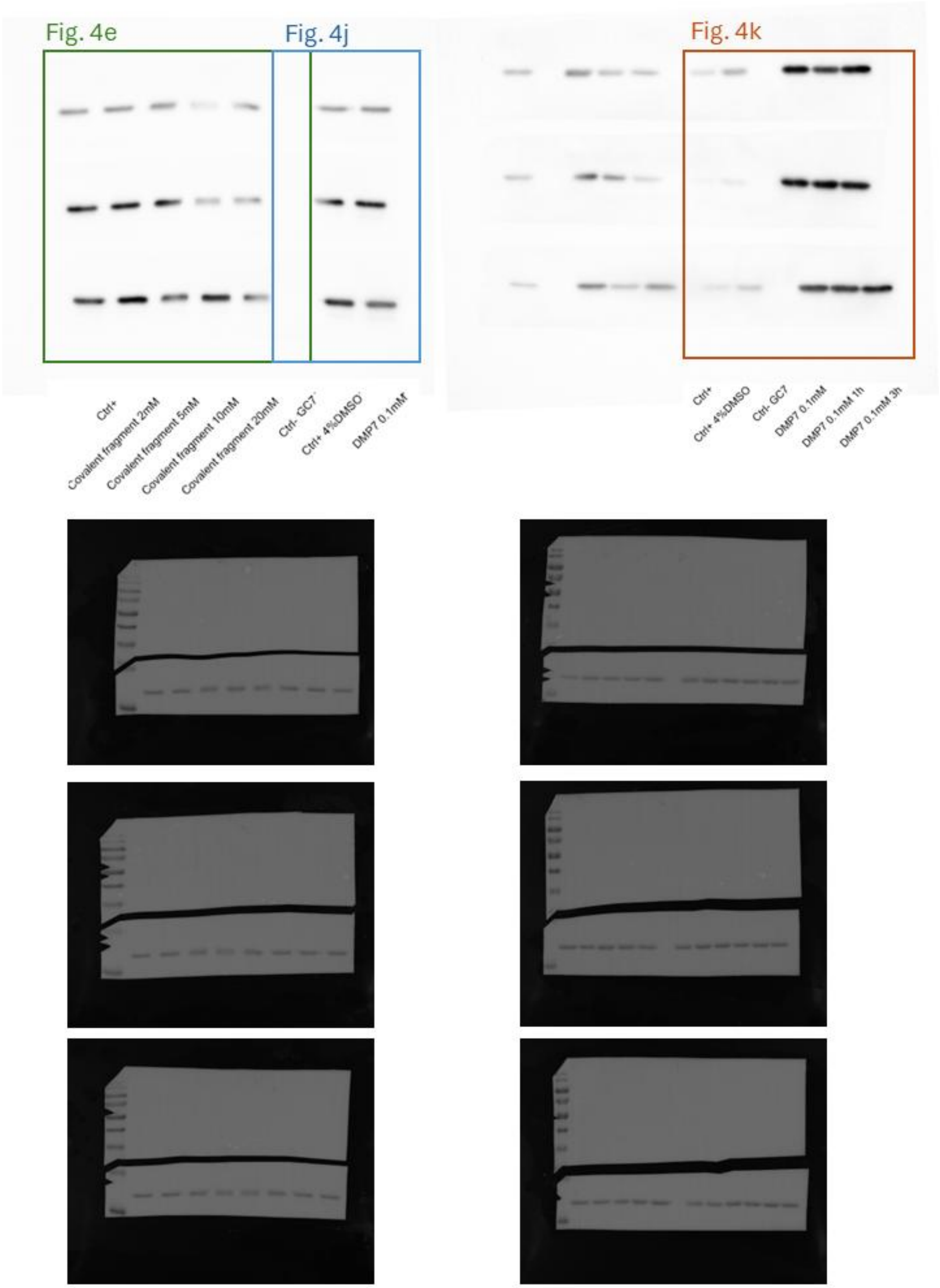

Fig. S4. Full, uncropped blot images used in the main Figure 4.

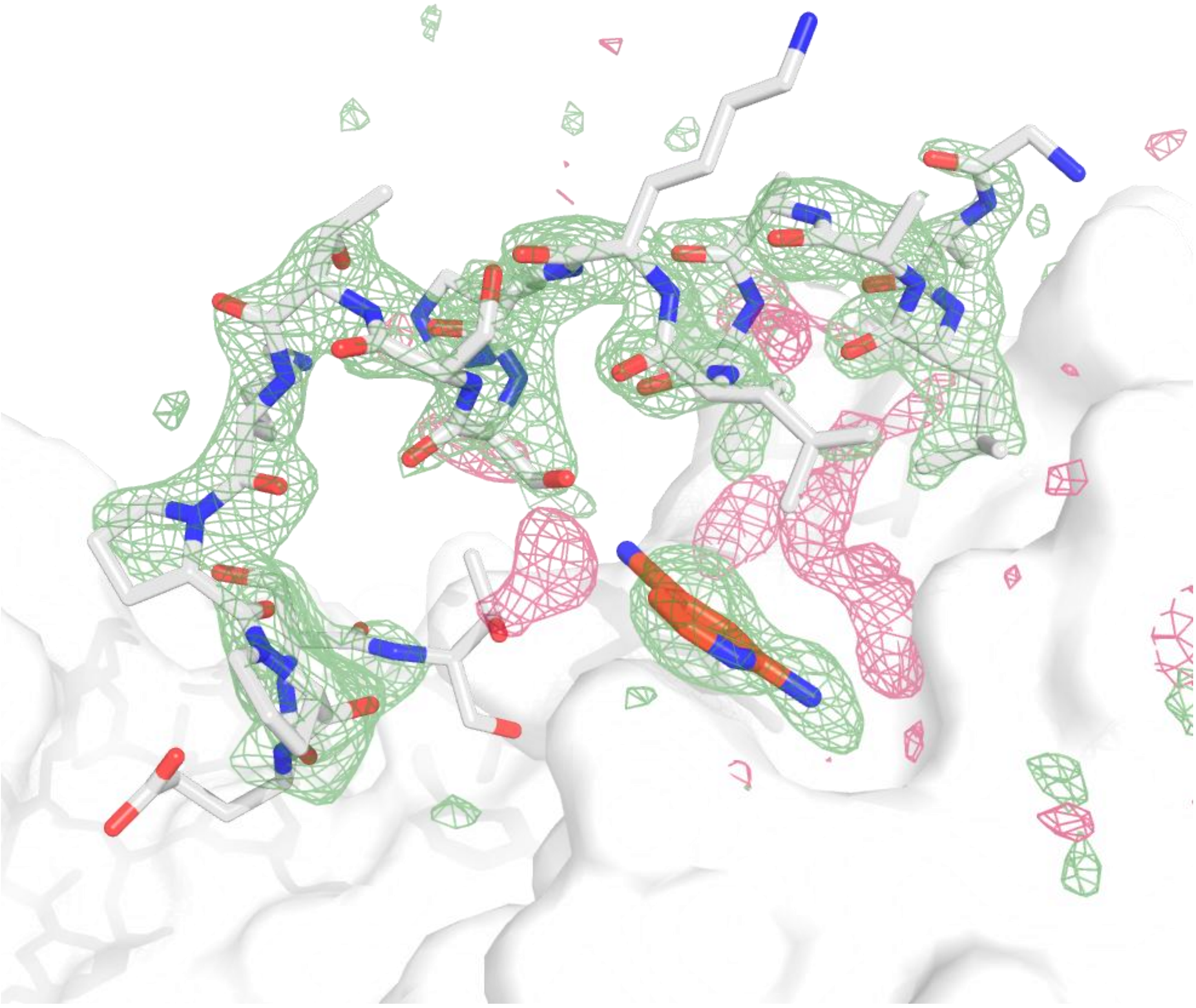

Fig. S5. Stabilisation of ball-and-chain motif by fragments. Here, the clear fo-fc electron density in the vicinity of the identified ligand (VT00143) is shown. The previously unmodelled N-terminus (residues 12-29) could be clearly traced in this map, showing its orientation significantly different from the one observed in unliganded DHS. Other fragments exhibited a similar stabilizing effect, and the full list of hits is listed in Supplementary File S5.

Supplementary methods

1. Crystal preparation and diffraction data analysis

All samples for crystallographic fragment screening were prepared at the FragMAX platform [1,2]. The previously optimized crystallization conditions were used to generate a large quantity of HsDHS crystals. In the 1st campaign, to retain possibly many binding pockets accessible for fragments and hence to maximize the anticipated hit rate we omitted NAD (a cofactor) from the crystallization. Obtained crystals were soaked in either a blank DMSO solution (for the apo, ground state) or with a FragMAXlib fragment library[3]. Following soaking the crystals were individually fished, flash-cooled in LN_2, and diffraction data were recorded at BioMAX beamline. The subsequent data processing and analyses were conducted using the FragMAXapp pipeline[3].

Crystals of DHS-NAD complex grown in previously optimized conditions were soaked O/N with 100mM ligands (final DMSO concentration =10%) arranged in a FragMaxLib fragment library; two crystals were harvested for each ligand. Diffraction data for DHS crystals soaked with fragments were collected at BioMAX [2] beamline at X-ray wavelength 0.9763 Å. All data were analyzed using FragMaxApp[4] with data reduction by XDSAPP[5] followed by phasing/refinement by DIMPLE[6] and hit identification by PanDDA[7]. Hit inspection was aided by pandda.inspect tool adapted for Windows desktop [https://github.com/tkrojer/pandda_inspect_tools]. For the verified hits a respective ligand model prepared from the SMILES string with AceDRG[8] was placed using COOT[9] and oriented guided primarily by pandda Z and event maps supported by the native 2fo-fc and fo-fc maps. In most cases model curation was limited to ligand placement and setting its occupancy to fractional, however if large structural changes were induced a protein chain was rebuilt (mostly concerning N-termini). All DHS-fragment models were subjected to default Refmac5 refinement protocol. The better structure (usually higher resolution) from the duplicate was deposited in the Protein Data Bank[10]. No attempt was made to fine tune the data reduction nor the refinement protocol and we acknowledge that some parameters (e.g. resolution cutoff, in XDS based primarily on CC1/2 value[11] may appear suboptimal, nevertheless we inspected all structures with due diligence to minimize number of significant errors.

Crystal of DHS soaked with DMP7 compound was measured at ALBA BL13 - XALOC beamline[12] at 0.9793 Å. XDSAPP3 was used for the data reduction and phaser for molecular replacement [cit] followed by model rebuilding in COOT[9] and refinement using phenix.refine[13]. Ligand model and restrains were generated from SMILES string with AceDRG[14]. Since the placed ligand crossed 2-fold crystallographic axis and overlapped with itself (except for ketone (=O) from the linker, which is located asymmetrically) the diffraction data was reprocessed in lower symmetry space group (P3(2) vs. P3(2)21). With this an entire homotetramer was placed in ASU with two copies of DMP7 ligand and model refined with phenix.refine. The final data collection and refinement statistics for all deposited structures are summarized in the supplementary table S1.

2. Chemical syntheses.

2.1. Preparation of linked pyroquilone 6 (DMP7)

All syntheses were performed according to procedures summarized in Scheme 1. Reagents were obtained from commercial suppliers (Merck, AmBeed, Angene) and used without further purification, unless otherwise noted. Anhydrous solvents were purchased from Merck. Nuclear magnetic resonance spectra were recorded on a Bruker Avance 600 spectrometer {1H NMR (600 MHz), 13C NMR (151 MHz)}. Chemical shifts (δ) for 1H NMR were reported in parts per million [ppm] referring to the solvent peak employed, and the coupling constants were in Hertz [Hz]. The following abbreviations were used for spin multiplicity: s = singlet, d = doublet, t = triplet, q = quartet, quin = quintet, dd = double of doublets, ddd = double doublet of doublets, m = multiplet. Chemical shifts (δ) for 13C NMR were reported in parts per million [ppm] relative to the solvent peak. IR absorption spectra were recorded on a Nicolet IR200 spectrometer using the ATR technique. Melting points were measured using a Stuart SMP30 apparatus. Thin layer chromatography was performed on Sigma-Aldrich precoated silica gel plates (0.20 mm thick, particle size 25 mm). Spots were visualized by UV light at 254 and 365 nm. Flash column chromatography was performed on a Reveleris® X2 Flash Chromatograph, using Büchi FlashPure EcoFlex Silica cartridges. HRMS analysis was carried out by the Laboratory for Forensic Chemistry Faculty of Chemistry, Jagiellonian University with the microOTOF-QII (Bruker) mass spectrometer using ESI ionization technique.

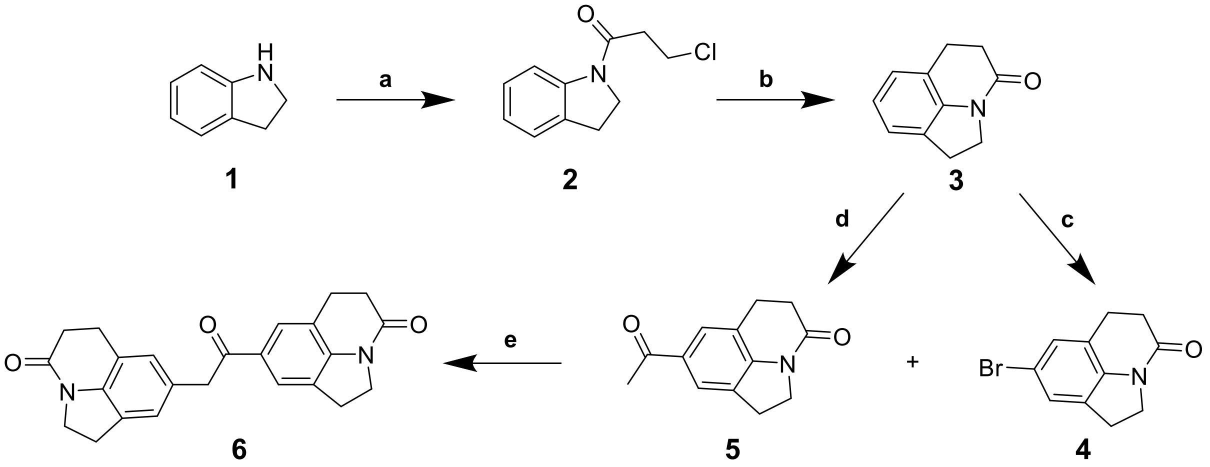

Scheme 1. Synthesis route leading to coupled pyroquilone **6**. Reagents and conditions: (a) Indoline (**1**), 3-chloropropanoyl chloride, anh. Et_3N, anh. DCM, RT, overnight, 75%; (b) **2**, $AlCl_3$, 140°C, 4h, 69%; (c) **3**, NBS, anh. DMF, 0°C, 2h, 72%; (d) **3**, acetyl chloride, $AlCl_3$, anh. DCM, reflux overnight, 74%; (e) 4, 5, BrettPhos Pd G1 MTBE complex, potassium *tert*-butoxide, anh. THF, reflux overnight, 61%.

2.2. Synthesis of N-acylated indoline derivative 2

Indoline (25.00 g, 209.8 mmol, 1.0 eq) and anhydrous triethylamine (58.40 ml, 419.6 mmol, 2.0 eq) were placed in a round bottom flask flushed previously with argon, then dissolved in anhydrous DCM (120 ml) and the obtained mixture was cooled to 0°C using an ice bath. In the next step, 3-chloropropionyl chloride (30.27 ml, 314.7 mmol, 1.5 eq) was added dropwise over 1 hour, then the mixture was allowed to warm up to room temperature and stirred overnight. The next day solution was diluted with DCM (500 ml) and transferred to a separatory funnel. The organic phase was washed with 2M HCl, 2M NaOH and water, then dried over anhydrous $MgSO_4$. The solvent was evaporated and the solid recrystallized in ethanol giving the desired product **2** as a brownish solid with a yield of 75% (32.99 g). Molecule **2** is known in the literature[15].

R_f = 0.47 (SiO_2, Hex/AcOEt, 1/1); **mp** = 87-88ºC; **^{1}H NMR** (600 MHz, $CDCl_3$) δ [ppm] 8.22 (d, *J* = 8.0 Hz, 1H), 7.22 – 7.15 (m), 7.03 (t, *J* = 7.4 Hz, 1H), 4.04 (t, *J* = 8.5 Hz, 2H), 3.89 (t, *J* = 6.9 Hz, 2H), 3.19 (t, *J* = 8.4 Hz, 2H), 2.88 (t, *J* = 6.9 Hz, 2H); **^{13}C NMR** (151 MHz, $CDCl_3$) δ [ppm] 167.8, 142.7, 131.2, 127.7, 124.7, 124.1, 117.1, 48.0, 39.4, 38.8, 28.0; **IR (ATR)** [cm^{-1}]: 3072, 2960, 2918, 1651, 1416, 760.

2.3. Synthesis of pyroquilone 3

Intermediate **2** (7.13 g, 34.00 mmol, 1 eq) and $AlCl_3$ (24.99 g, 187.49 mmol, 5.5 eq) were placed in a round-bottom flask previously flushed with argon and then heated at 140ºC for 4 hours. After this time, the solution was cooled to 0°C and a water/ice mixture (100 ml) was added to neutralize excess $AlCl_3$. The crude obtained was extracted with AcOEt (3x50 ml), organics were dried over anhydrous $MgSO_4$ and the solvent evaporated. The product was purified by flash column chromatography (SiO_2, Hex/AcOEt), giving product **3** as a colorless solid with a yield of 69% (4.06 g). Molecule **3** is known in the literature[16].

R_f = 0.12 (SiO_2, Hex/AcOEt, 1/1); **mp** = 104-105ºC **^{1}H NMR** (600 MHz, $CDCl_3$) δ [ppm] 6.94 (d, J = 7.4 Hz, 1H), 6.85 (dd, J = 7.5, 0.5 Hz, 1H), 6.79 (t, J = 7.4 Hz, 1H), 3.94 – 3.87 (m, 2H), 3.08 – 2.98 (m, 2H), 2.81 (t, J = 7.8 Hz, 2H), 2.52 (t, J = 7.8 Hz, 2H); **^{13}C NMR** (151 MHz, $CDCl_3$) δ [ppm] 167.1, 140.8, 128.5, 124.9, 122.8, 122.8, 119.7, 44.7, 31.2, 27.3, 24.0; **IR (ATR)** [cm^{-1}]: 3046, 2913, 2900, 1644, 1390.

2.4. Bromination of pyroquilone leading to molecule 4

Intermediate **3** (1.16 g, 6.70 mmol, 1 eq) was dissolved in anhydrous DMF (10 ml), the mixture was cooled to 0°C and a solution of NBS (1.25 g, 7.03 mmol, 1.05 eq) in anhydrous DMF (10 ml) was added dropwise there. The mixture was then stirred for 2 hours at 0°C, after which transferred to a separatory funnel containing 30 ml of water, extracted with ethyl acetate (3x20 ml) and organics dried over anhydrous $MgSO_4$. The solvent was evaporated and the product purified by flash column chromatography (SiO_2, Hex/AcOEt), giving **4** as a yellowish solid with 72% (1.22 g) yield. Molecule **4** is known in the literature[17].

R_f = 0.30 (SiO_2, AcOEt); **mp** = 106-108ºC **^{1}H NMR** (600 MHz, $CDCl_3$) δ [ppm] 7.16 – 7.14 (m, 1H), 7.09 – 7.07 (m, 1H), 4.06 – 4.01 (m, 2H), 3.13 (t, J = 8.5 Hz, 2H), 2.91 (t, J = 7.8 Hz, 2H), 2.62 (t, J = 7.8 Hz, 2H);**^{13}C NMR** (151 MHz, $CDCl_3$) δ [ppm] 167.3, 140.5, 130.9, 128.3, 126.4, 121.8, 115.4, 45.3, 31.3, 27.6, 24.2; **IR (ATR)** [cm^{-1}]: 3036, 2954, 2898, 1656, 1379, 859.

2.5. Acetylation of pyroquilone leading to molecule 5

Intermediate **3** (1.09 g, 5.77 mmol, 1 eq) and $AlCl_3$ (5.39 g, 40.41 mmol, 7 eq) were placed in an argonated round-bottom flask and dissolved in anhydrous DCM (30 ml). Next, acetyl chloride (0.62 ml, 8.66 mmol, 1.5 eq) was added dropwise at room temperature and the mixture refluxed overnight. The next day, the solution was cooled to ambient temperature, reaction quenched with a water/ice mixture (50 ml) and extracted with AcOEt (3x30 ml). The organics were dried over anhydrous $MgSO_4$, solvent was removed and the product purified by flash column chromatography (SiO_2, Hex/EtOAc), giving **5** as a pinkish solid with 74% (0.92 g) yield. Molecule **5** is known in the literature[18].

R_f = 0.27 (SiO_2, AcOEt); **mp** = 94-96ºC **^{1}H NMR** (600 MHz, $CDCl_3$) δ [ppm] 7.54 (s, 1H), 7.50 (s, 1H), 3.96 – 3.90 (m, 2H), 3.05 (t, J = 8.5 Hz, 2H), 2.85 (t, J = 7.8 Hz, 2H), 2.52 (t, J = 7.8 Hz, 2H), 2.37 (s, 3H);**^{13}C NMR** (151 MHz, $CDCl_3$) δ [ppm] 196.4, 167.5, 145.3, 132.7, 128.9, 126.6, 123.8, 119.3, 45.4, 31.0, 26.9, 26.2, 23.8; **IR (ATR)** [cm^{-1}]: 3554, 3387, 3064, 2964, 1640, 1585, 1383, 1188.

2.6. Palladium-catalyzed coupling leading to final molecule 6

Intermediates **4** (0.386 g, 1.53 mmol, 1.10 eq) and **5** (0,300 g, 1.39 mmol, 1.00 eq) were placed in a round-bottom flask, dissolved in anhydrous THF (10 ml) and resulting mixture was bubbled with argon for 10 minutes. In the next step, potassium *tert*-butoxide (0.702 g, 6.26 mmol, 4.50 eq) and BrettPhos Pd G1 MTBE complex (0.062 g, 0.07 mmol, 0.05 eq) were added and the solution was refluxed overnight. The reaction was quenched by adding water (10 ml), mixture transferred into a separatory funnel and extracted with chloroform (3x15 ml). Organics were dried over anhydrous $MgSO_4$ and the product was purified by flash column chromatography (SiO_2, AcOEt/MeOH) giving **6** as beige solid with 61% (0.328 g) yield.

R_f = 0.17 (SiO_2, EtOAc/MeOH, 9/1); **mp** = slow decomposition starts at 198ºC; **^{1}H NMR** (600 MHz, $CDCl_3$) δ [ppm] 7.76 (s, 1H), 7.72 (s, 1H), 6.95 (s, 1H), 6.87 (s, 1H), 4.15 (s, 2H), 4.13 – 4.09 (m, 2H), 4.07 – 4.02 (m, 2H), 3.21 (t, J = 8.5 Hz, 2H), 3.15 (t, J = 8.4 Hz, 2H), 3.01 (t, J = 7.8 Hz, 2H), 2.92 (t, J = 7.8 Hz, 2H), 2.70 (t, J = 7.8 Hz, 2H), 2.64 (t, J = 7.8 Hz, 2H);**^{13}C NMR** (151 MHz, $CDCl_3$) δ [ppm] 196.7, 167.9, 167.6, 145.9, 140.5, 132.5, 129.9, 129.4, 129.4, 127.3, 126.6, 124.5, 124.5, 120.3, 119.9, 45.8, 45.4, 45.0, 31.6, 31.5, 27.8, 27.4, 24.5, 24.3; **HRMS (ESI+):** calcd for $C_{24}H_{22}N_2O_3$ [m/z] $[M+Na]^+$ 409.1523; found $[M+Na]^+$ 409.1521; **IR (ATR)** [cm^{-1}]: 2913, 2851, 1656, 1591, 1380.

3. Copies of ^{1}H and ^{13}C NMR spectra of final molecule 6 (DMP7).

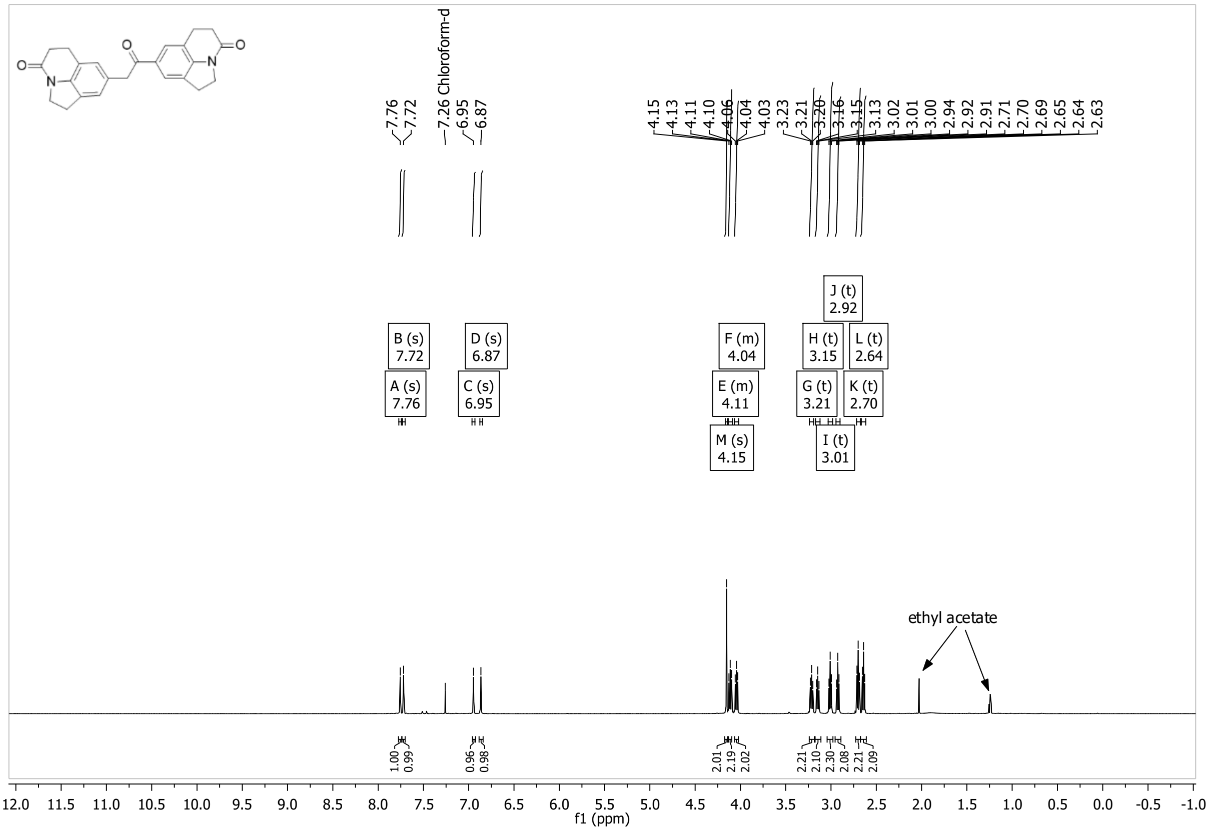

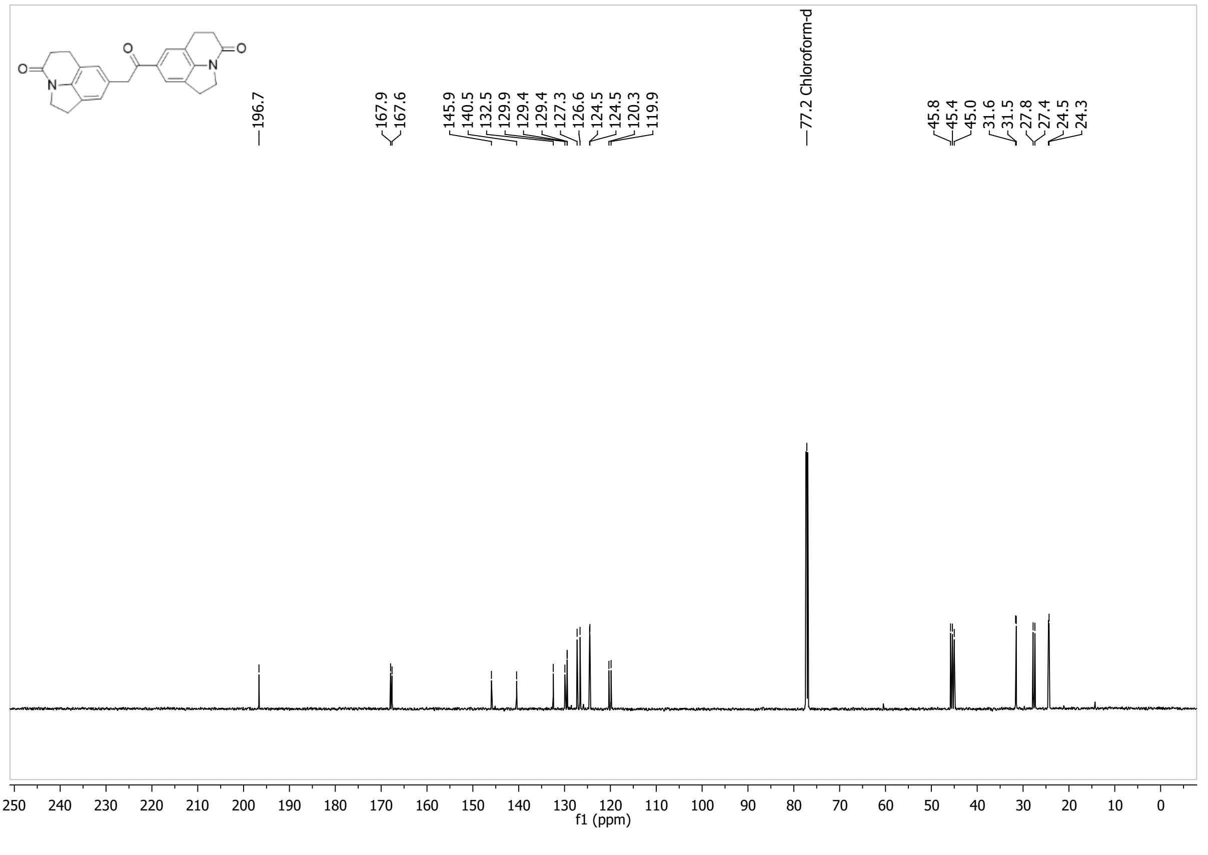

References

1. Lima, G. M. A. *et al.* FragMAX: the fragment-screening platform at the MAX IV Laboratory. *Acta Crystallogr D Struct Biol* **76**, 771–777 (2020).

2. Kanchugal P., S. *et al.* FragMAX Facility for Crystallographic Fragment and Ligand Screening at MAX IV. *Applied Research* **4**, e202400263 (2025).

3. Lima, G. M. A. *et al.* FragMAX: the fragment-screening platform at the MAX IV Laboratory. *Acta Crystallogr D Struct Biol* **76**, 771–777 (2020).

4. Lima, G. M. A. *et al.* *FragMAXapp* : crystallographic fragment-screening data-analysis and project-management system. *Acta Crystallogr D Struct Biol* **77**, 799–808 (2021).

5. Sparta, K. M., Krug, M., Heinemann, U., Mueller, U. & Weiss, M. S. *XDSAPP2.0*. *Journal of Applied Crystallography* **49**, 1085–1092 (2016).

6. Winter, G. *et al.* *DIALS* : implementation and evaluation of a new integration package. *Acta Crystallogr D Struct Biol* **74**, 85–97 (2018).

7. Pearce, N. PanDDA: extracting ligand-bound protein states from conventionally uninterpretable crystallographic electron density. *Acta Crystallographica Section A Foundations and Advances* **75**, e43–e43 (2019).

8. Long, F. *et al.* *AceDRG* : a stereochemical description generator for ligands. *Acta Crystallogr D Struct Biol* **73**, 112–122 (2017).

9. Emsley, P., Lohkamp, B., Scott, W. G. & Cowtan, K. Features and development of Coot. *Acta Crystallographica Section D Biological Crystallography* **66**, 486–501 (2010).

10. Burley, S. K. *et al.* RCSB Protein Data Bank: Powerful new tools for exploring 3D structures of biological macromolecules for basic and applied research and education in fundamental biology, biomedicine, biotechnology, bioengineering and energy sciences. *Nucleic Acids Research* **49**, D437–D451 (2021).

11. Karplus, P. A. & Diederichs, K. Linking Crystallographic Model and Data Quality. *Science* **336**, 1030–1033 (2012).

12. Juanhuix, J. *et al.* Developments in optics and performance at BL13-XALOC, the macromolecular crystallography beamline at the Alba Synchrotron. *J Synchrotron Rad* **21**, 679–689 (2014).

13. Afonine, P. V. *et al.* Towards automated crystallographic structure refinement with phenix.refine. *Acta Crystallographica Section D: Biological Crystallography* **68**, 352–367 (2012).

14. Tanaka, Y. *et al.* New Series of Potent Allosteric Inhibitors of Deoxyhypusine Synthase. *ACS Med. Chem. Lett.* **11**, 1645–1652 (2020).

15. Knight, N. M. L. *et al.* Iridium-Catalysed C(sp^3)−H Activation and Hydrogen Isotope Exchange via Nitrogen-Based Carbonyl Directing Groups. *Adv Synth Catal* **366**, 2577–2586 (2024).

16. Yin, L. *et al.* Novel Imidazol-1-ylmethyl Substituted 1,2,5,6-Tetrahydropyrrolo[3,2,1- *ij*]quinolin-4-ones as Potent and Selective CYP11B1 Inhibitors for the Treatment of Cushing's Syndrome. *J. Med. Chem.* **55**, 6629–6633 (2012).

17. Lucas, S., Negri, M., Heim, R., Zimmer, C. & Hartmann, R. W. Fine-Tuning the Selectivity of Aldosterone Synthase Inhibitors: Structure−Activity and Structure−Selectivity Insights from Studies of Heteroaryl Substituted 1,2,5,6-Tetrahydropyrrolo[3,2,1- *ij*]quinolin-4-one Derivatives. *J. Med. Chem.* **54**, 2307–2319 (2011).

18. Lucas, S., Negri, M., Heim, R., Zimmer, C. & Hartmann, R. W. Fine-Tuning the Selectivity of Aldosterone Synthase Inhibitors: Structure−Activity and Structure−Selectivity Insights from Studies of Heteroaryl Substituted 1,2,5,6-Tetrahydropyrrolo[3,2,1- *ij*]quinolin-4-one Derivatives. *J. Med. Chem.* **54**, 2307–2319 (2011).
