# Supplementary material for: Crystallographic fragment screening supports tool compound discovery and reveals conformational flexibility in human deoxyhypusine synthase
Source: Commun Chem. 2026 Jan 17;9:66. doi: 10.1038/s42004-026-01897-9 (PMC12868627; doi:10.1038/s42004-026-01897-9)
Supplement: Supplementary file 3 — Description of Additional Supplementary Files [file 42004_2026_1897_MOESM3_ESM.pdf]

Description of Additional Supplementary Files:

File name: Supplementary Movie 1

Description: Local flexibility of the apo DHS

File name: Supplementary Movie 2

Description: Local flexibility of the NAD-bound DHS

File name: Supplementary Data 1

Description: Composition of the fragment library used

File name: Supplementary Data 2

Description: Visualization of each hit with all its binding poses along with their surrounding maps

File name: Supplementary Data 3

Description: Summary of data reduction and structure refinement statistics

File name: Supplementary Data 4

Description: PyMOL session with all identified hits
