# Supplementary material for: Crystallographic fragment screening supports tool compound discovery and reveals conformational flexibility in human deoxyhypusine synthase
Source: Commun Chem. 2026 Jan 17;9:66. doi: 10.1038/s42004-026-01897-9 (PMC12868627; doi:10.1038/s42004-026-01897-9)

Supplementary Data File 2: All hits identified in the CFS campaign.

List of all hits identified in the here-described campaign with one type of ligand per page. Ligand ID as in supplementary table 2 and its 2D representation is provided on top of each page. Below all ligands modelled in a given structure are depicted with colouring of carbon atoms indicating hit population classification (yellow - active site, brown - entrance, orange - ball-and-chain plum - interface, green - peripherial and black indicating molecule from the symmetry related dimer). For each molecule three sets of maps are depicted as mesh around the molecule - ground (PanDDA ground state average map), native (crystallographic blue 2fo-fc and green/red fo-fc) and PanDDA (green/red Z-map and blue event map). Core of the protein is traced as tube and neighbouring sidechains shown as sticks.

VT00015

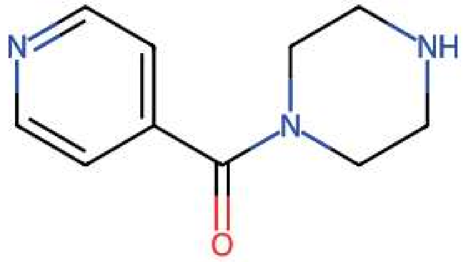

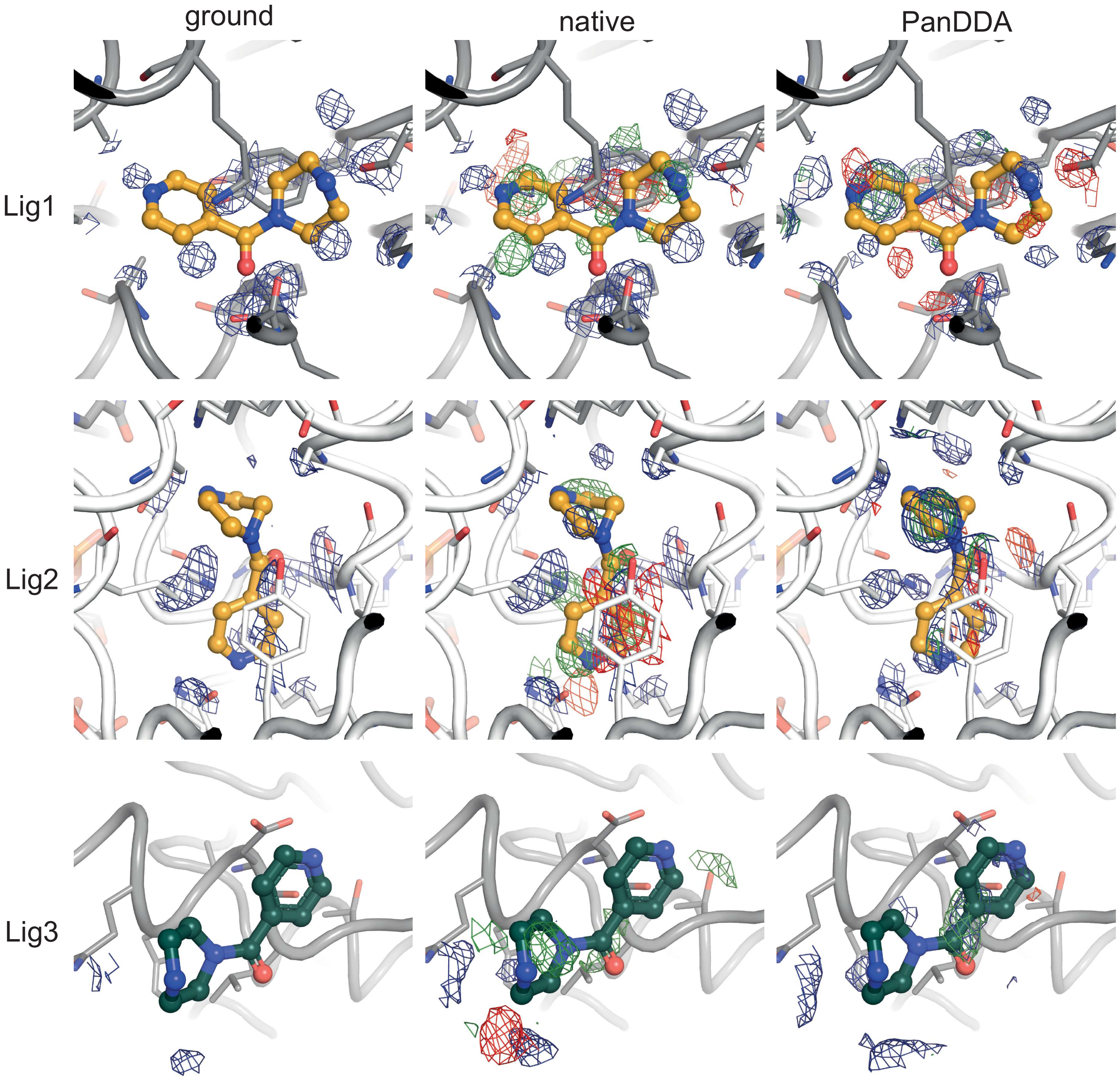

VT00019

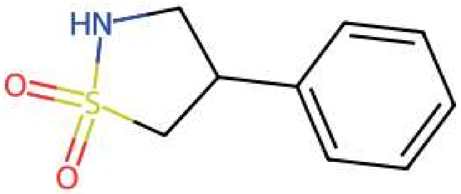

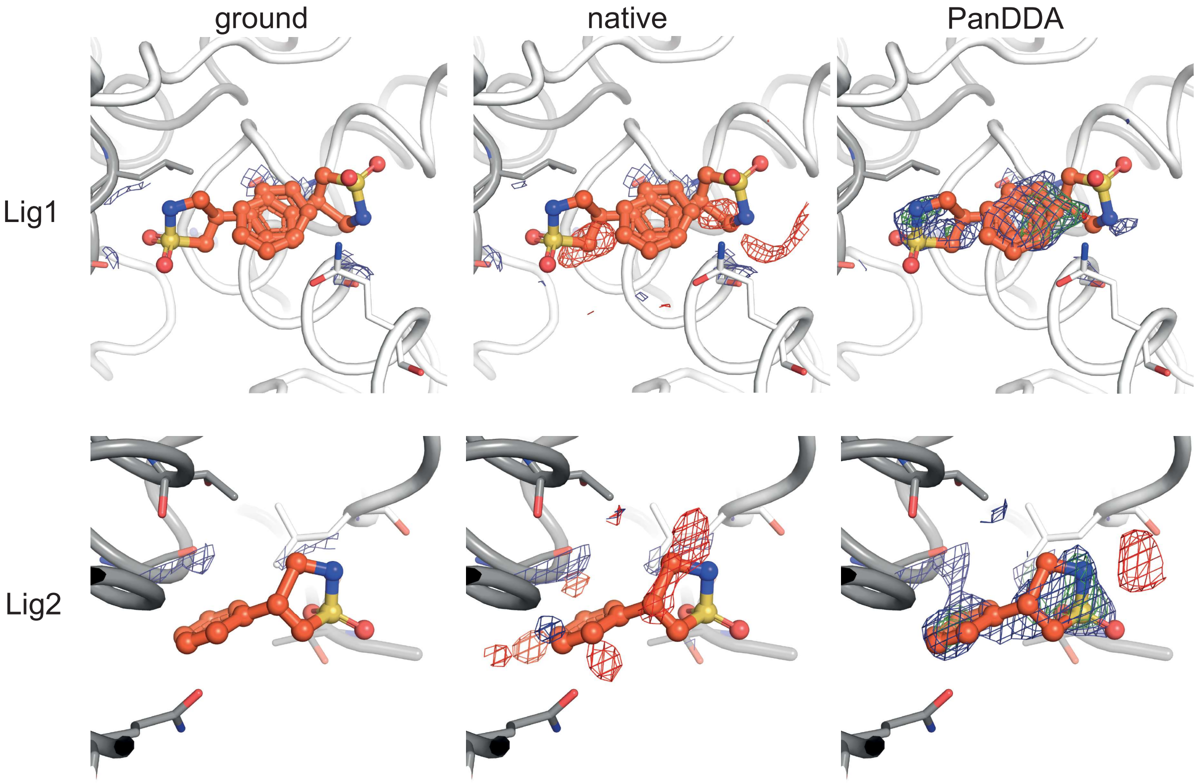

VT00025

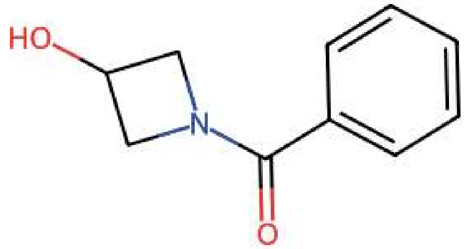

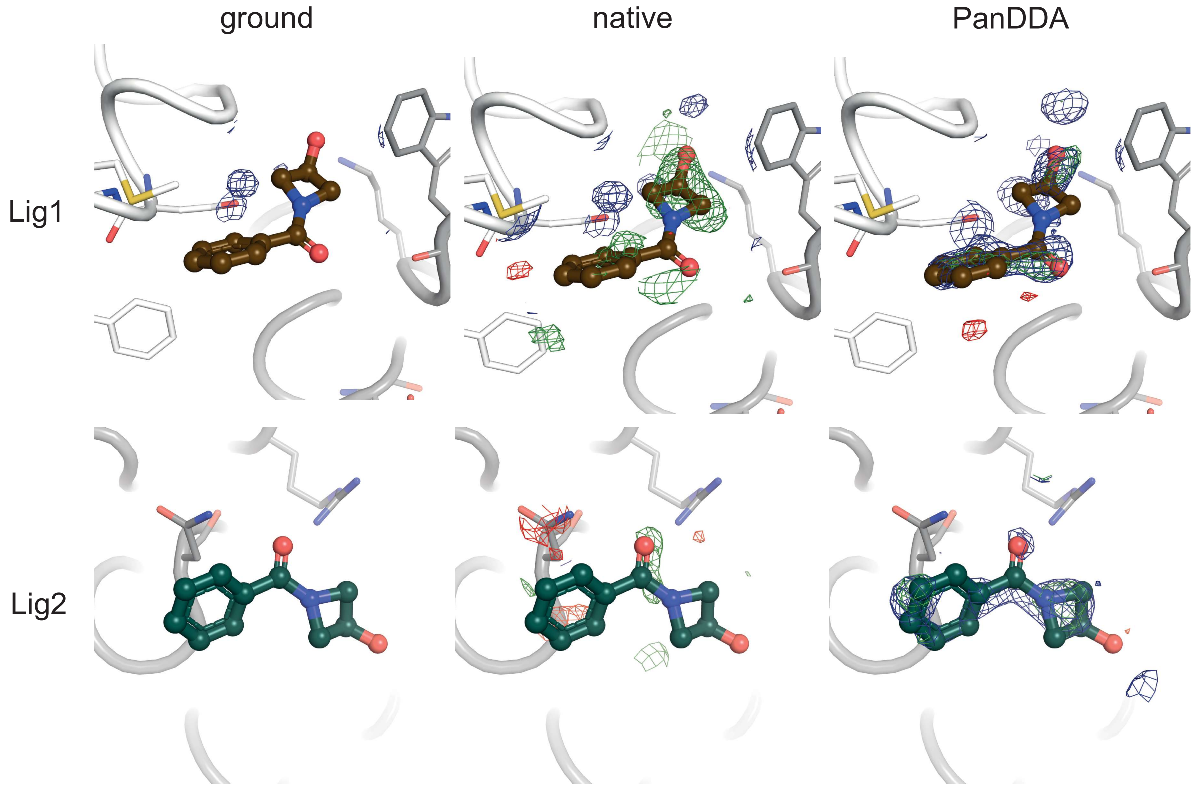

VT00038

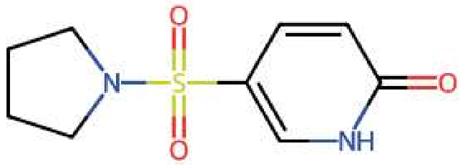

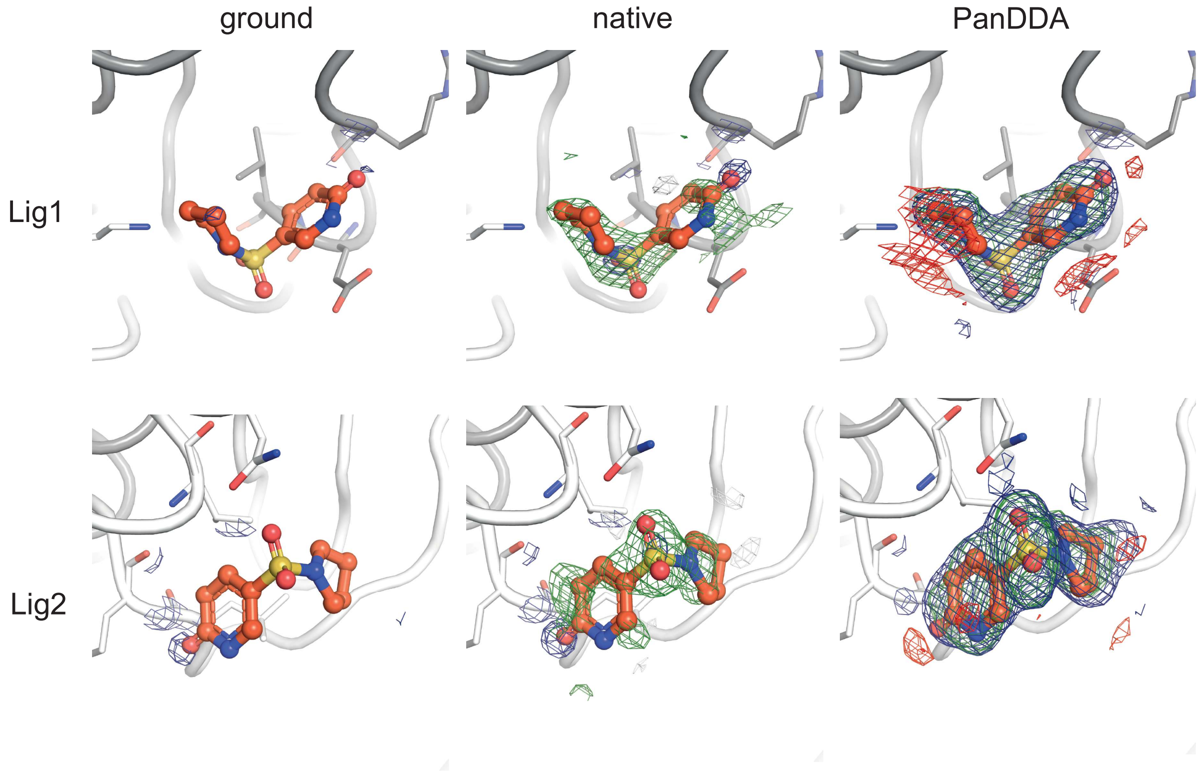

VT00048

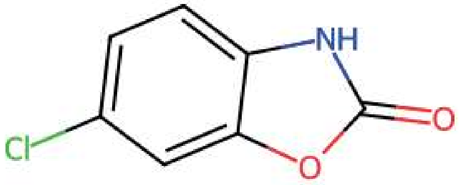

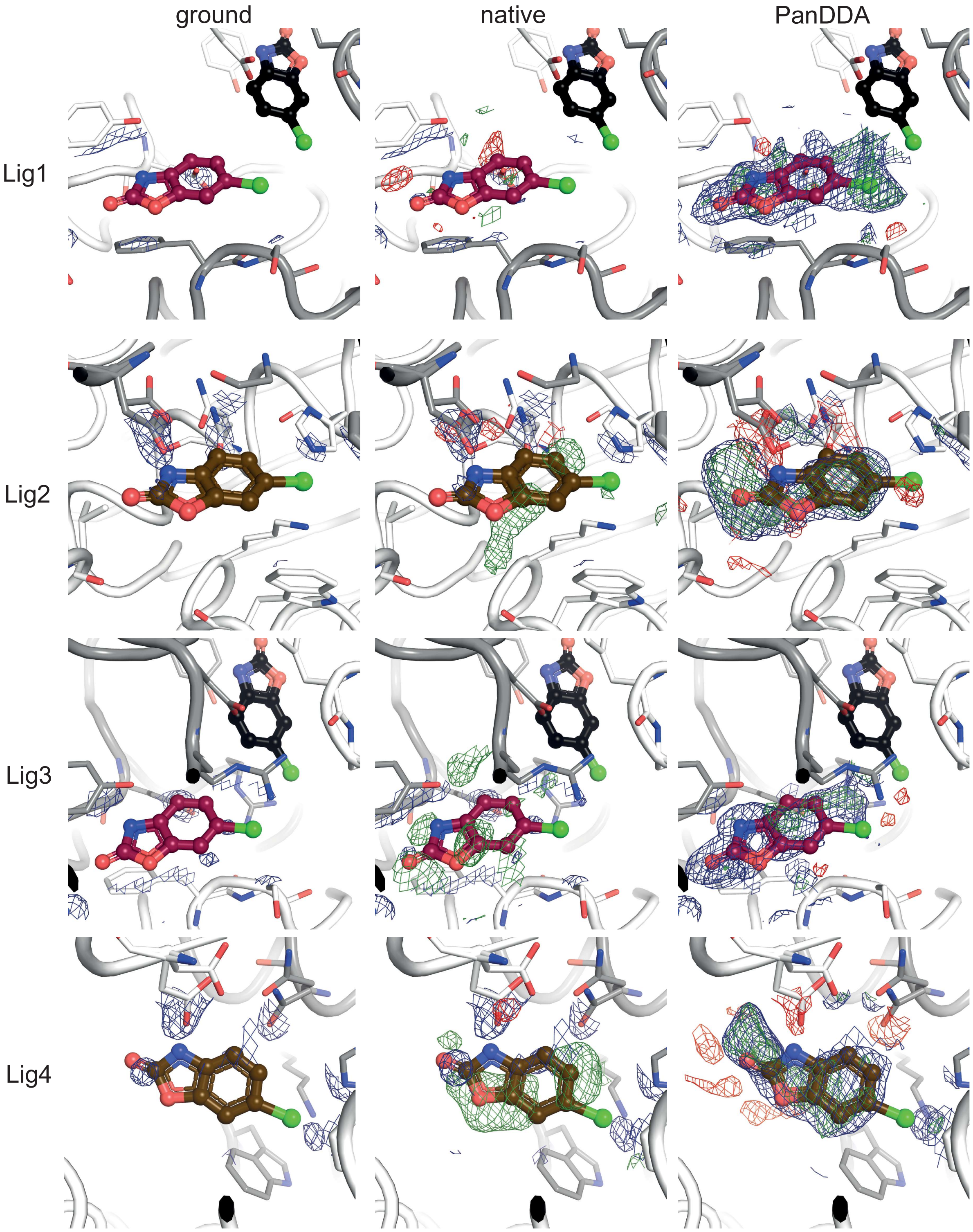

VT00049

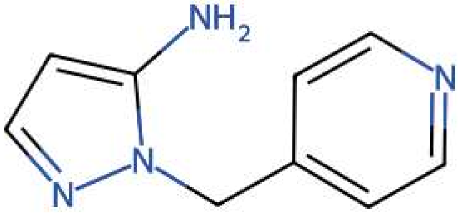

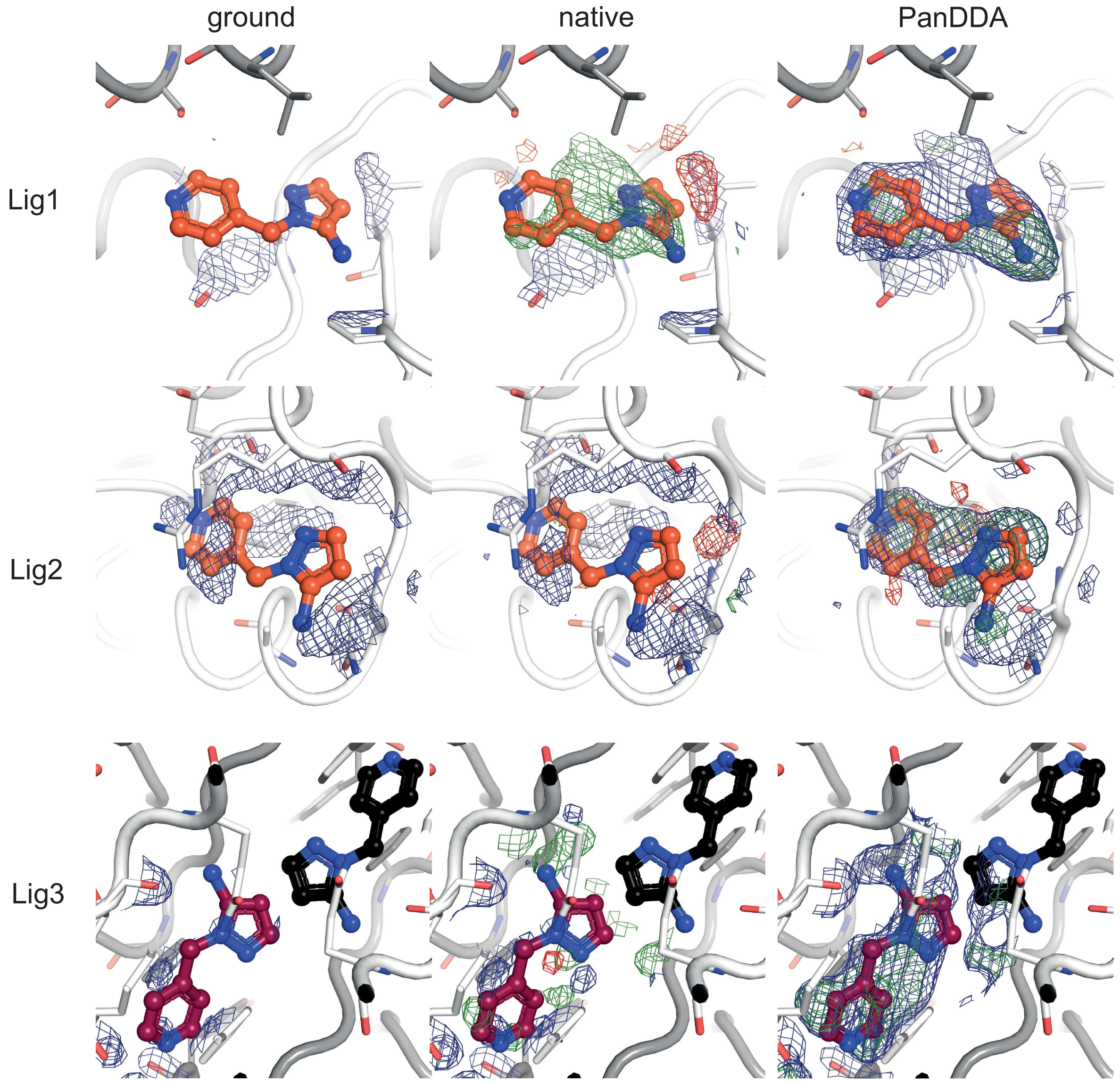

VT00058

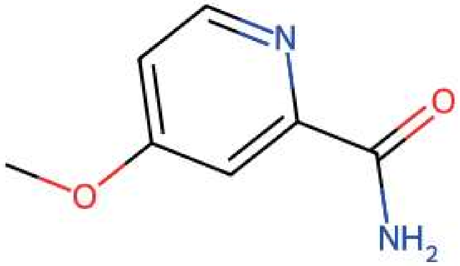

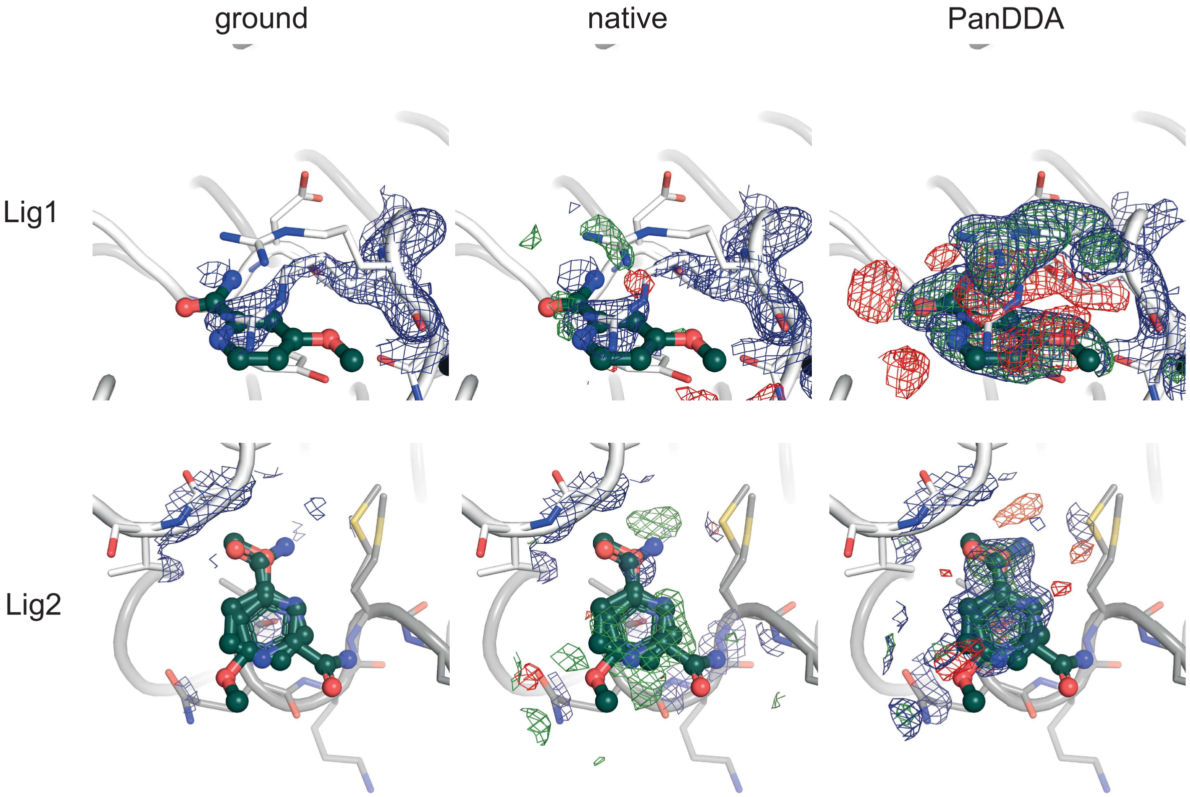

VT00065

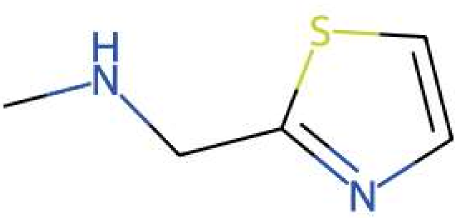

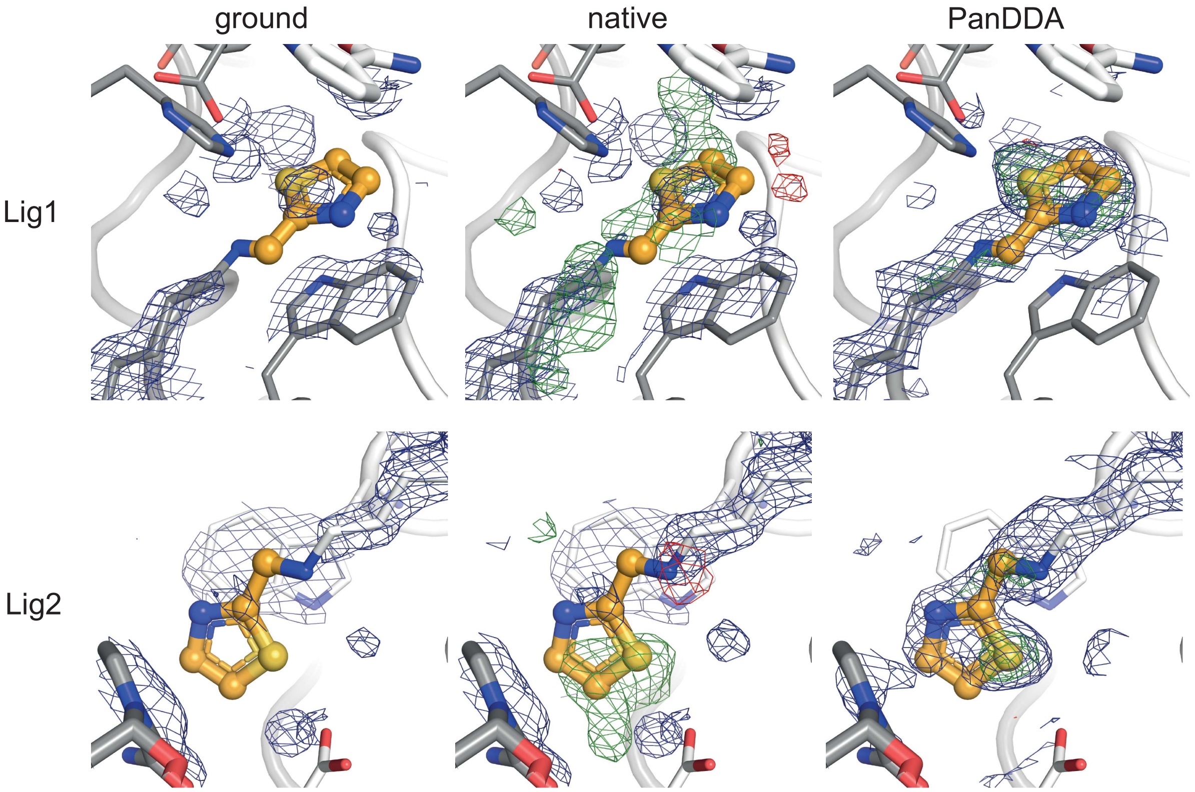

VT00068

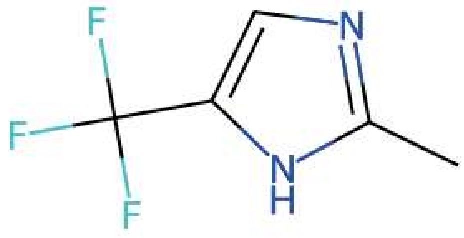

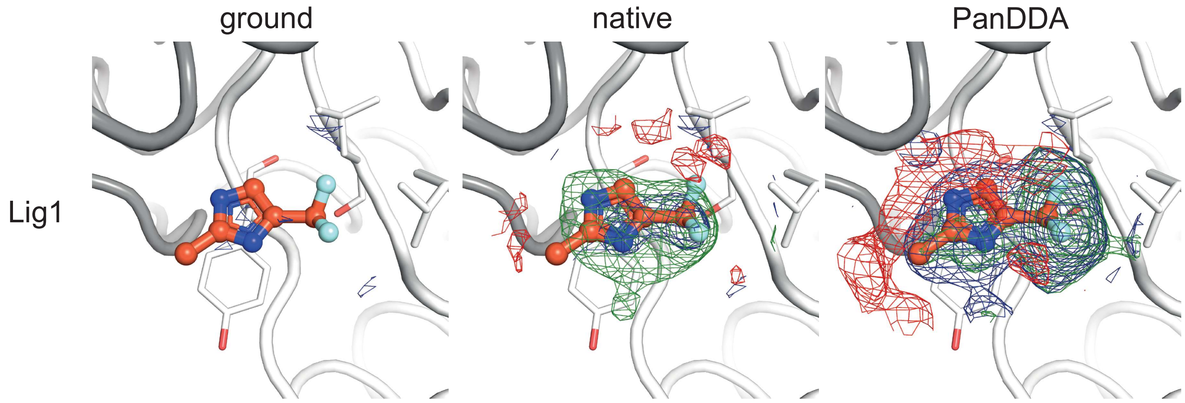

VT00079

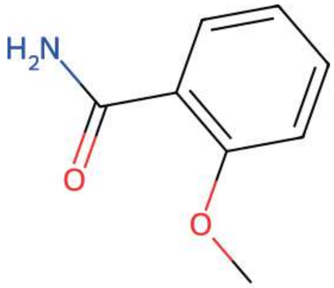

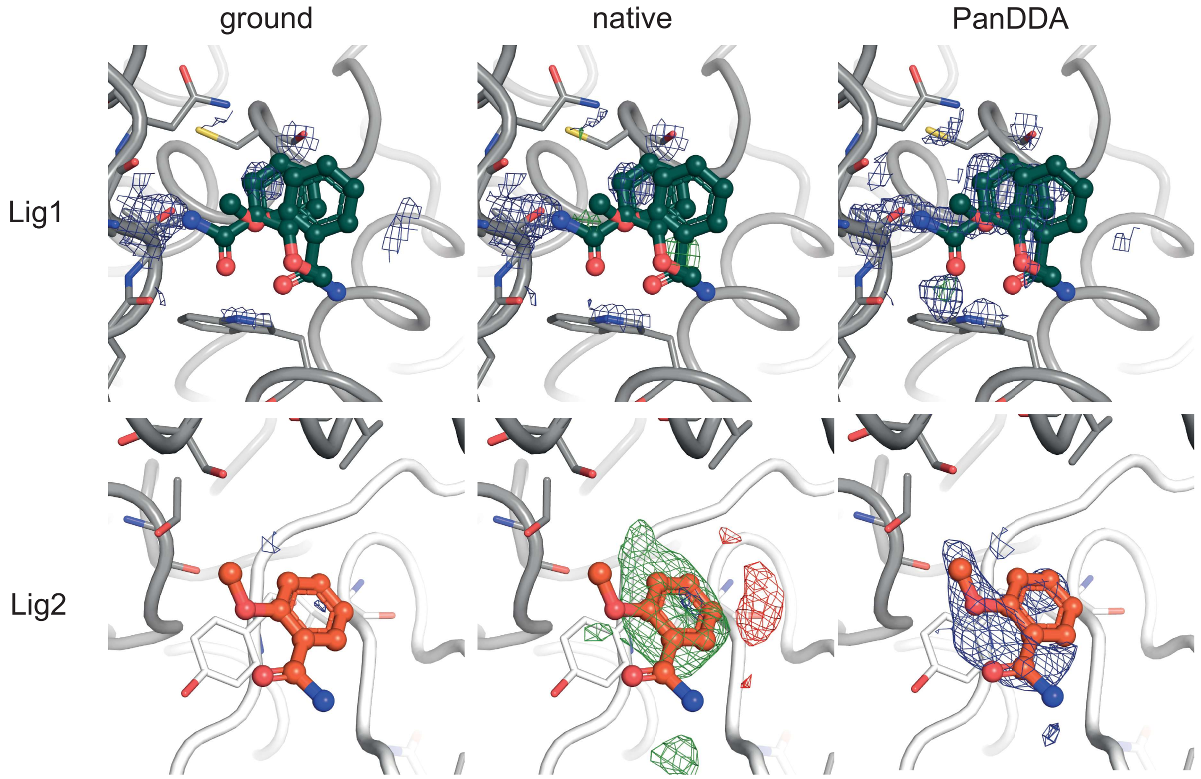

VT00082

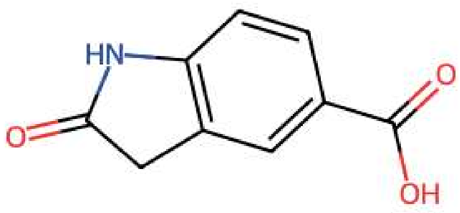

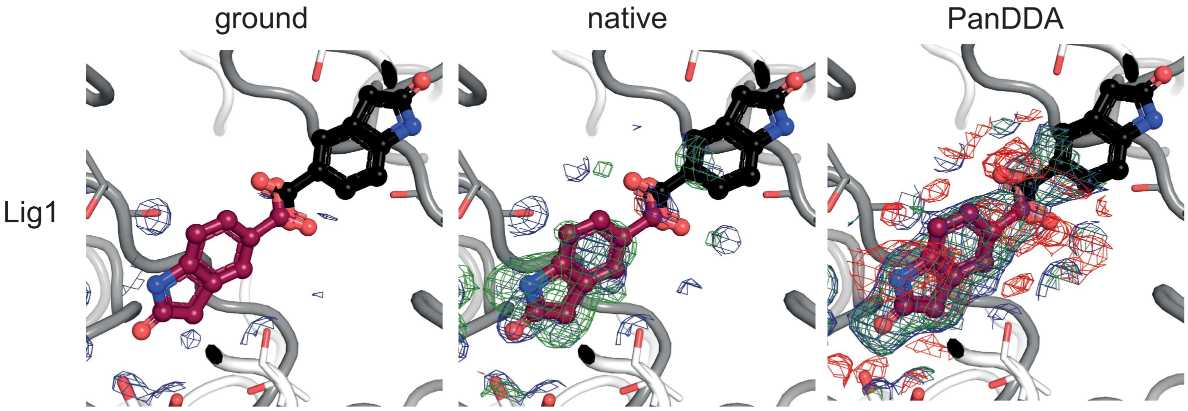

VT00086

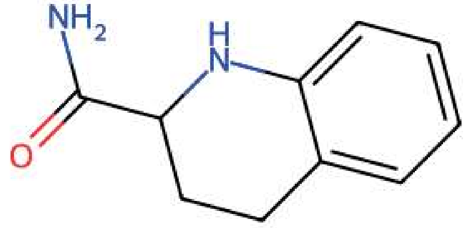

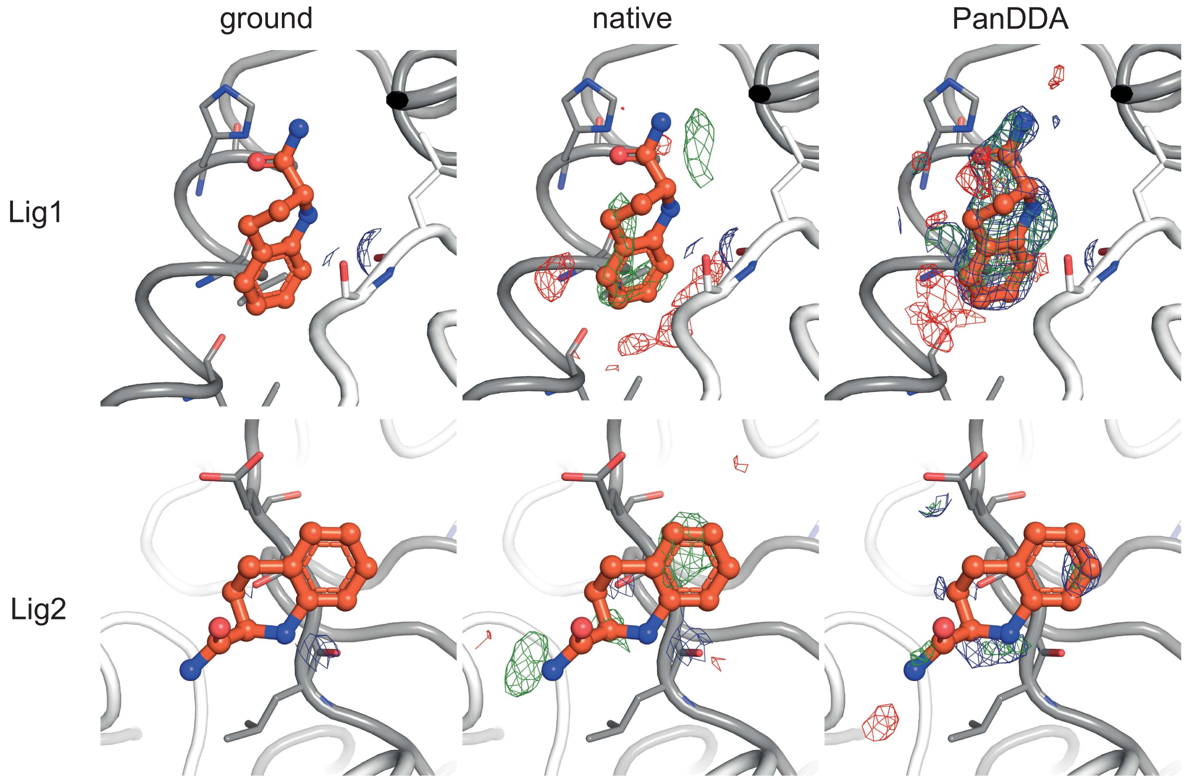

VT00089

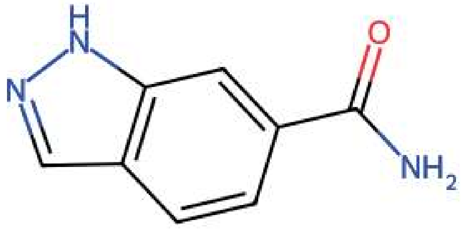

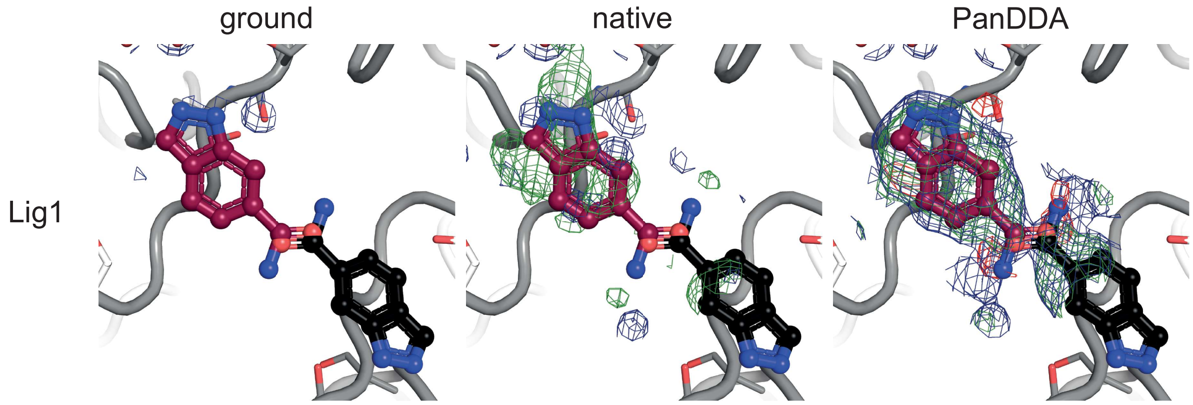

VT00096

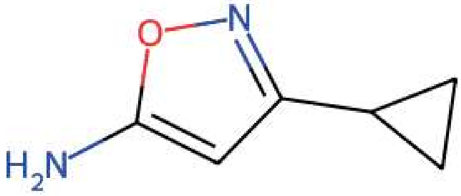

ground native PanDDA

Lig1 & Lig2

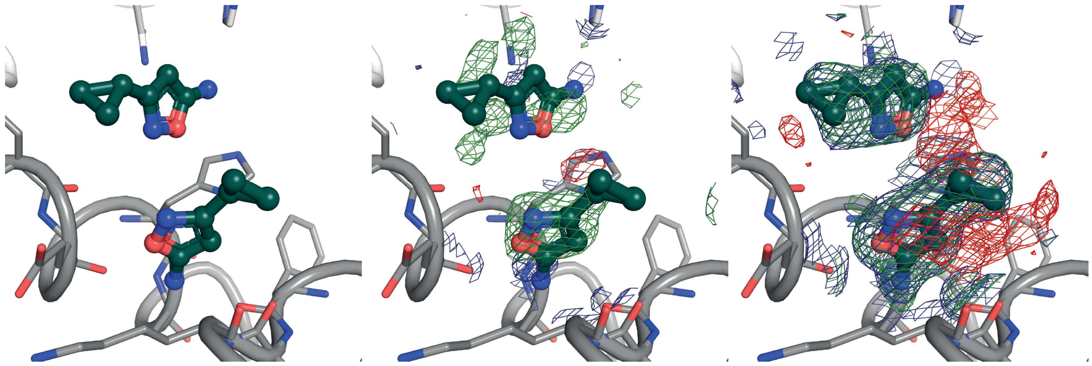

VT00127

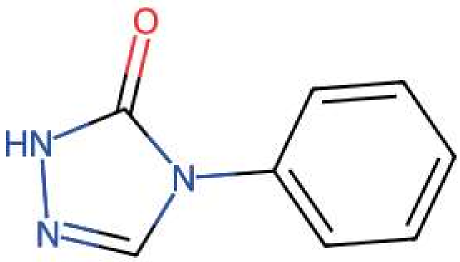

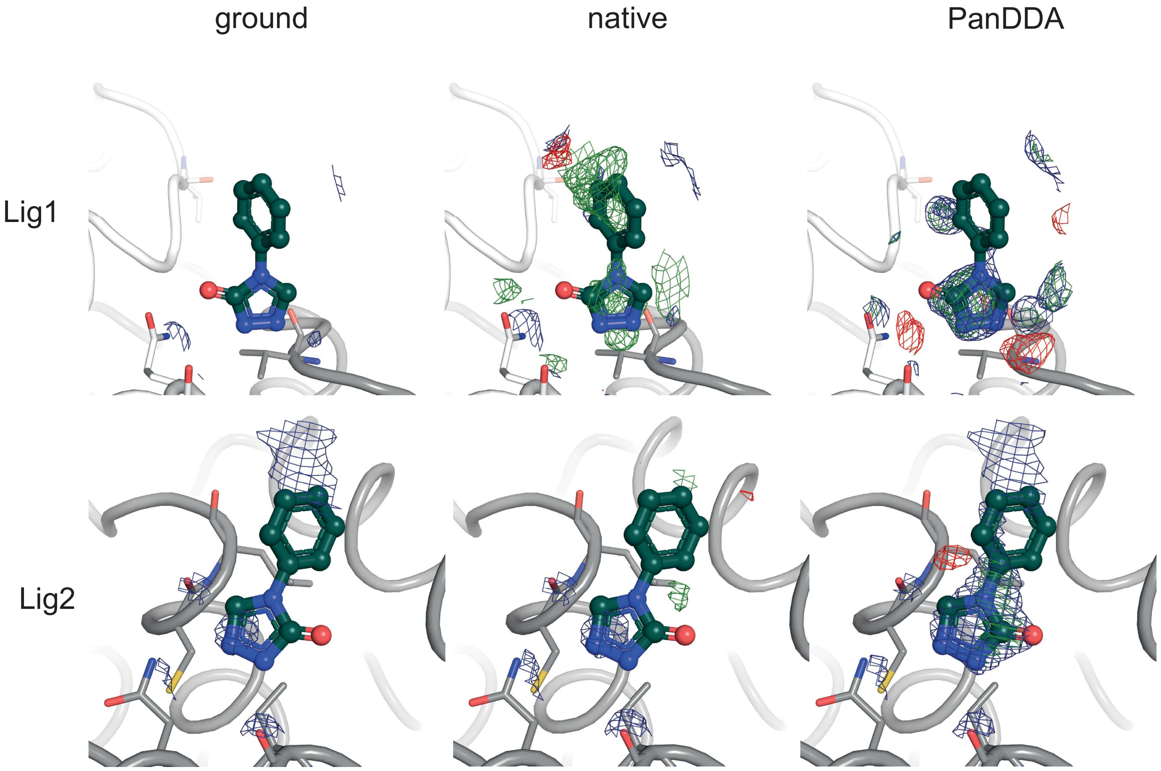

VT00128

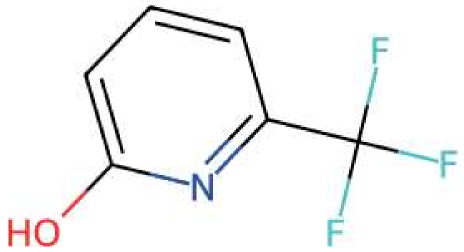

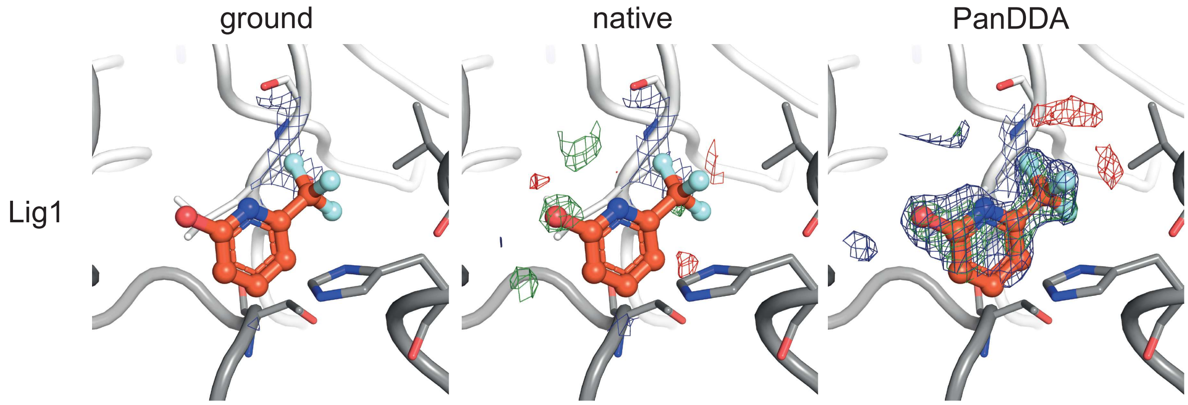

VT00143

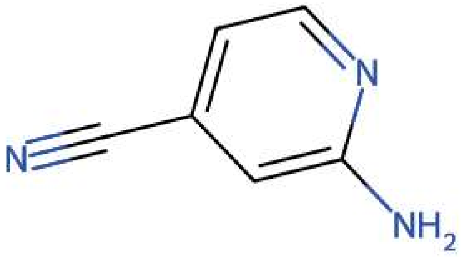

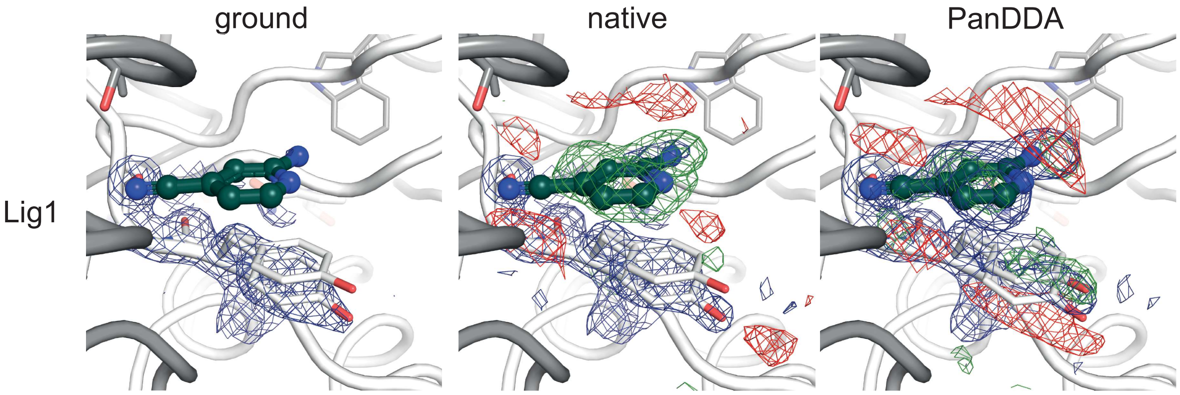

VT00154

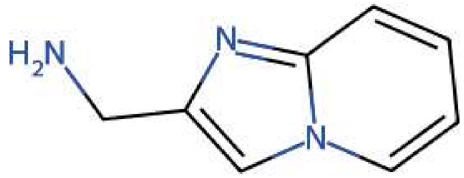

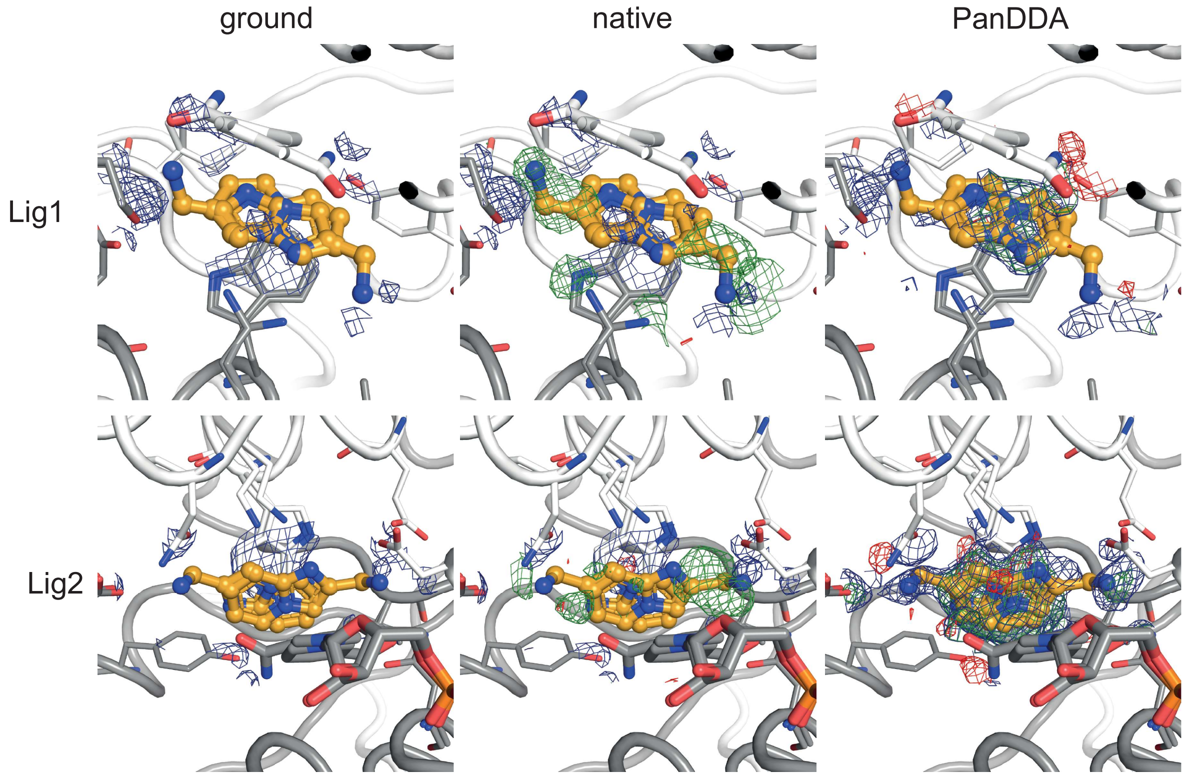

VT00155

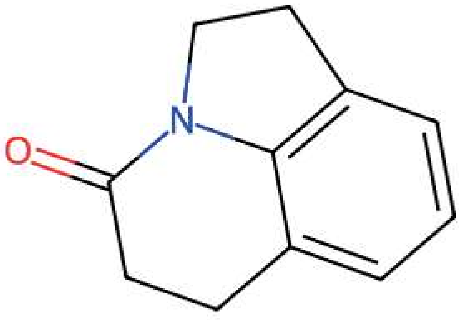

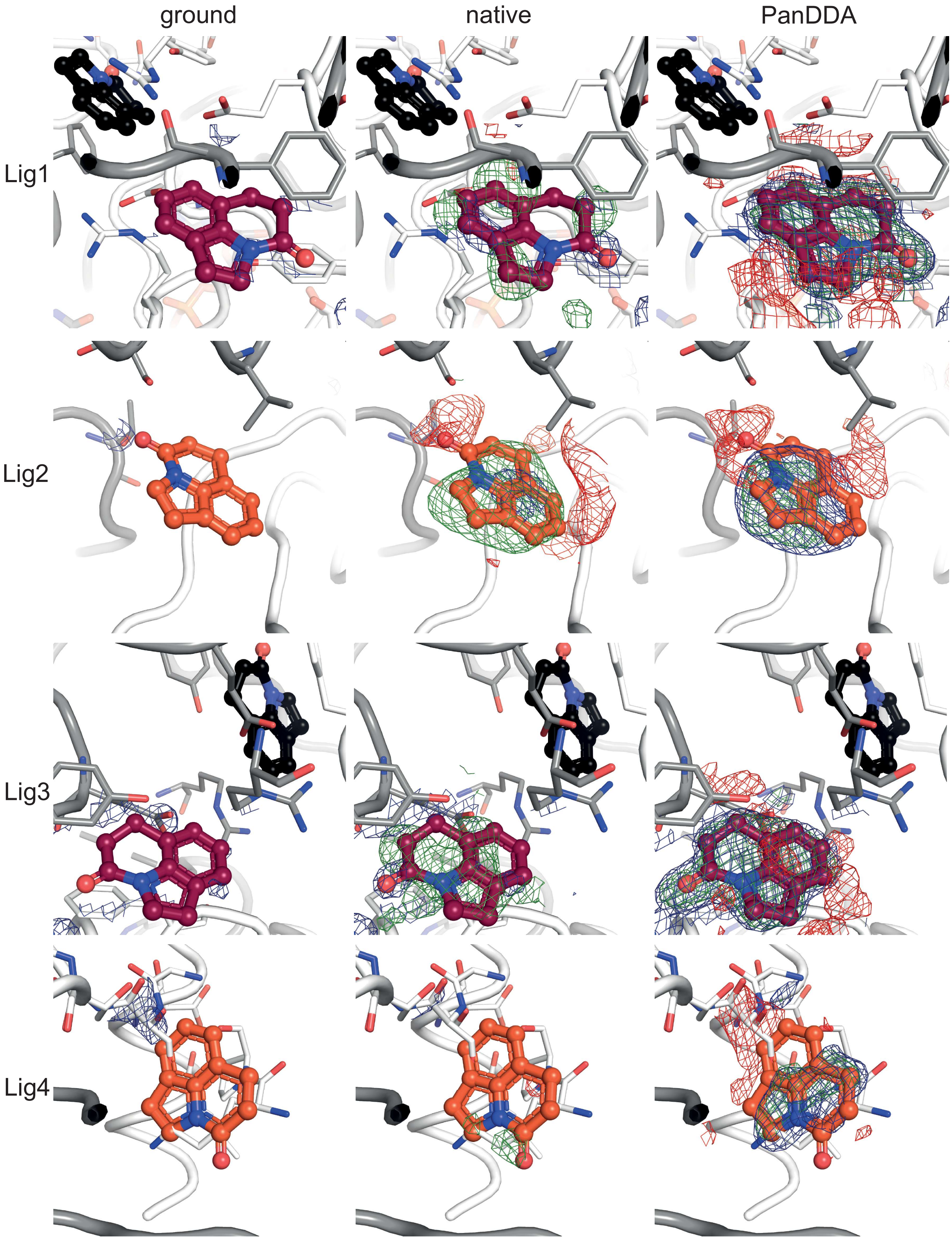

VT00165

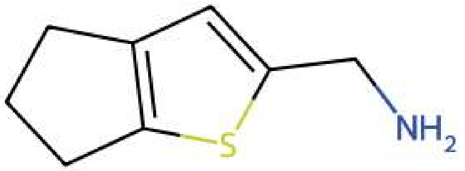

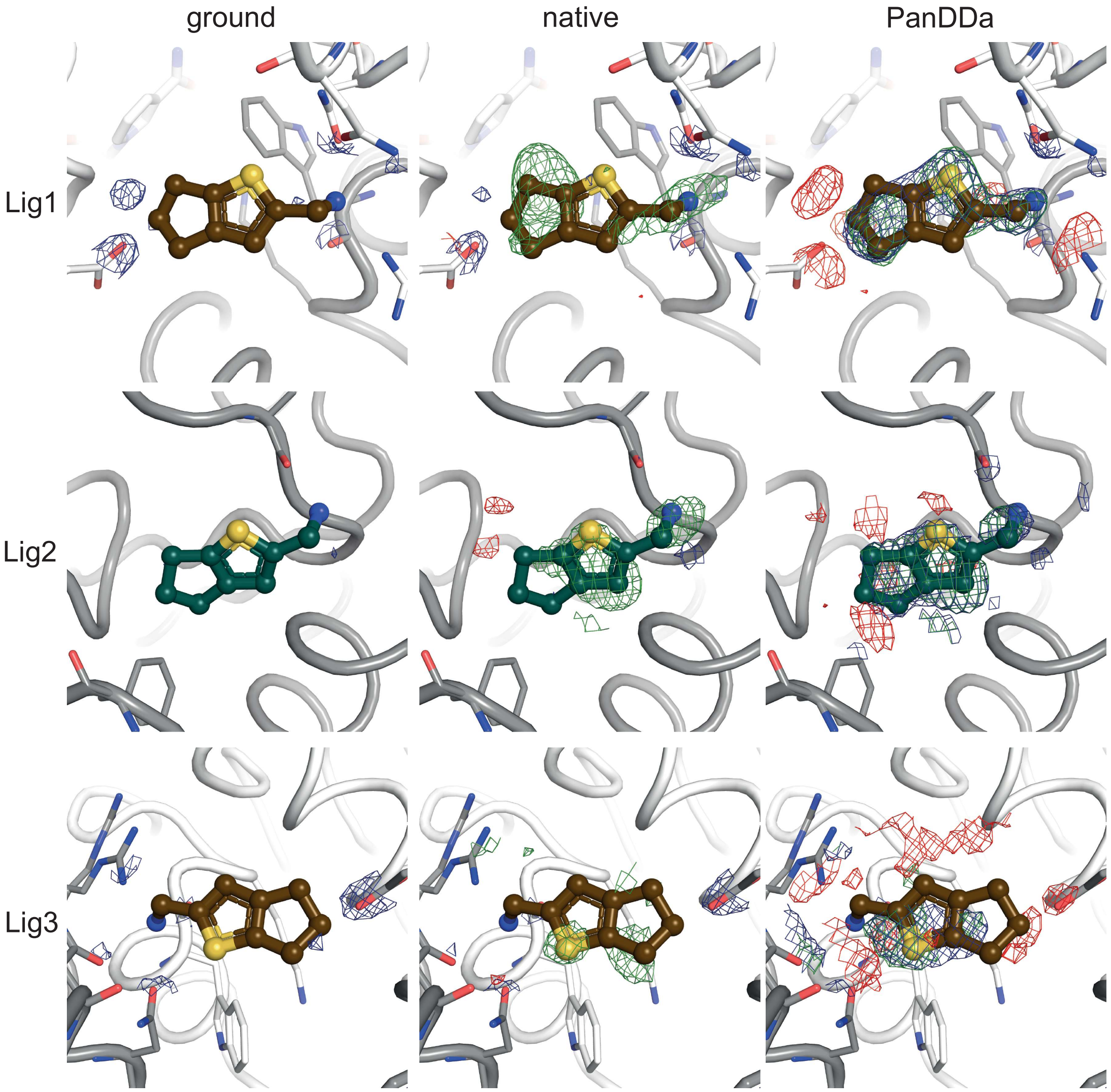

VT00173

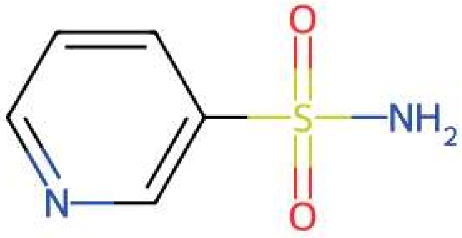

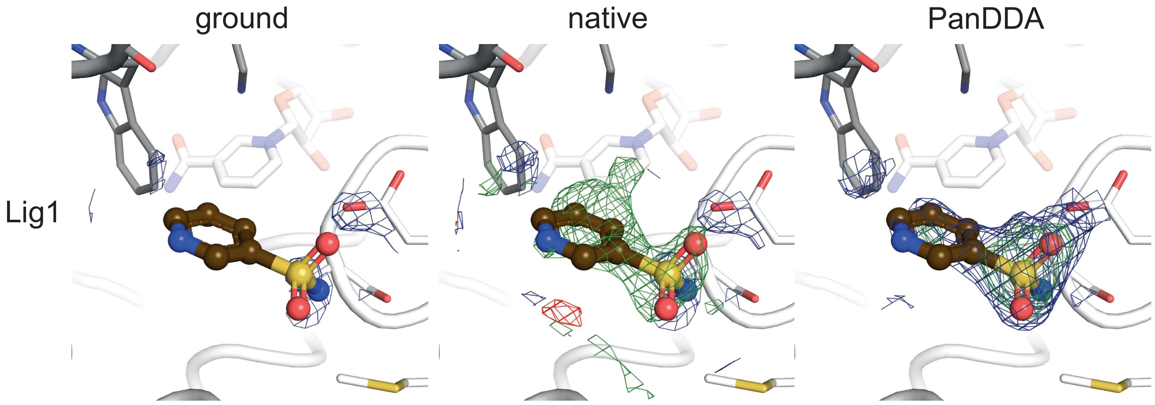

VT00175

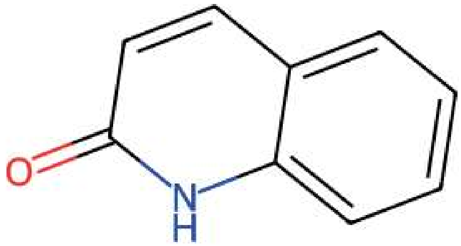

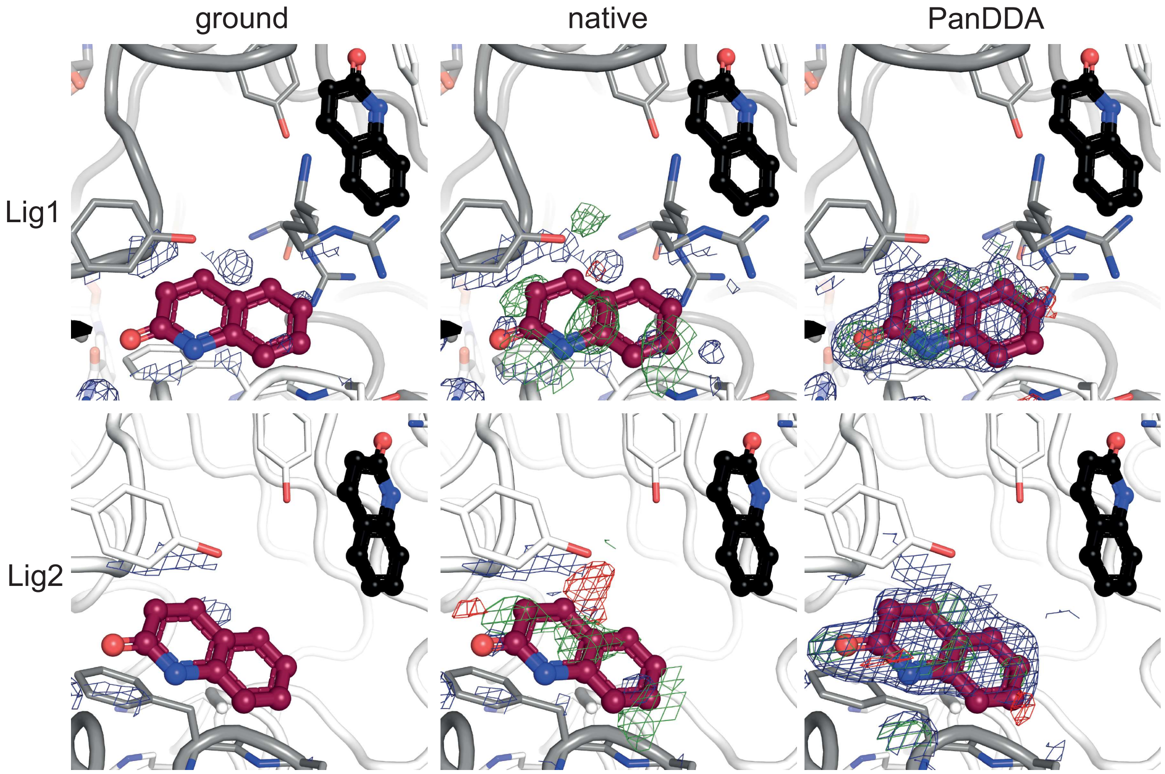

VT00178

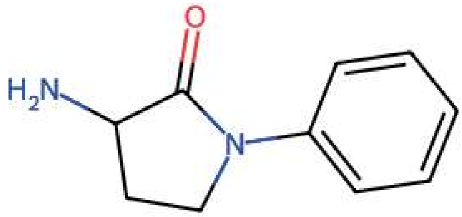

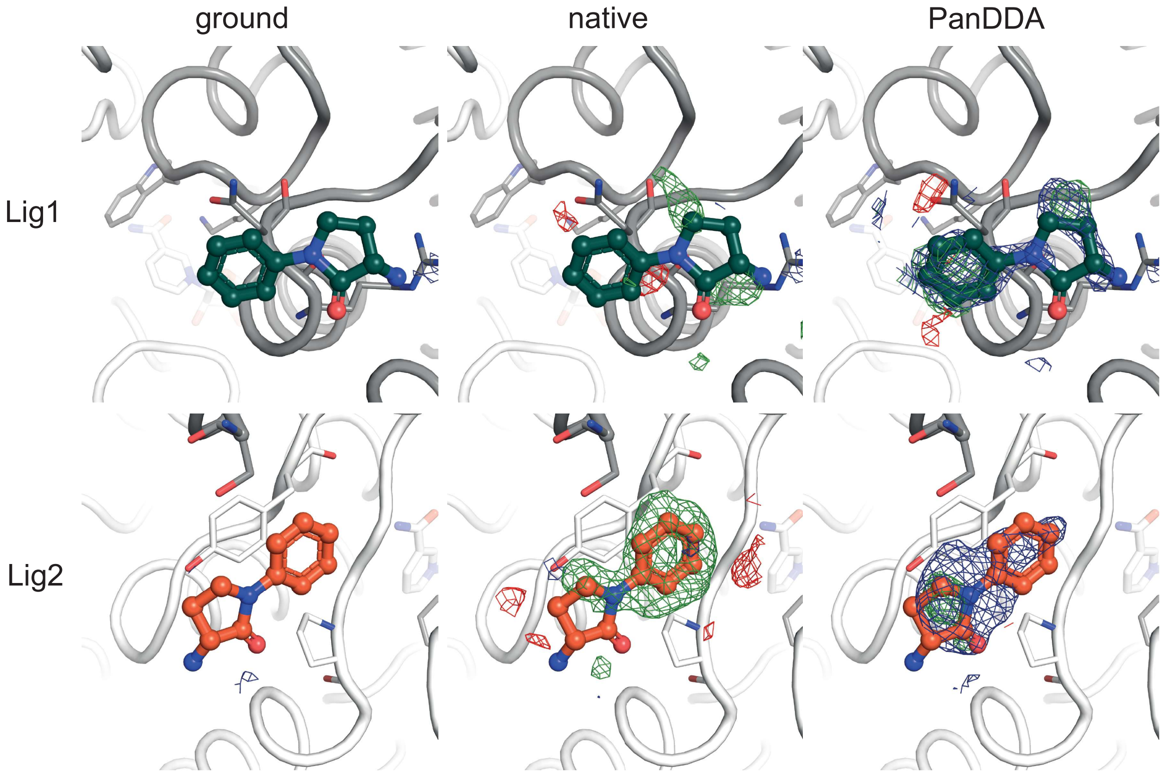

VT00188

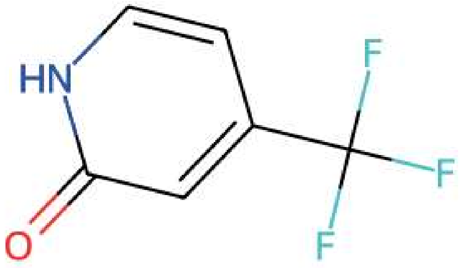

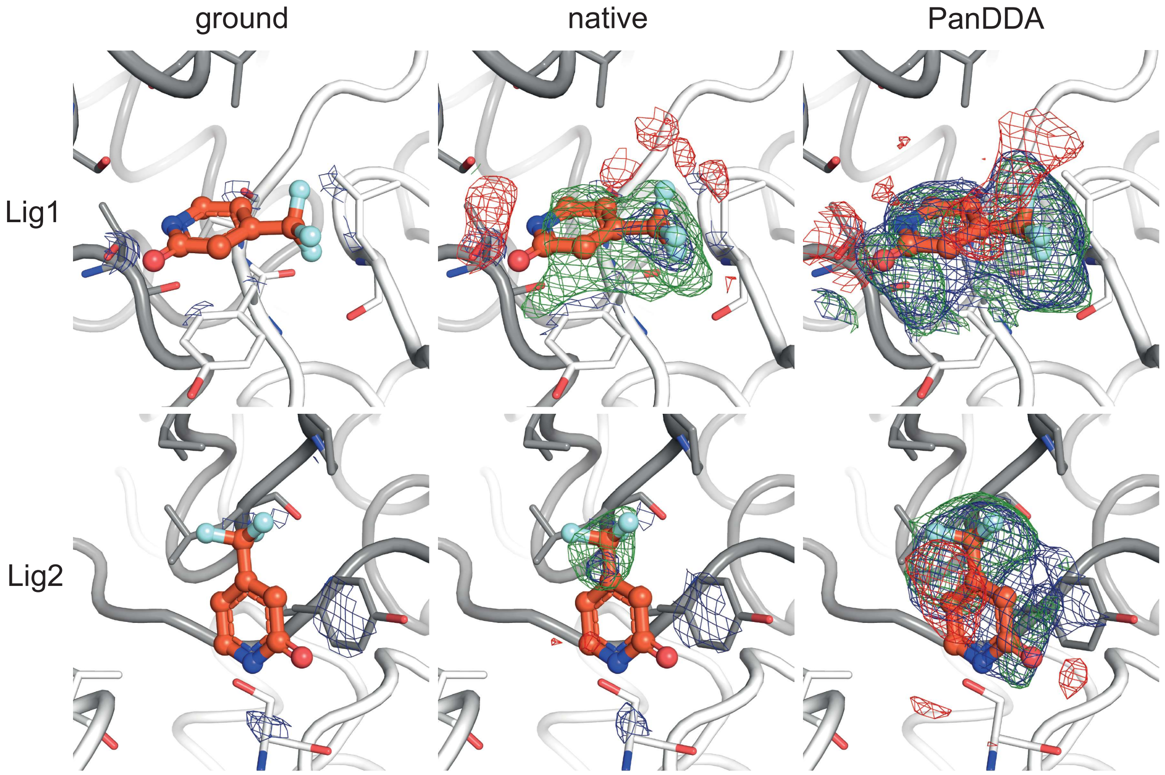

VT00190

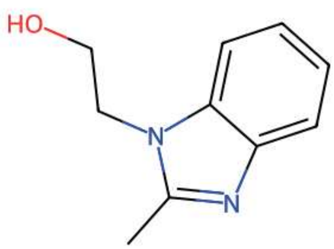

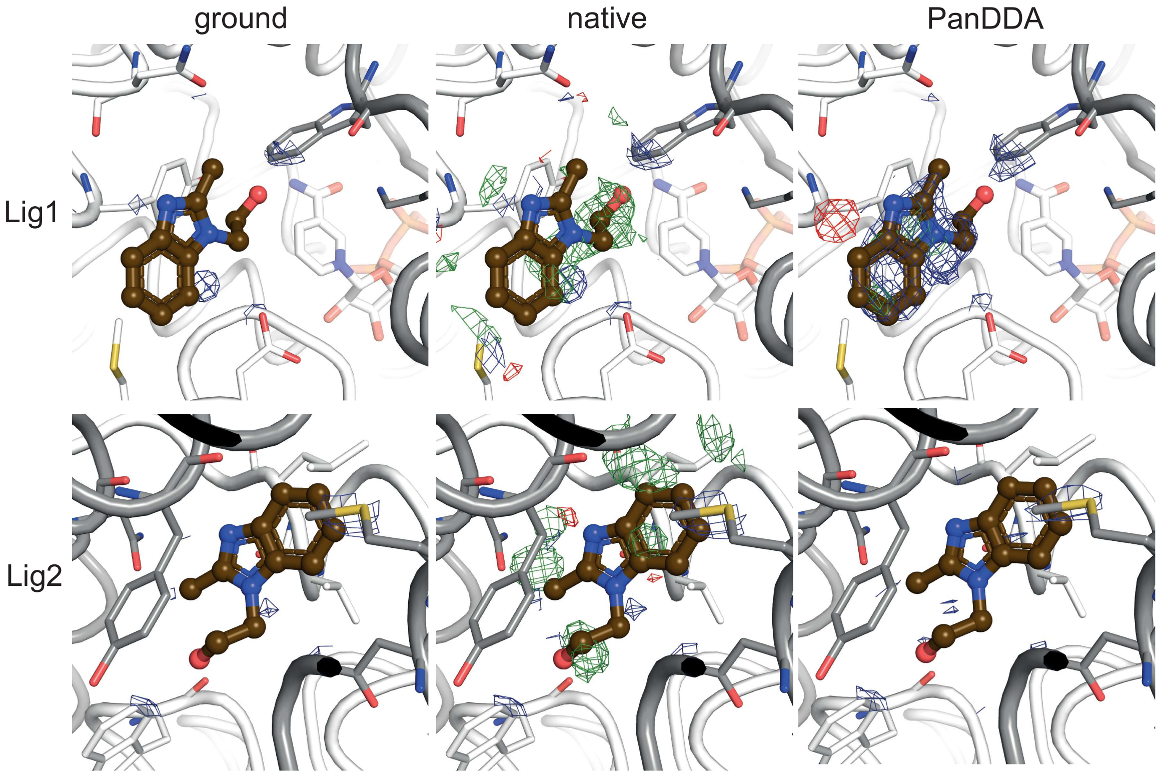

VT00193

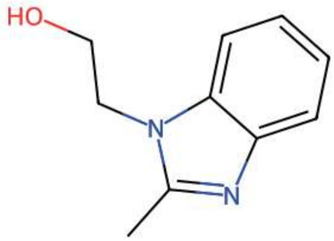

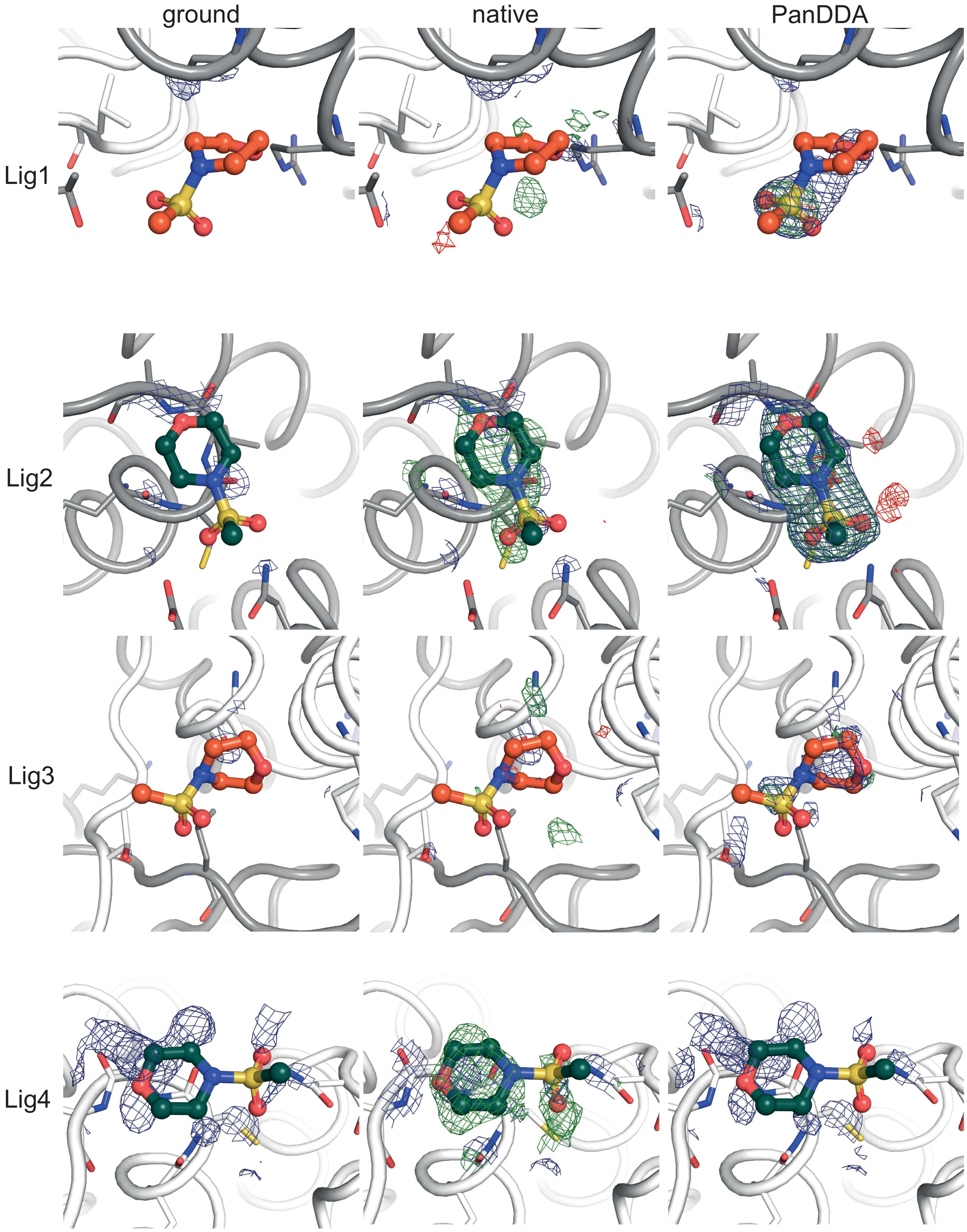

VT00204

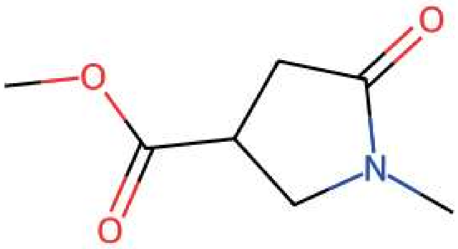

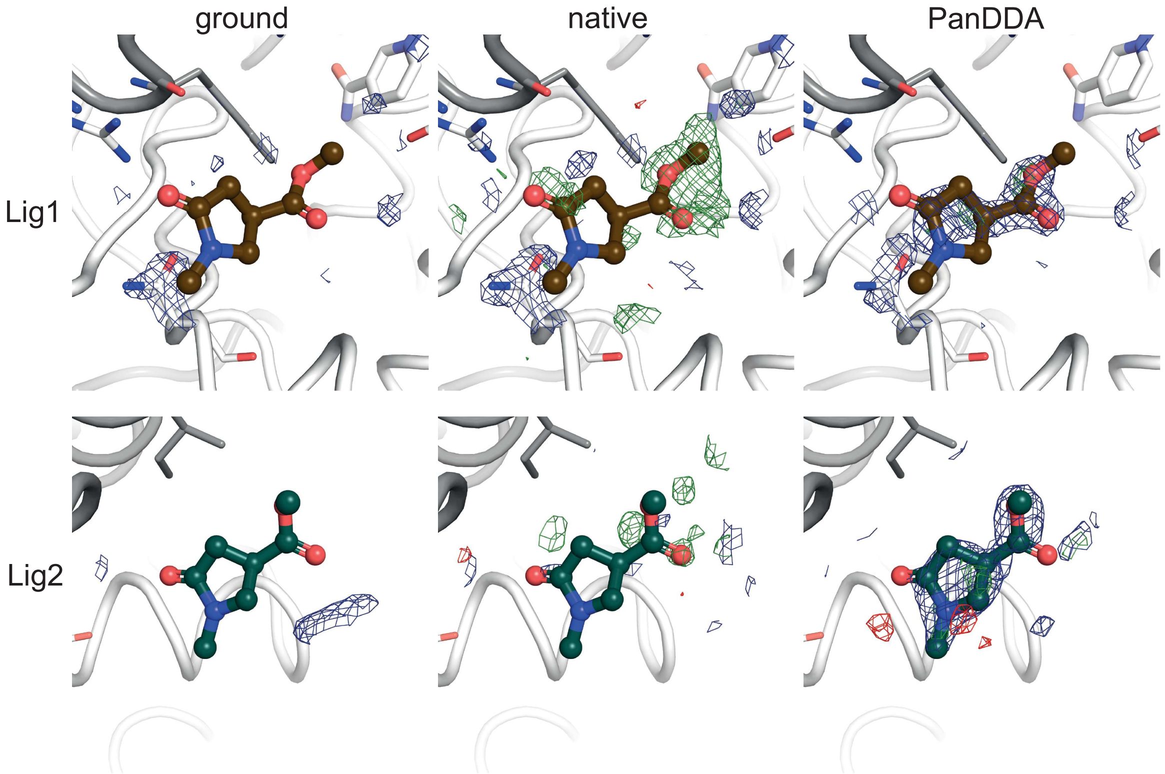

VT00210

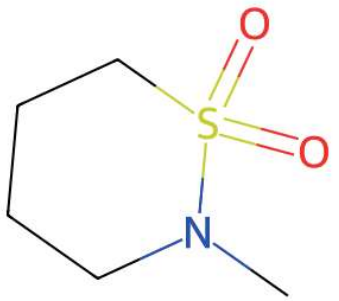

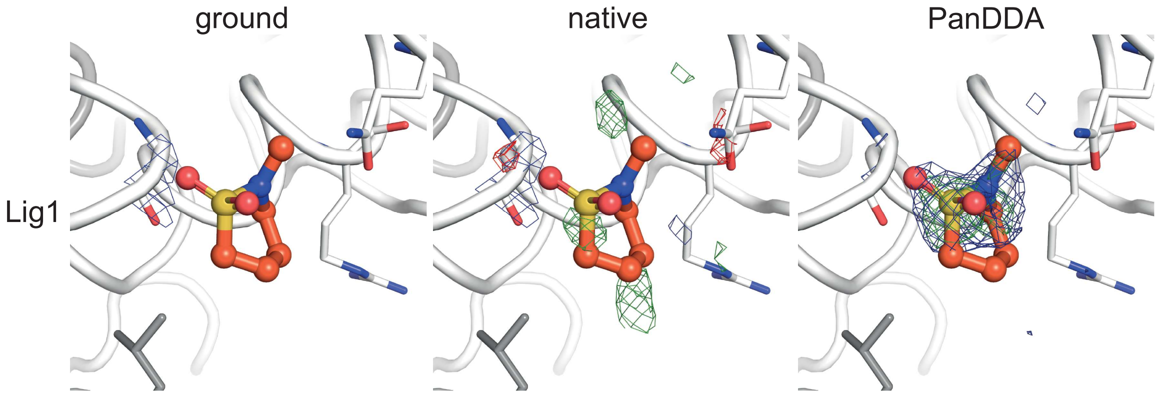

VT00213

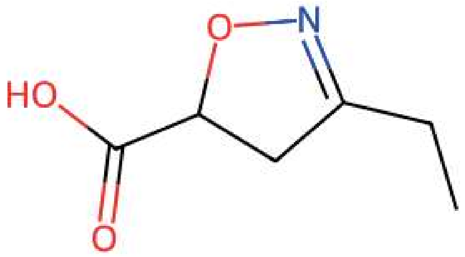

ground native PanDDA

Lig1

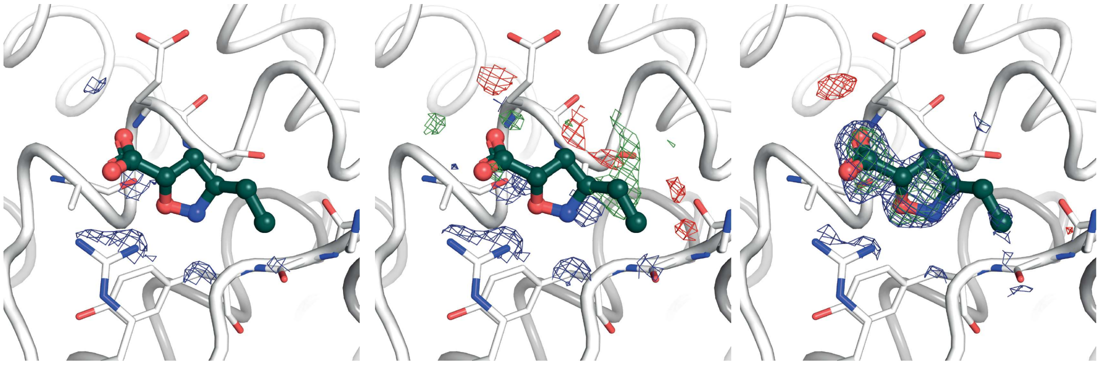

VT00215

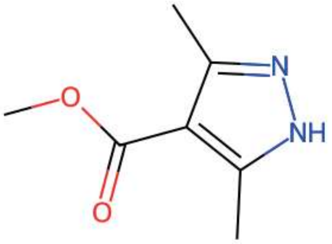

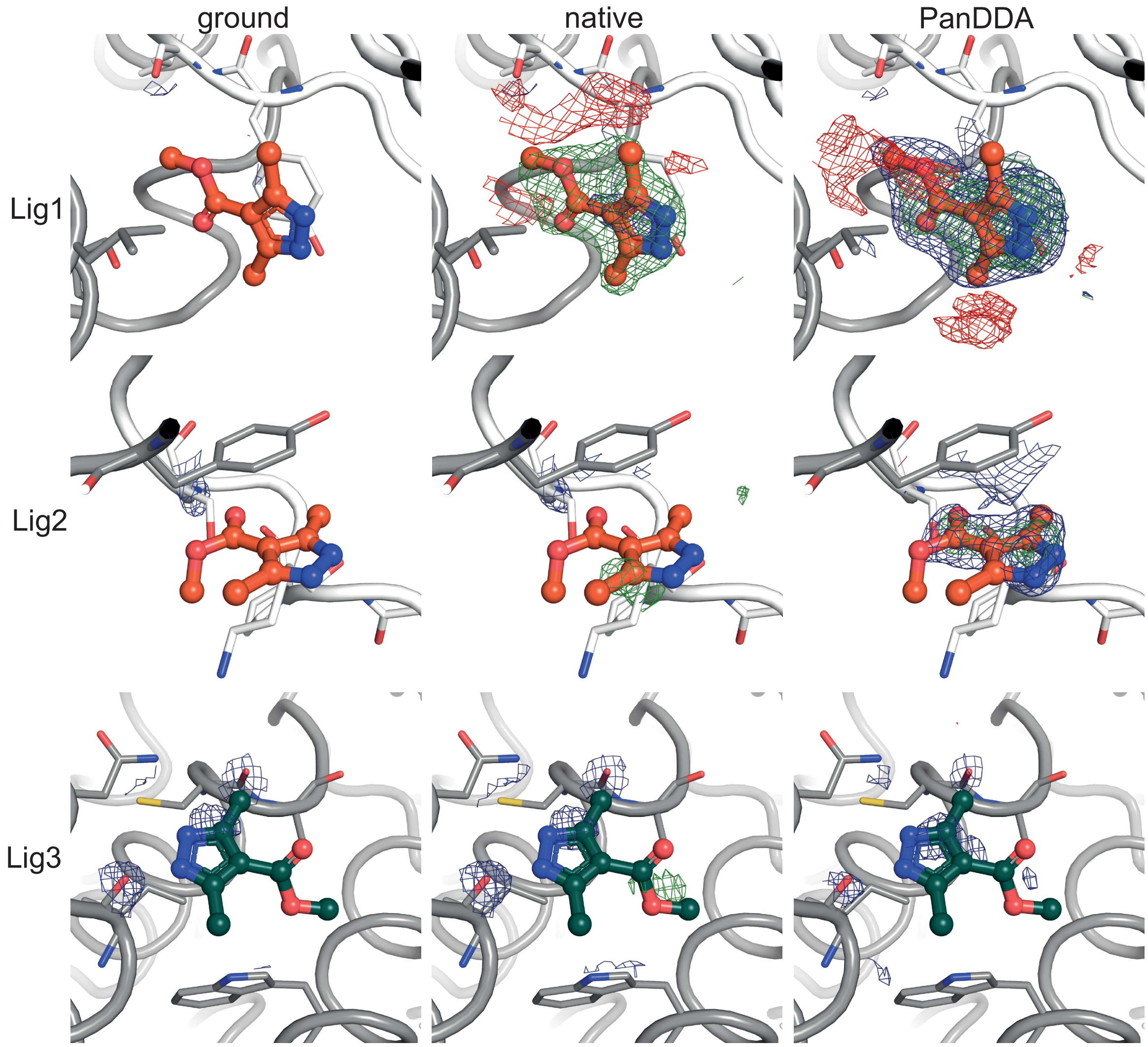

VT00216

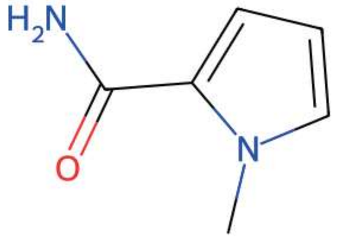

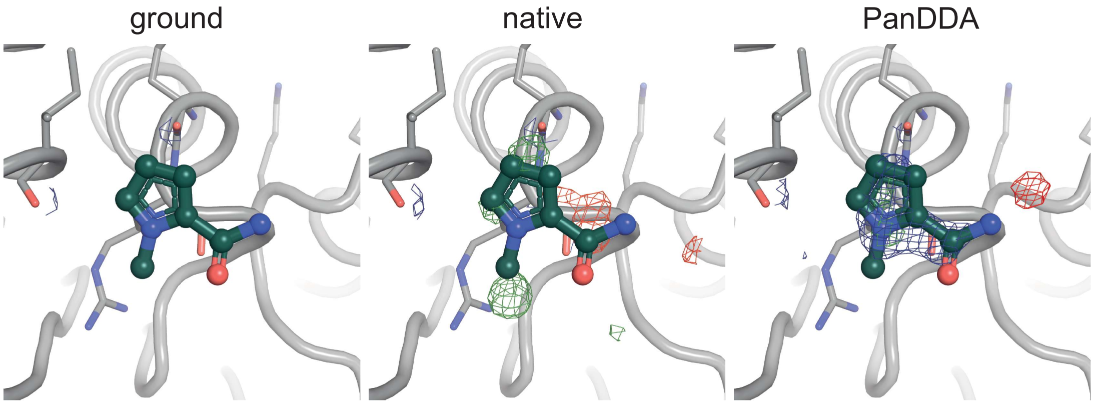

Lig4

VT00217

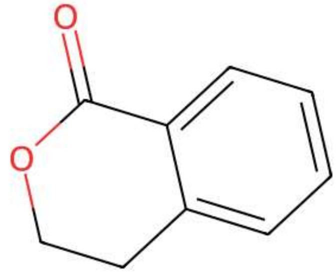

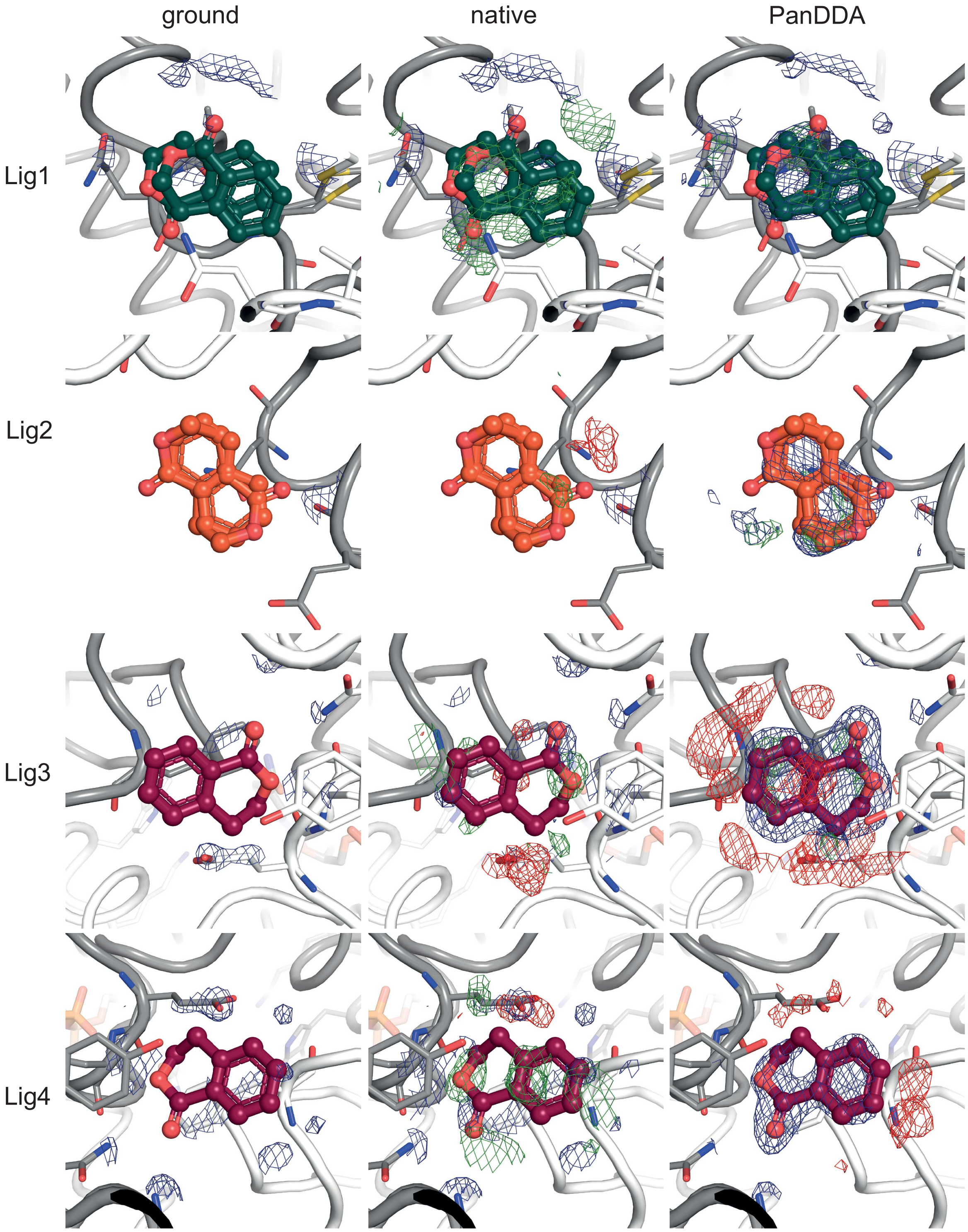

VT00218

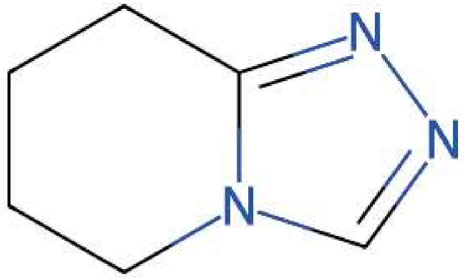

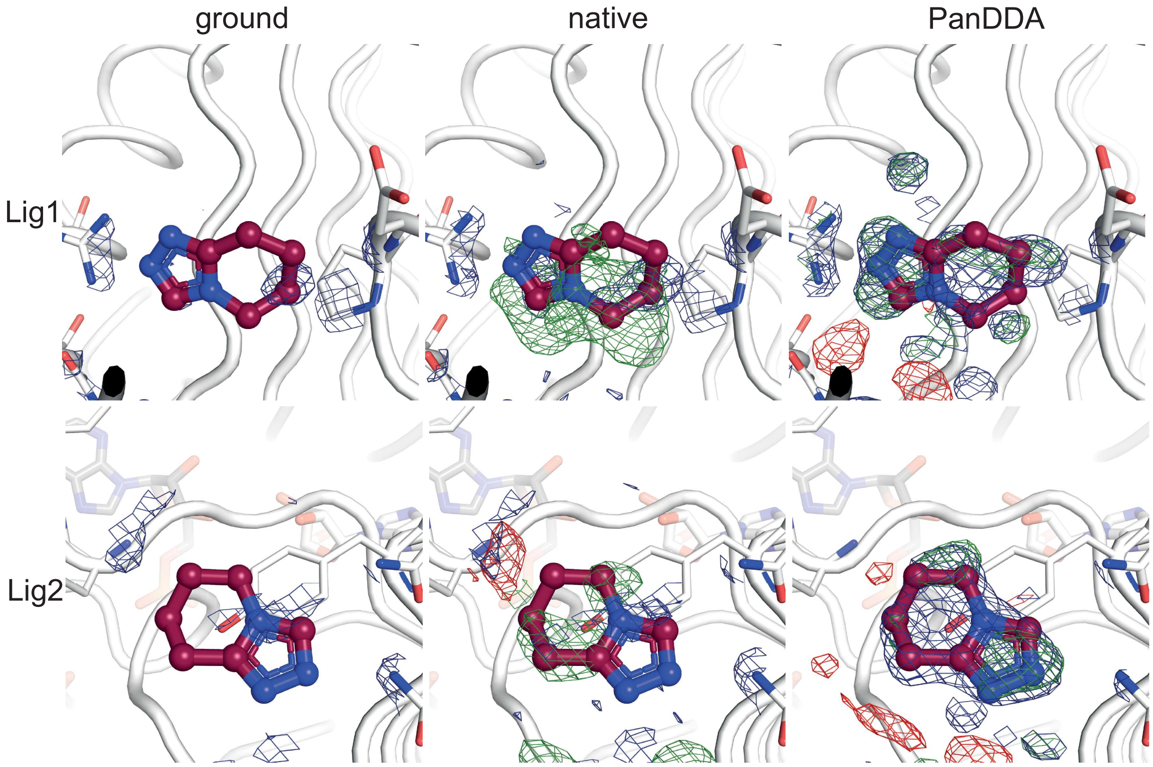

VT00219

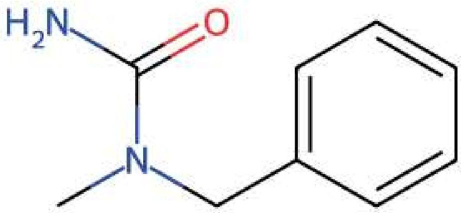

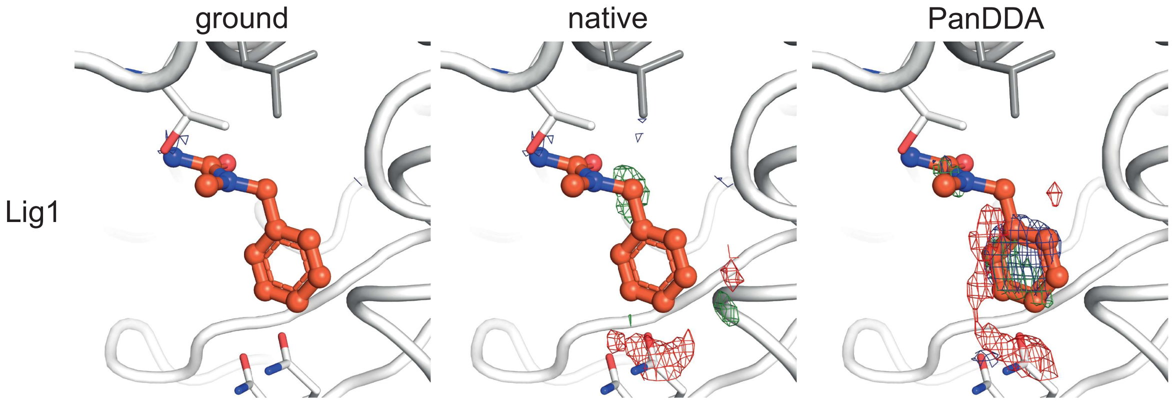

VT00221

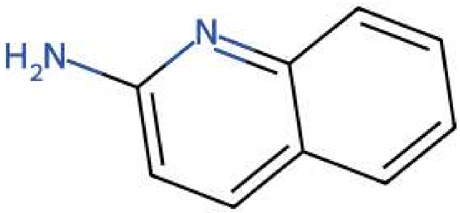

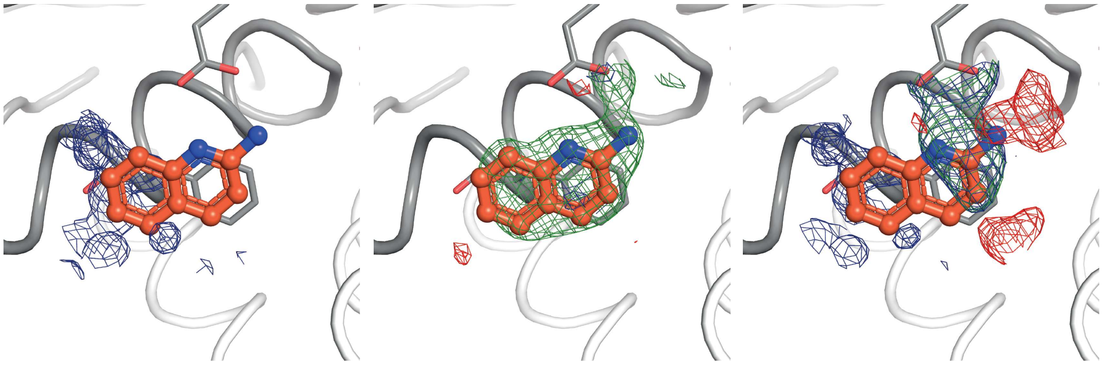

VT00222

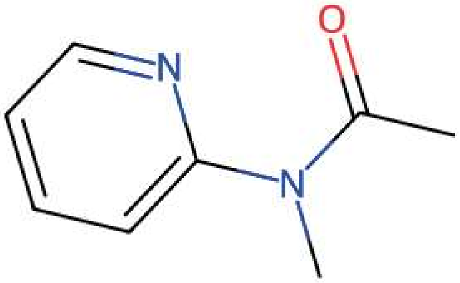

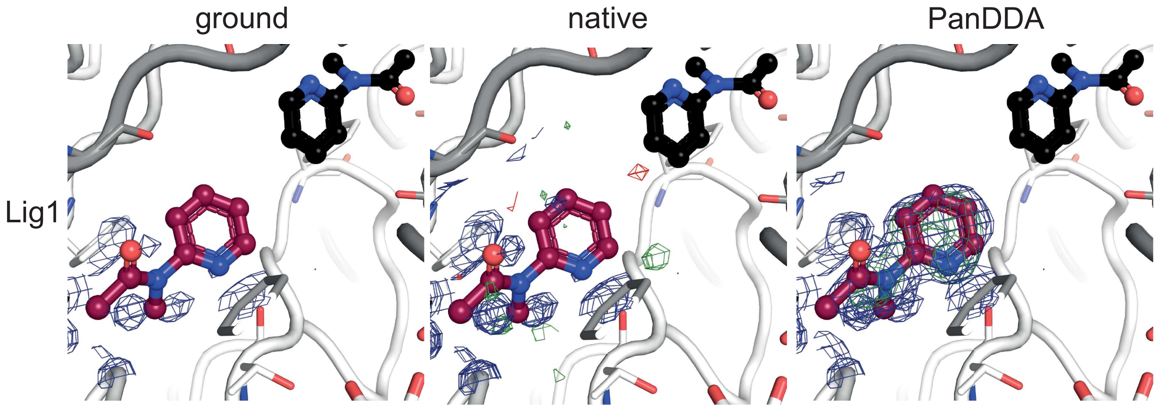

VT00223

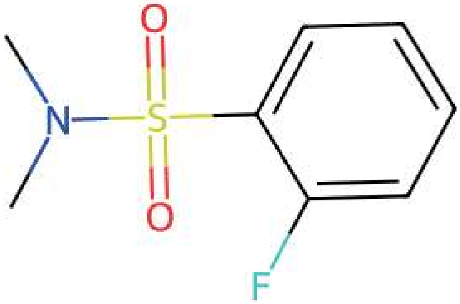

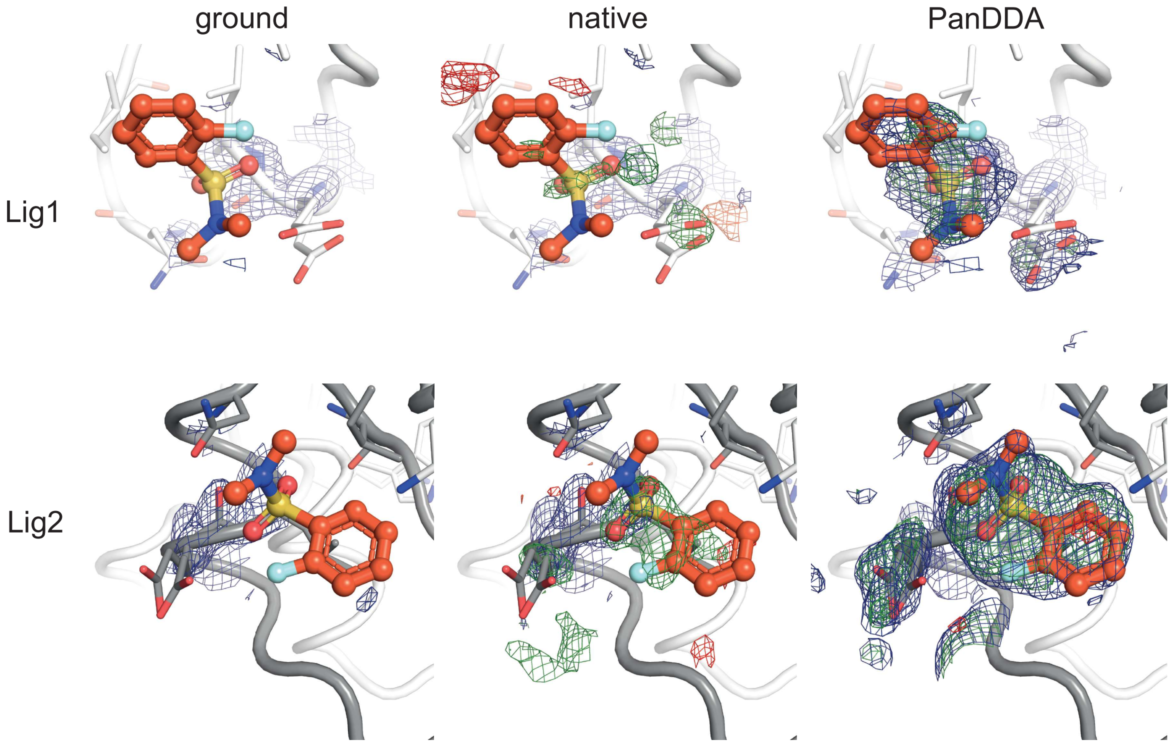

VT00224

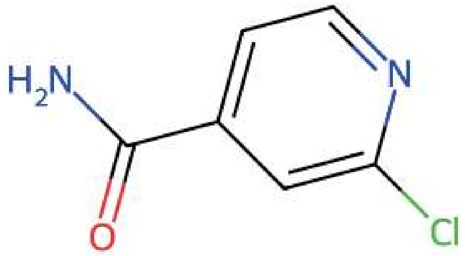

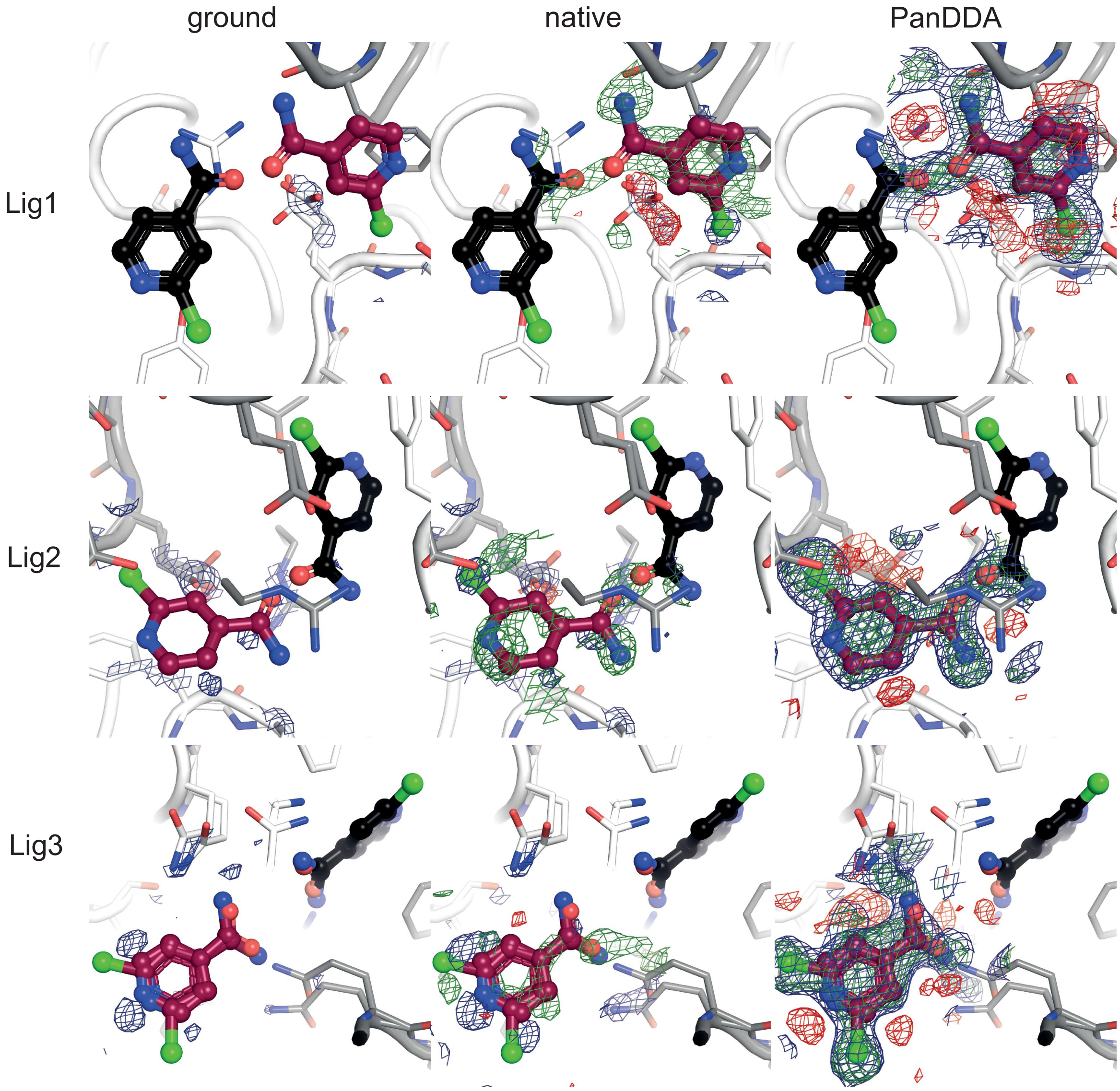

VT00228

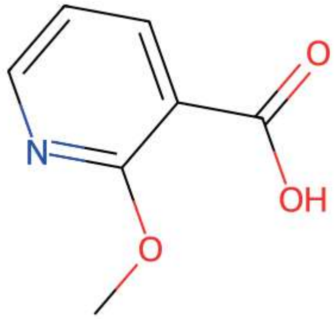

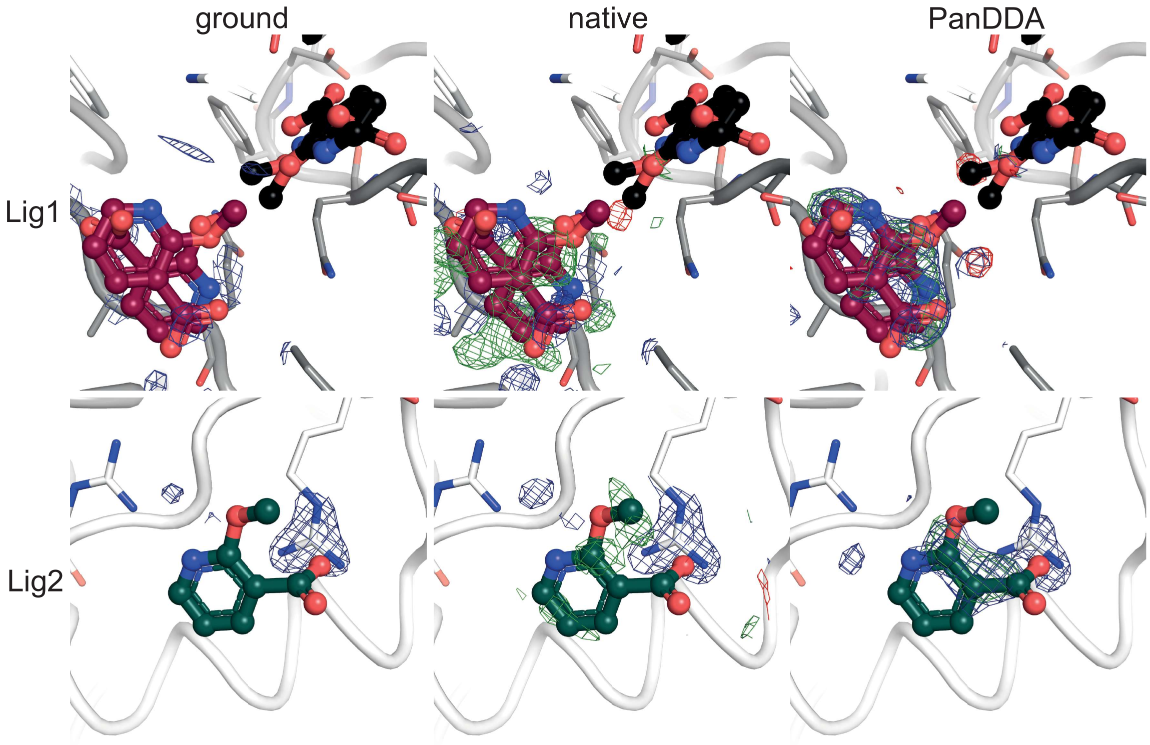

VT00229

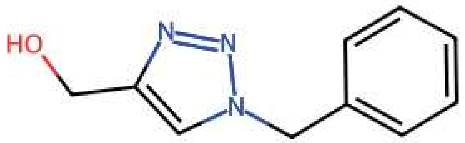

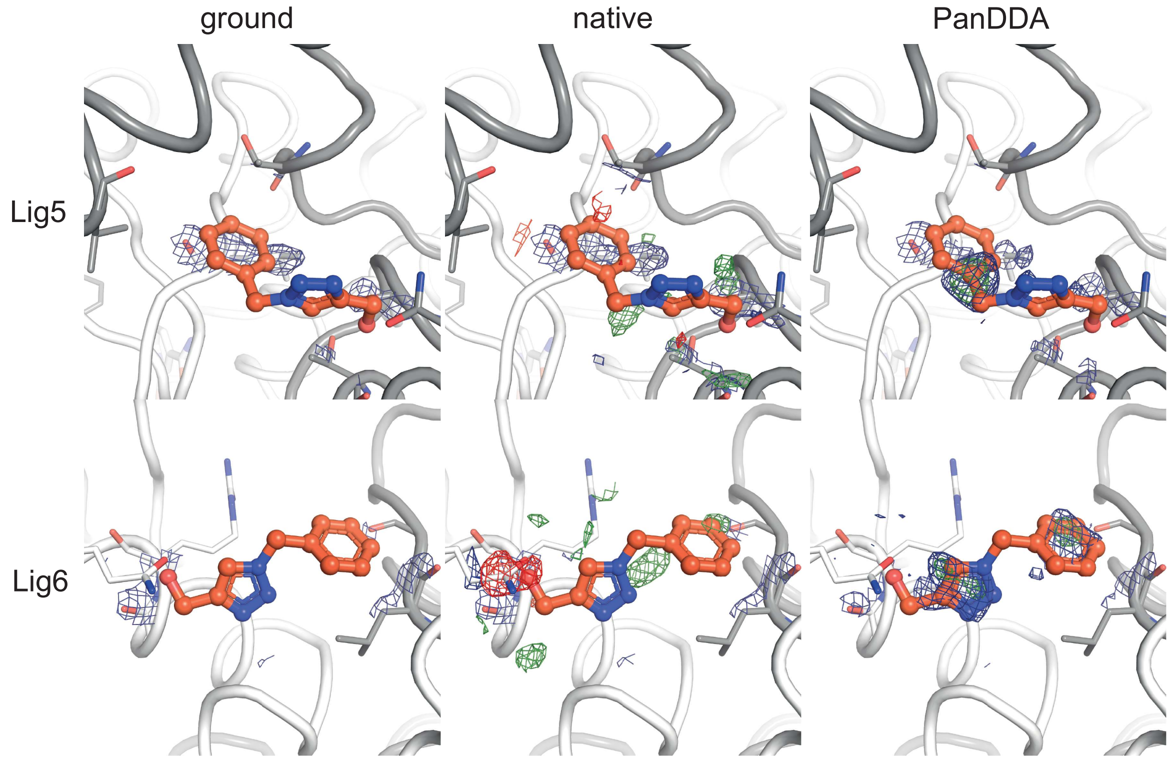

VT00229

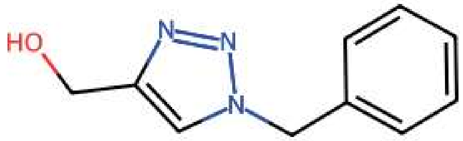

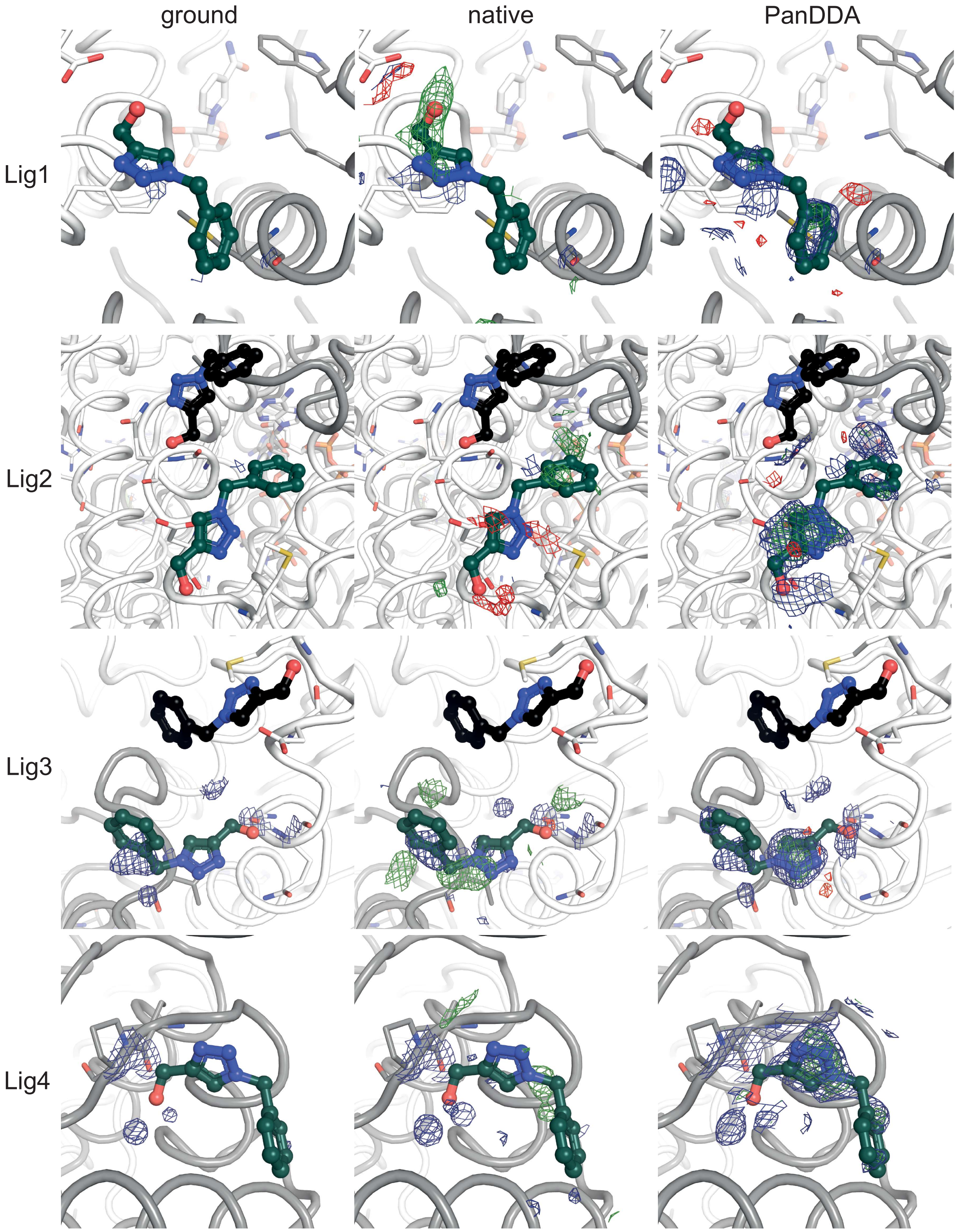

VT00230

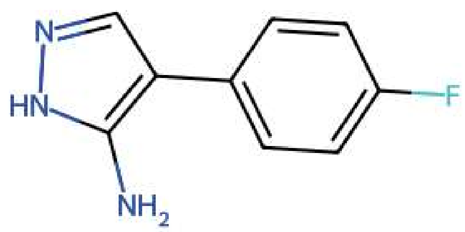

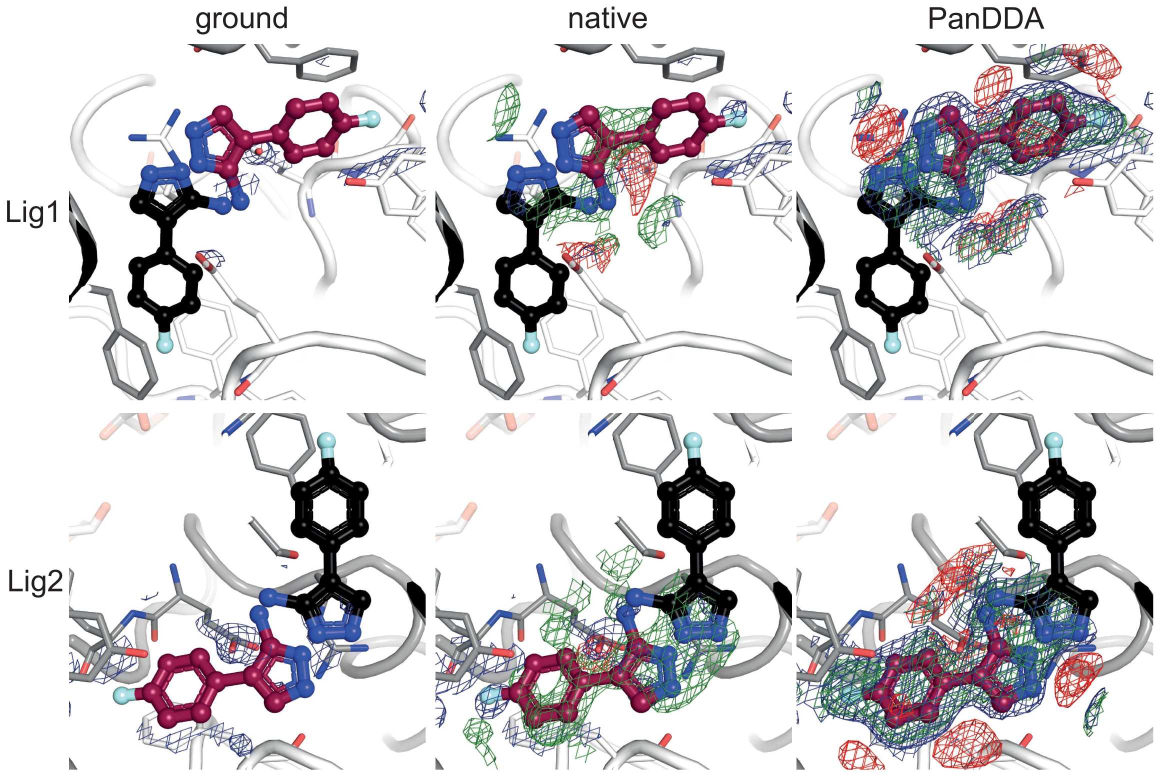

VT00234

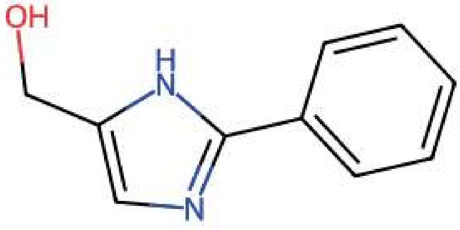

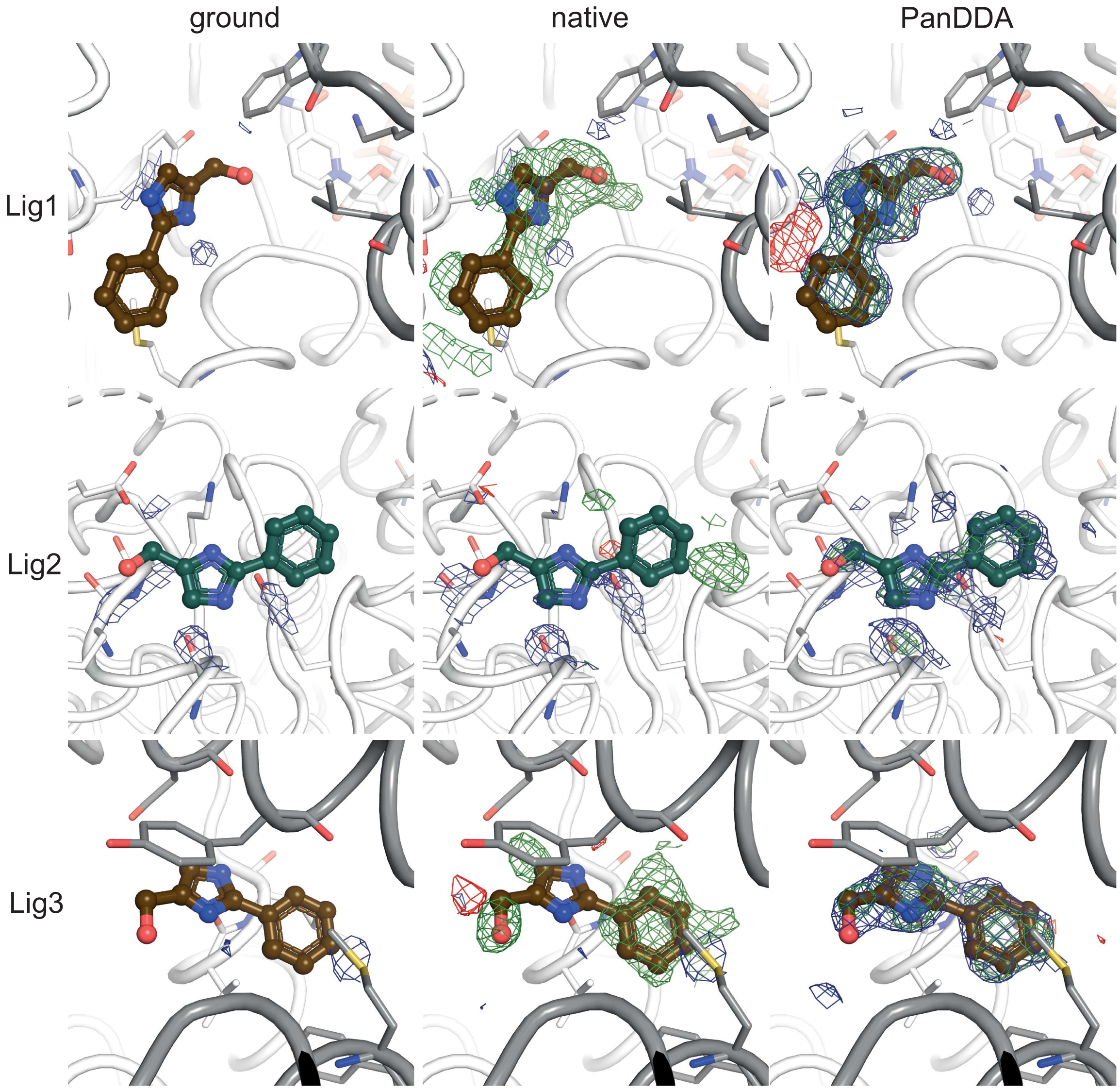

VT00236

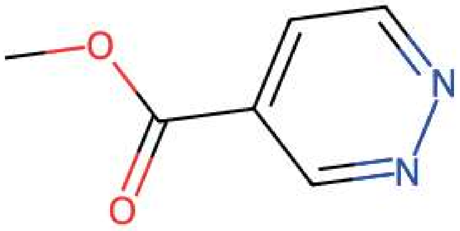

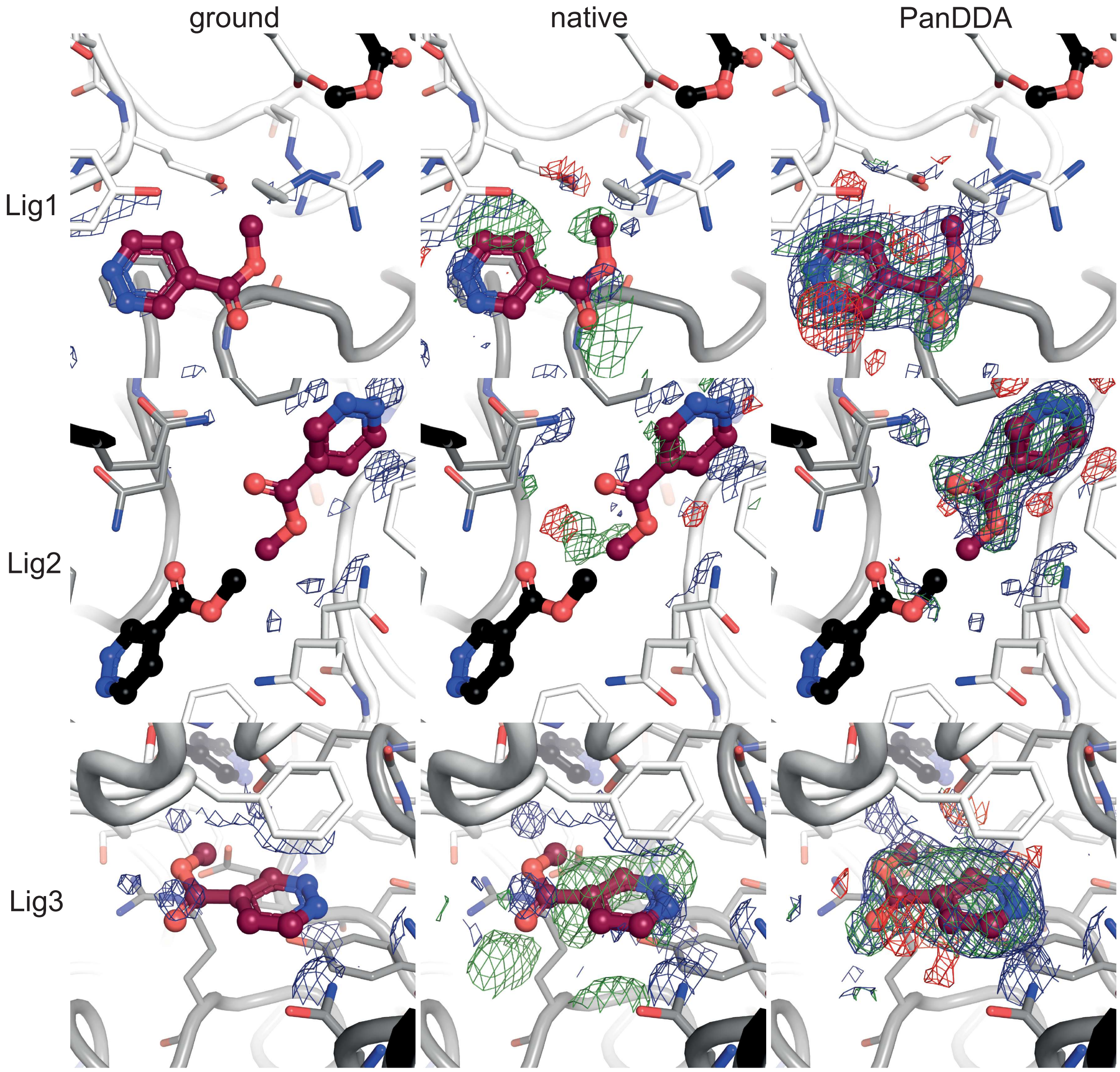

VT00253

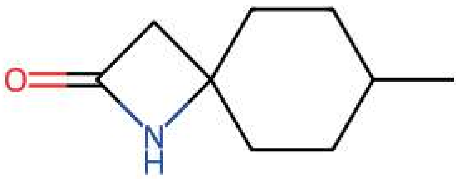

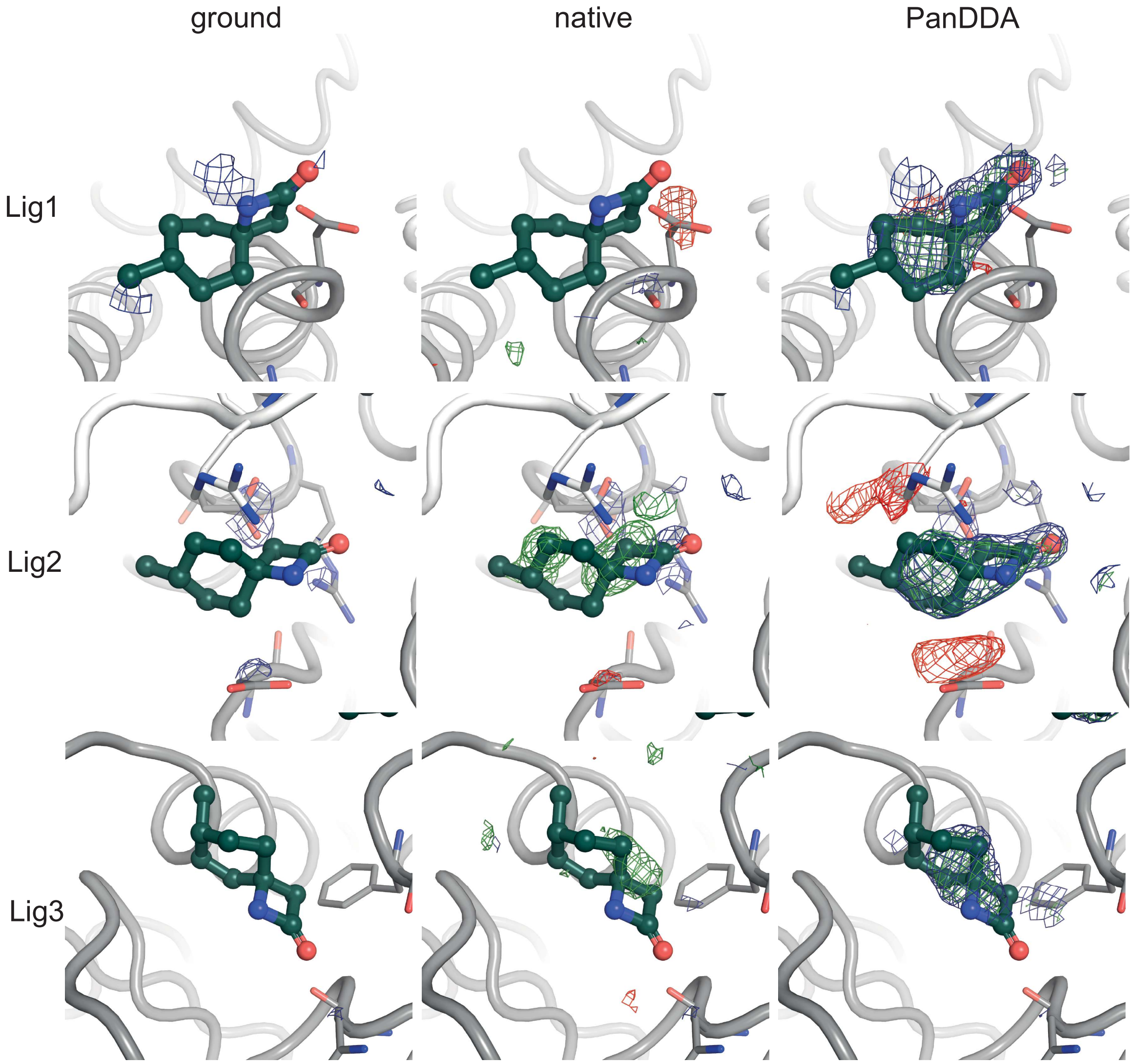

VT00254

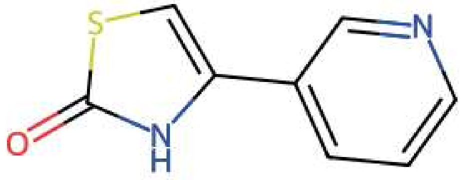

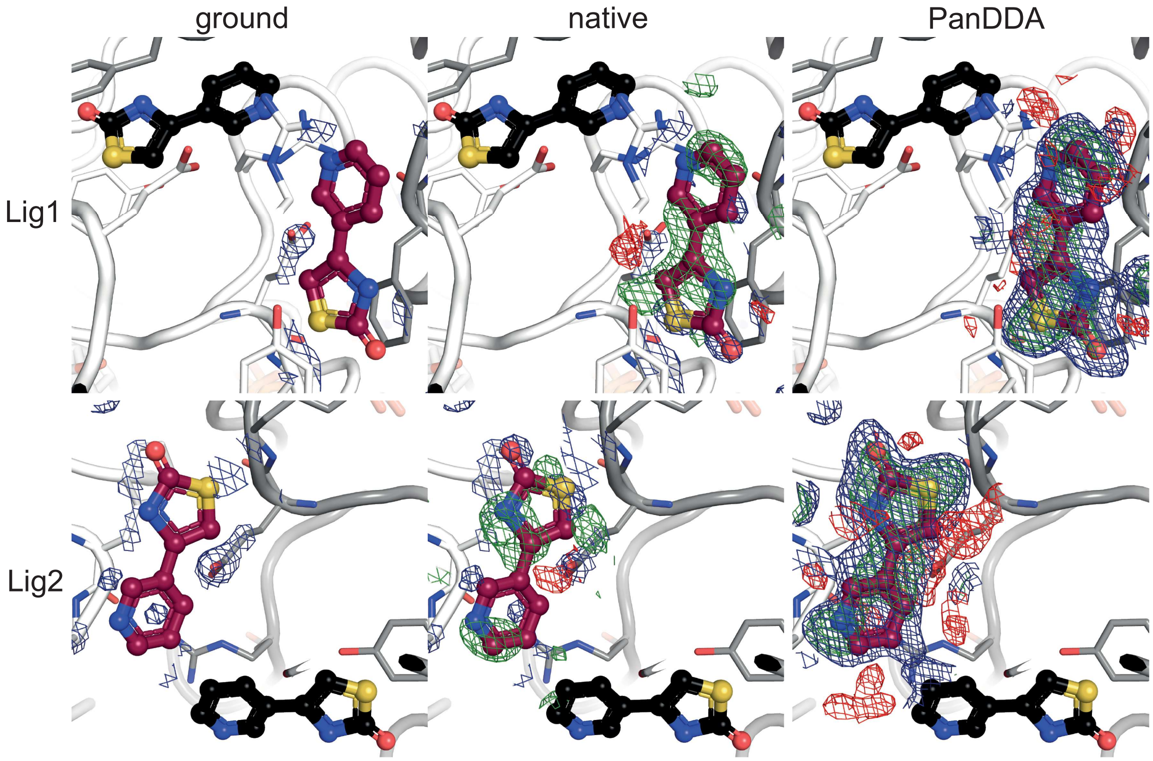

VT00257

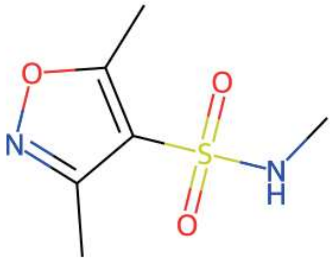

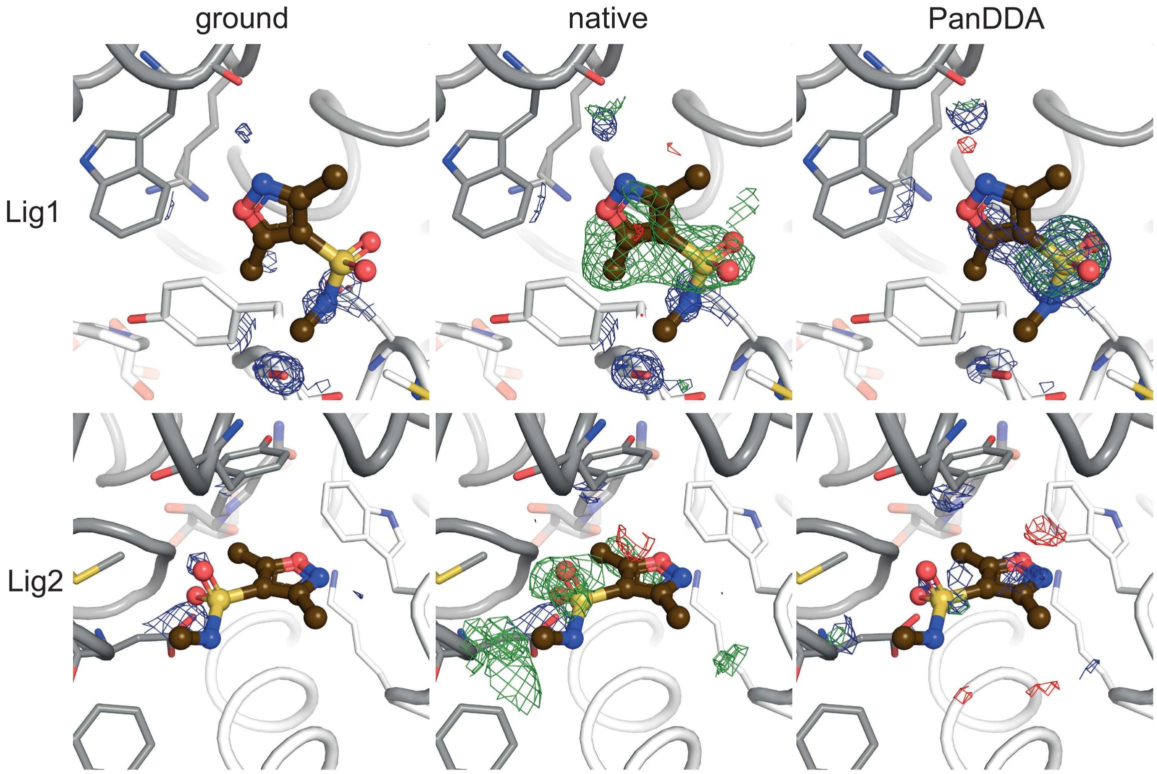

VT00259

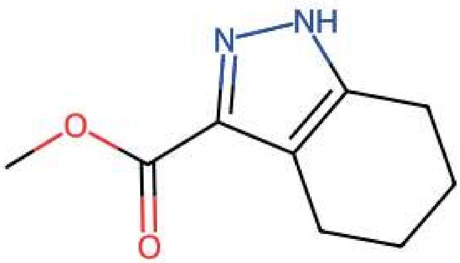

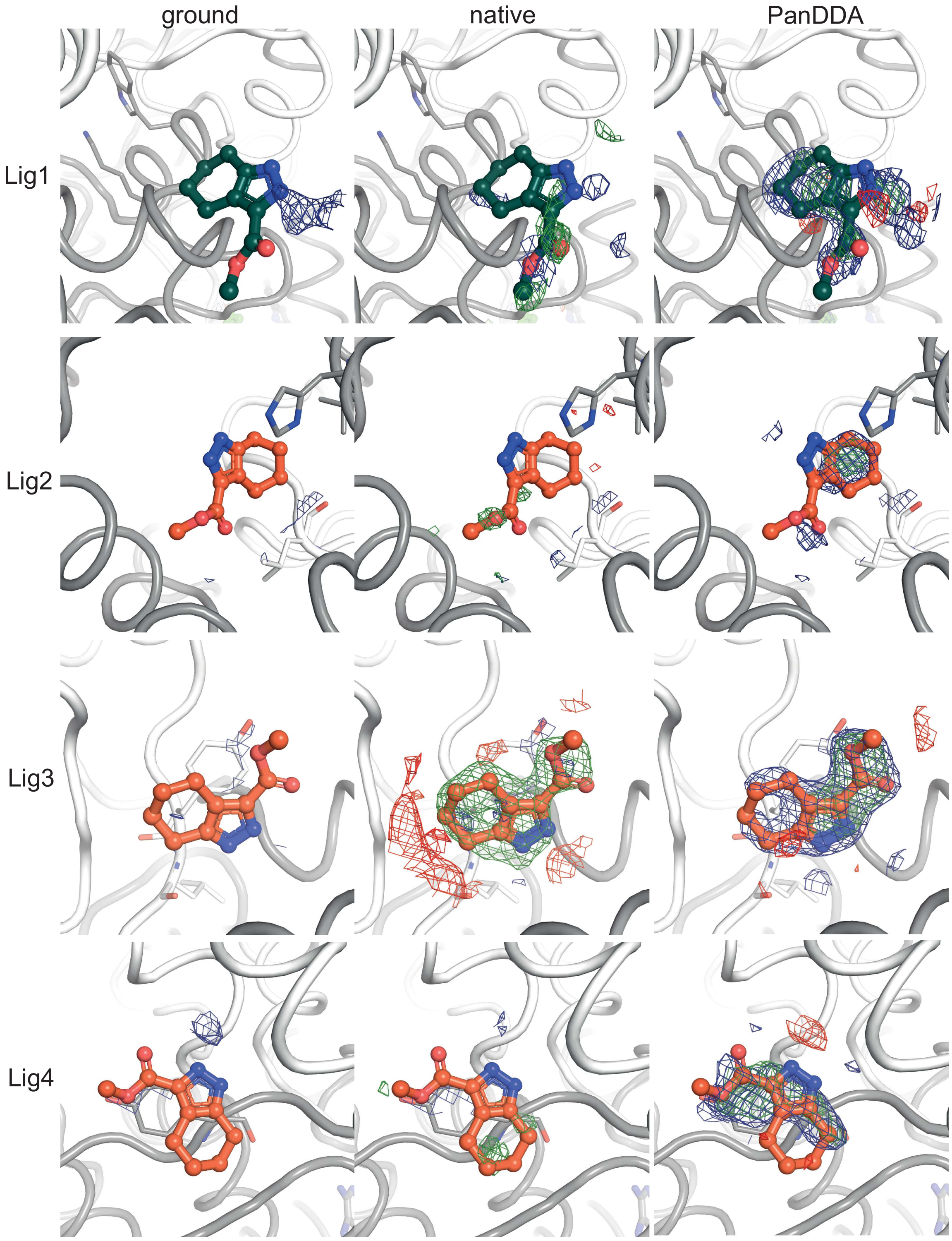

VT00261

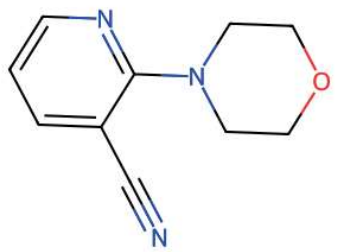

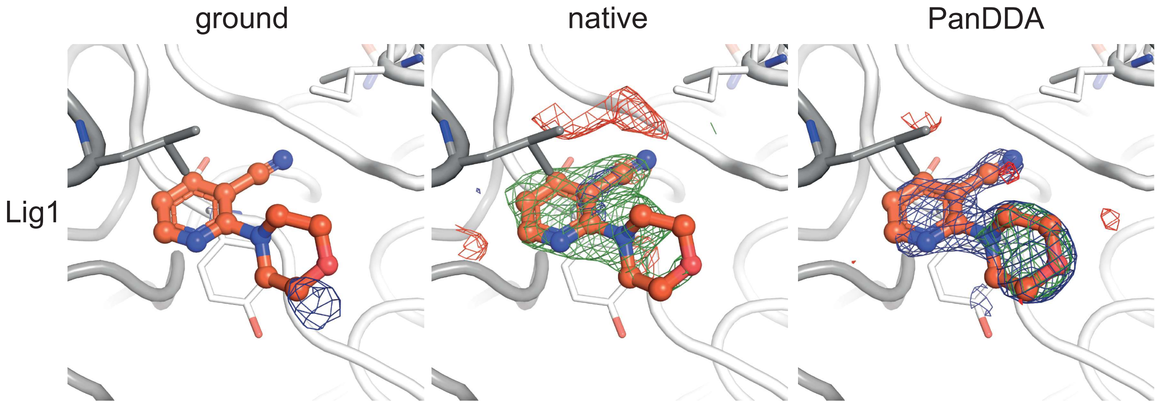

VT00268

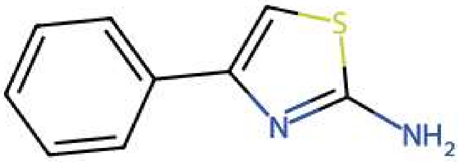

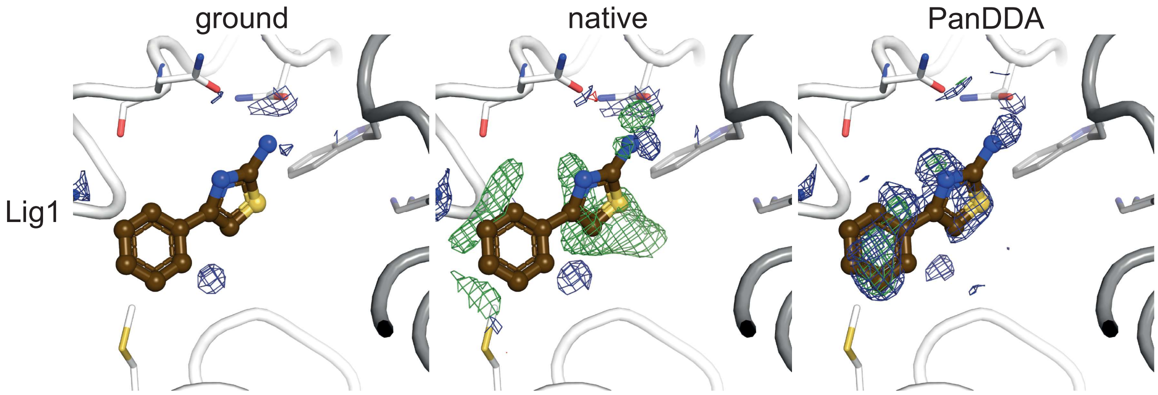

VT00403

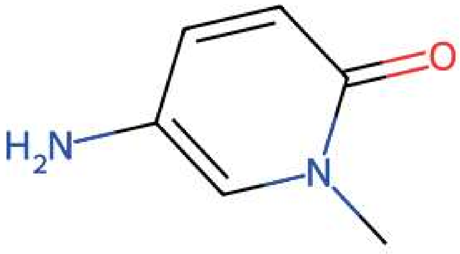

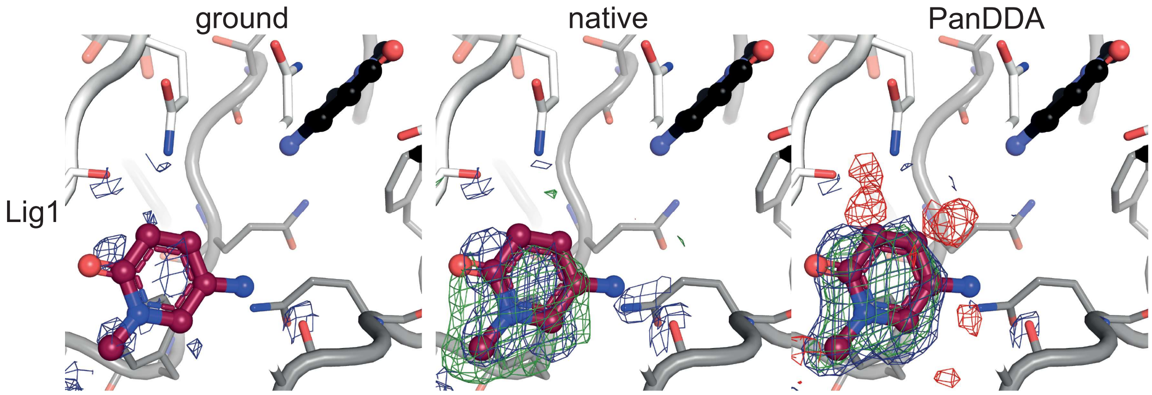

VT00405

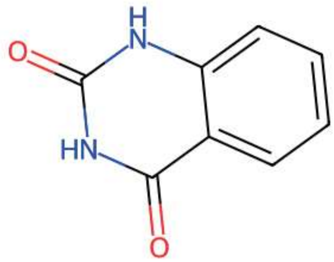

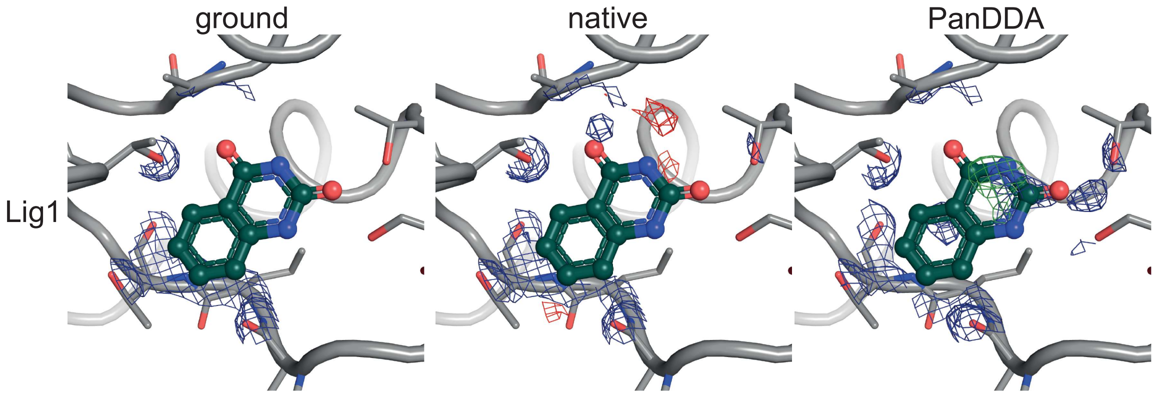

VT00407

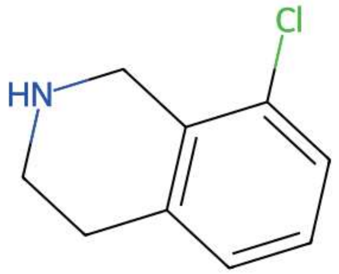

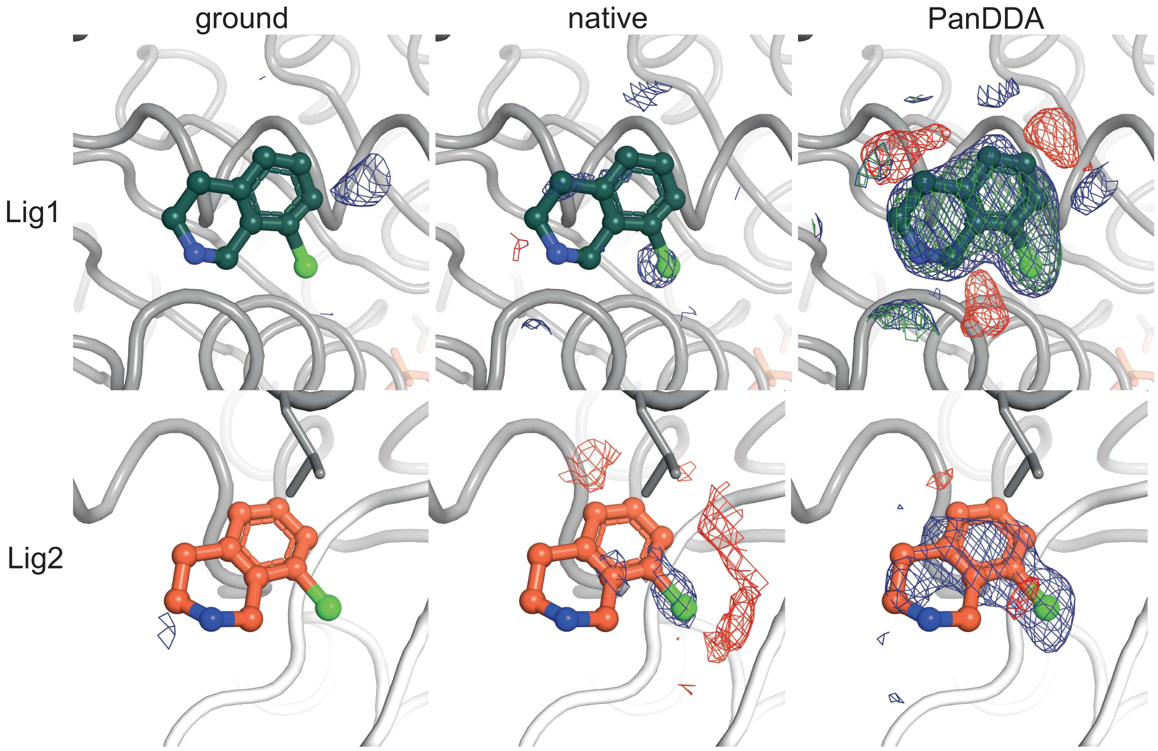

VT00409

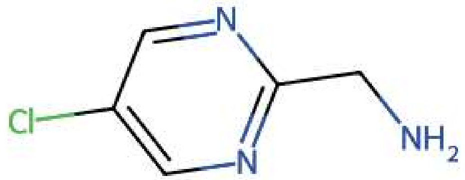

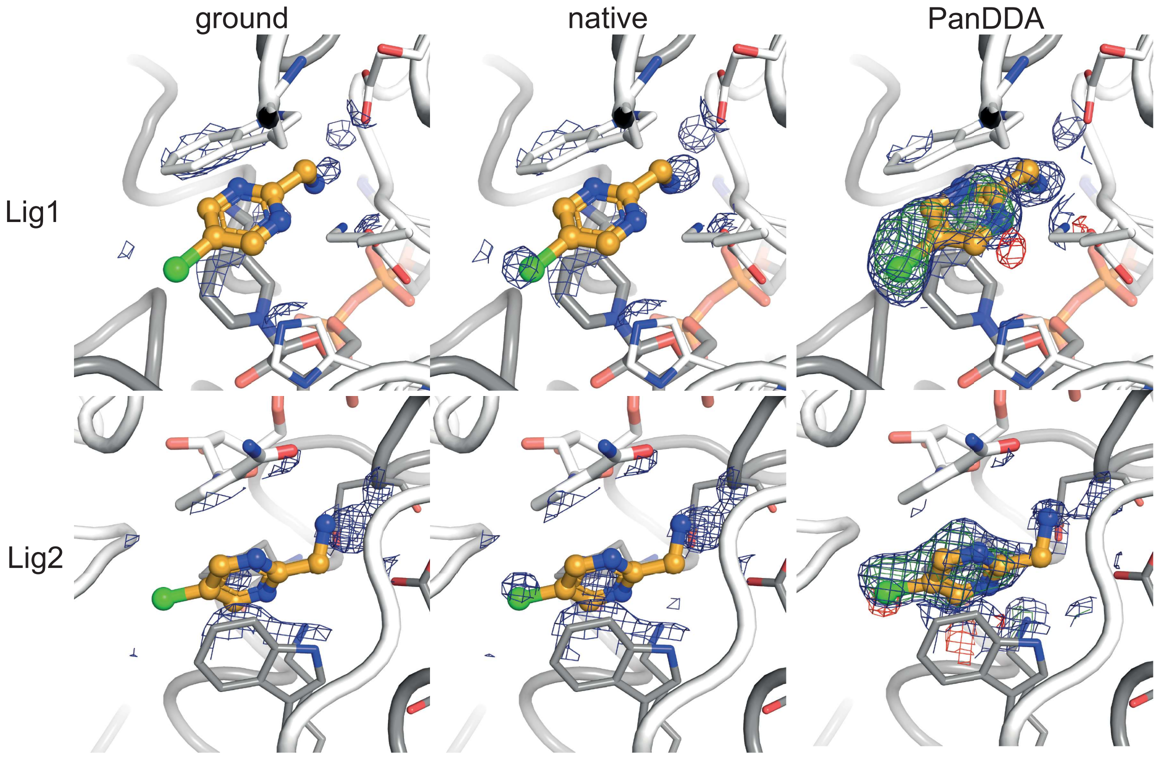

VT00416

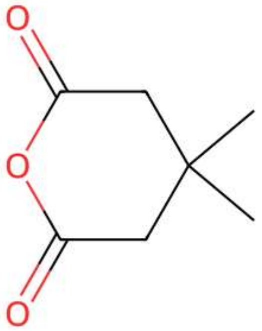

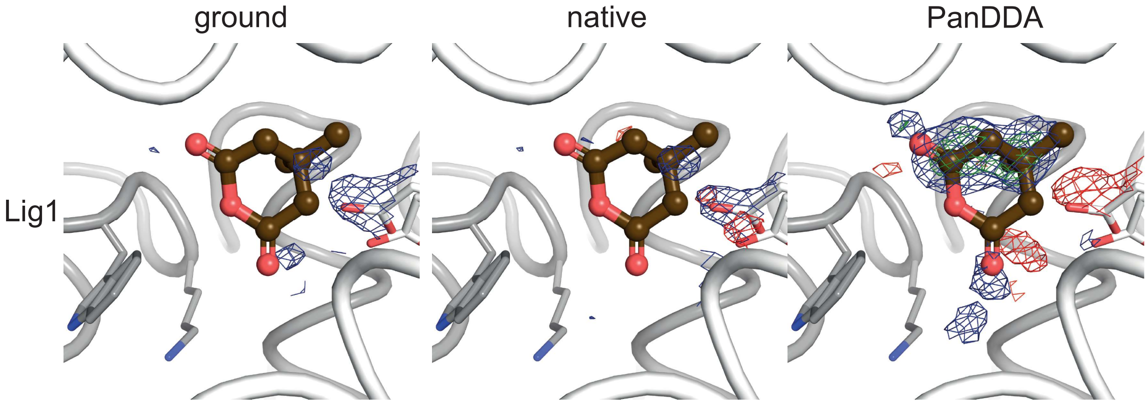

VT00417

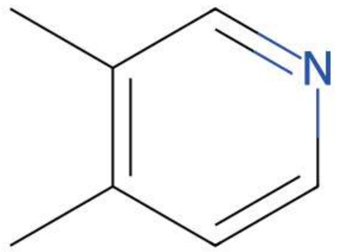

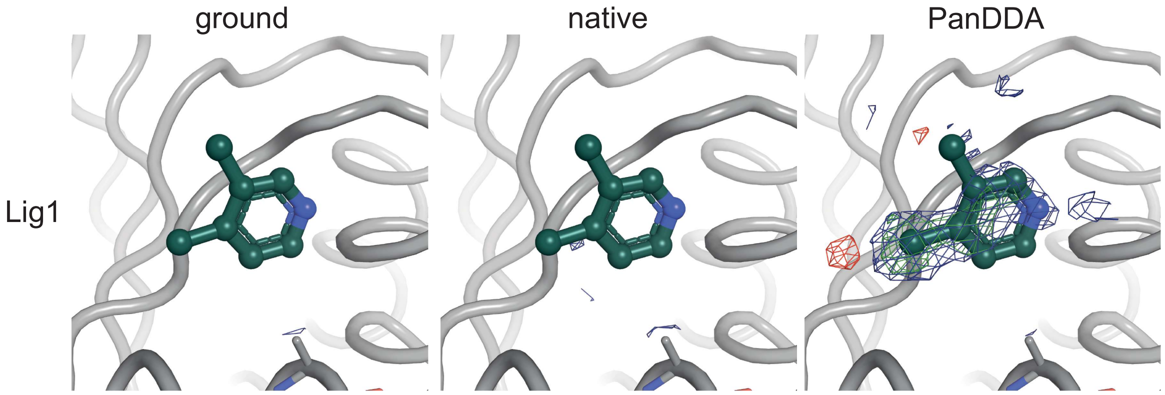

VT00423

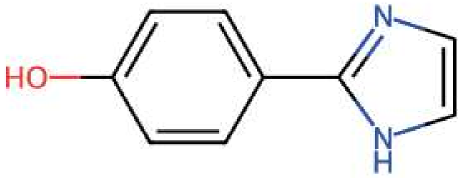

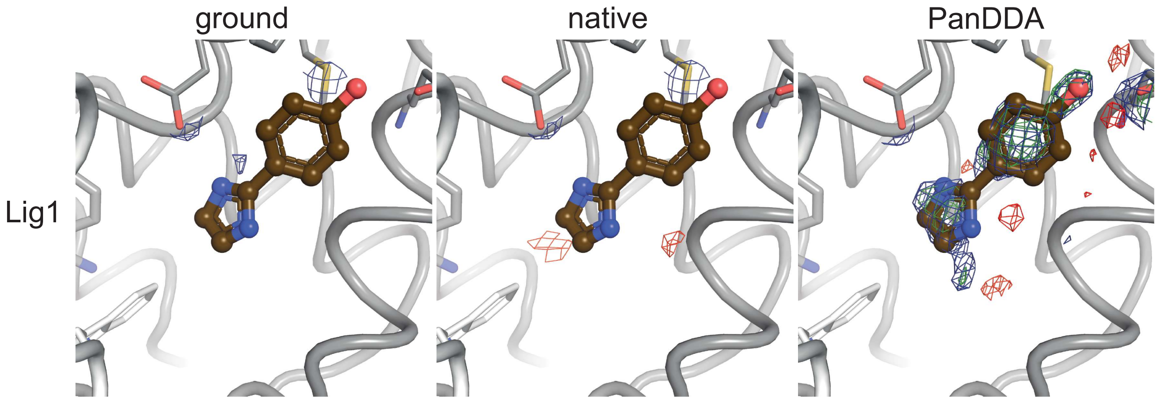

VT00424

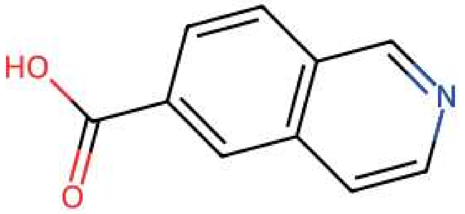

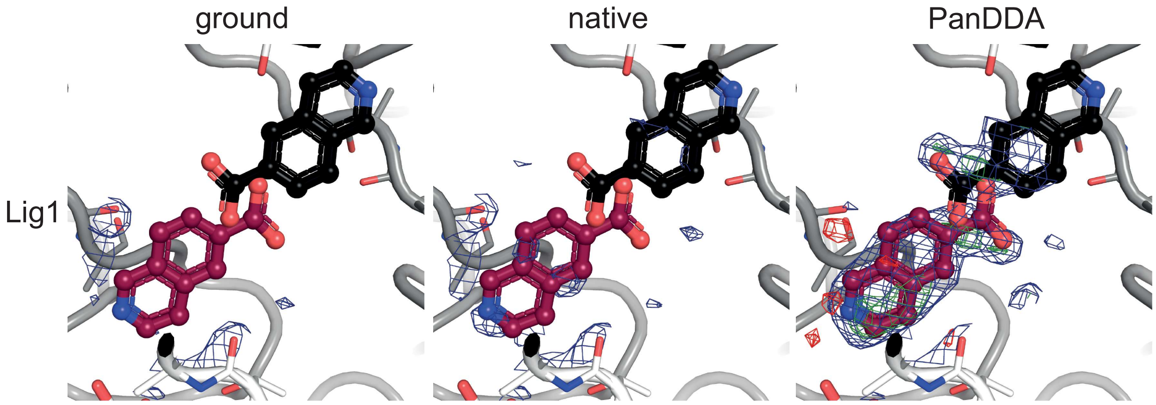

VT00427

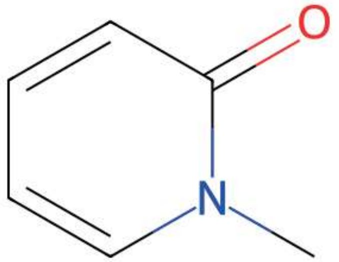

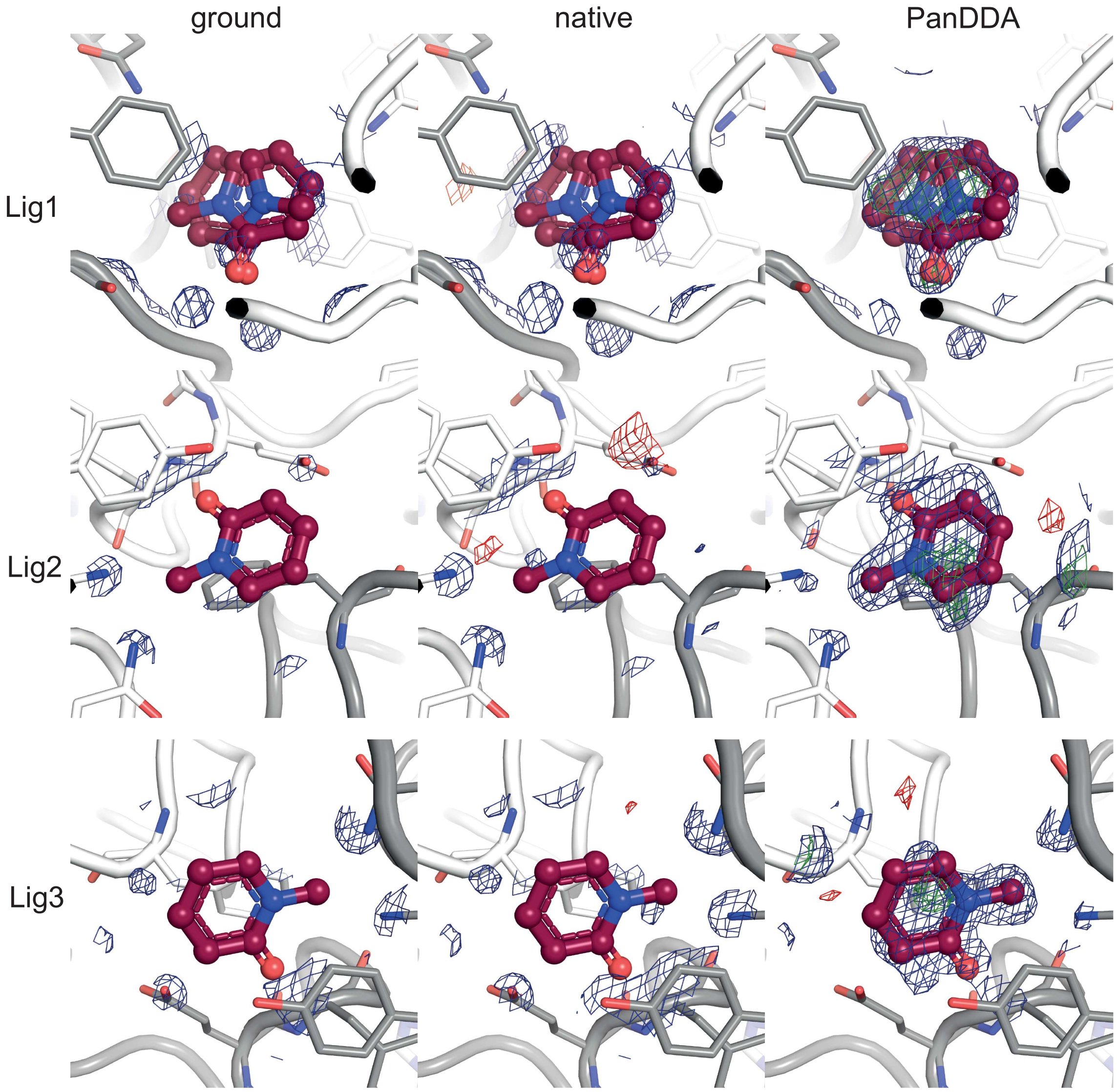

VT00428

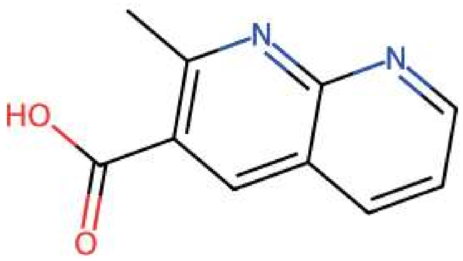

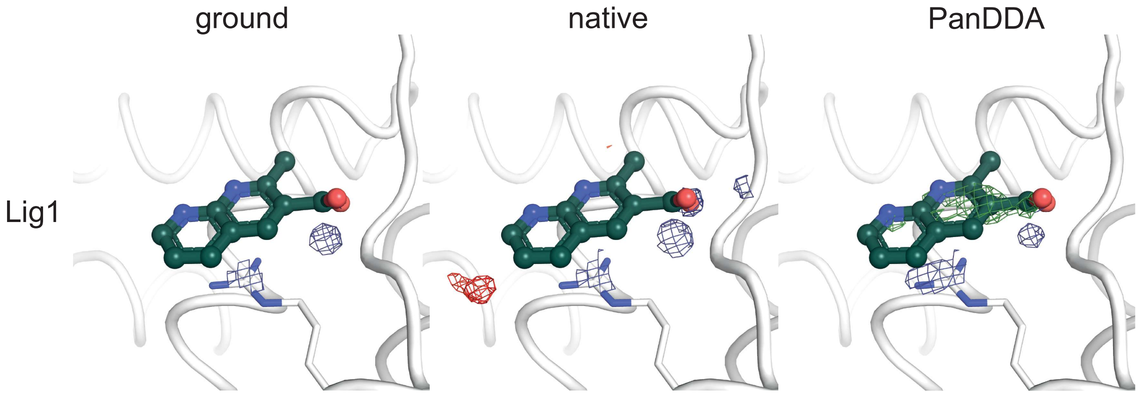

VT00438

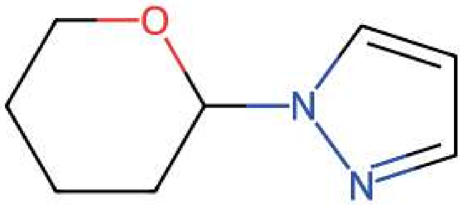

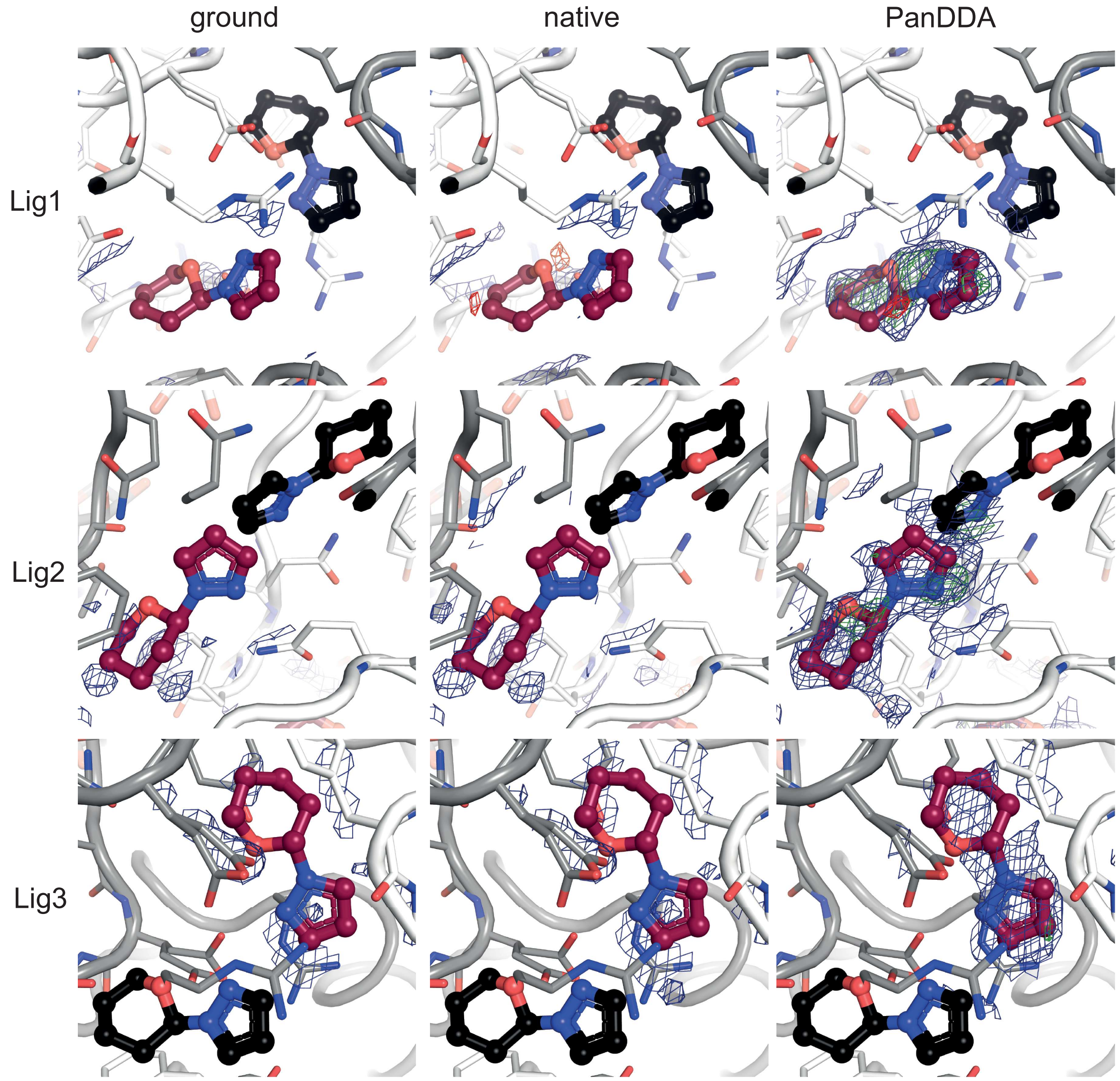

VT00440

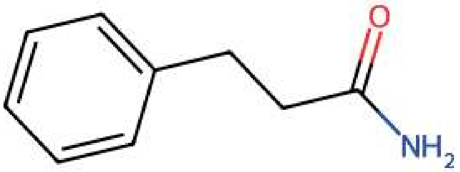

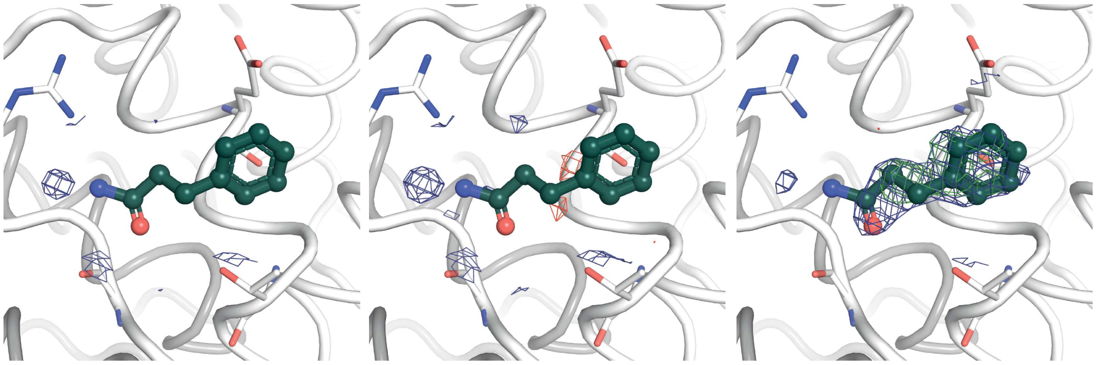

VT00441

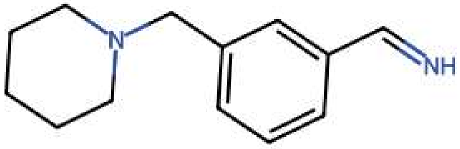

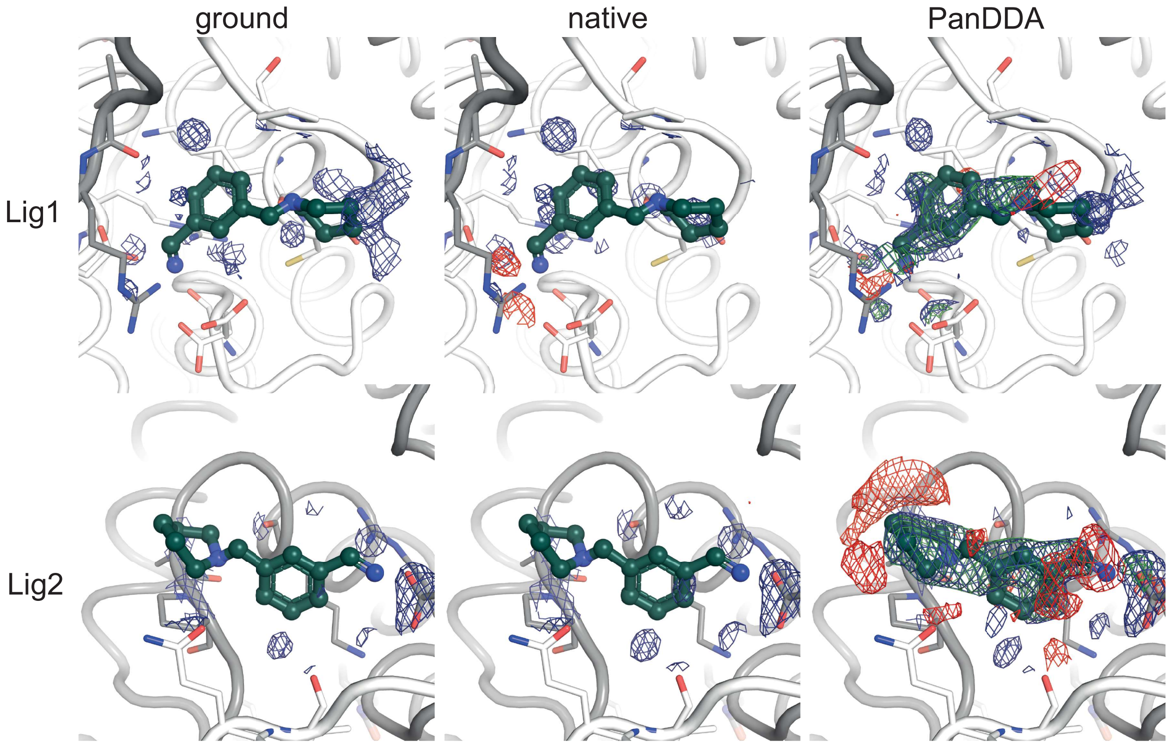

VT00442

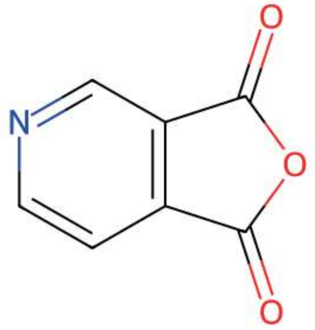

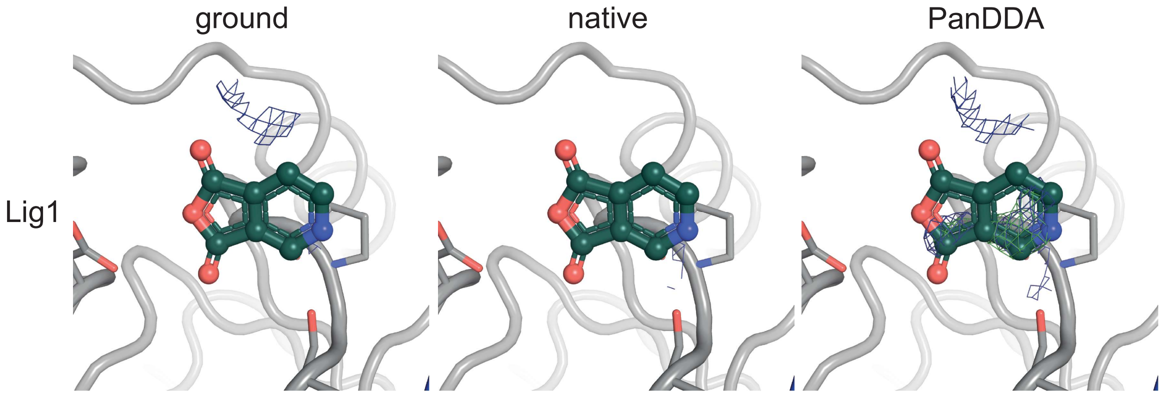

VT00445

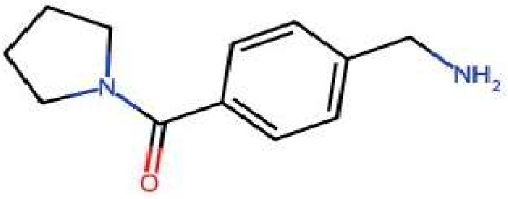

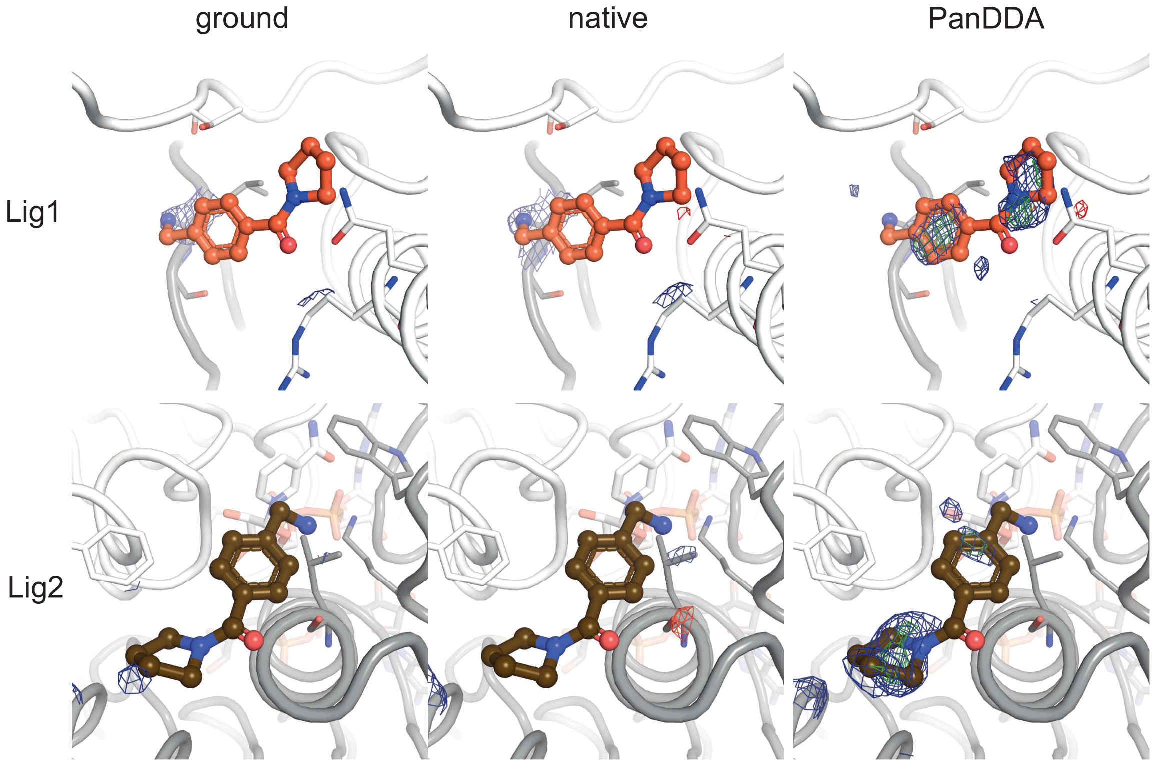

VT00446

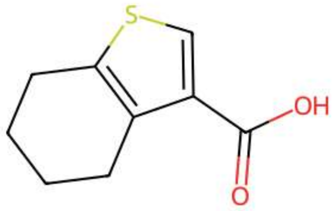

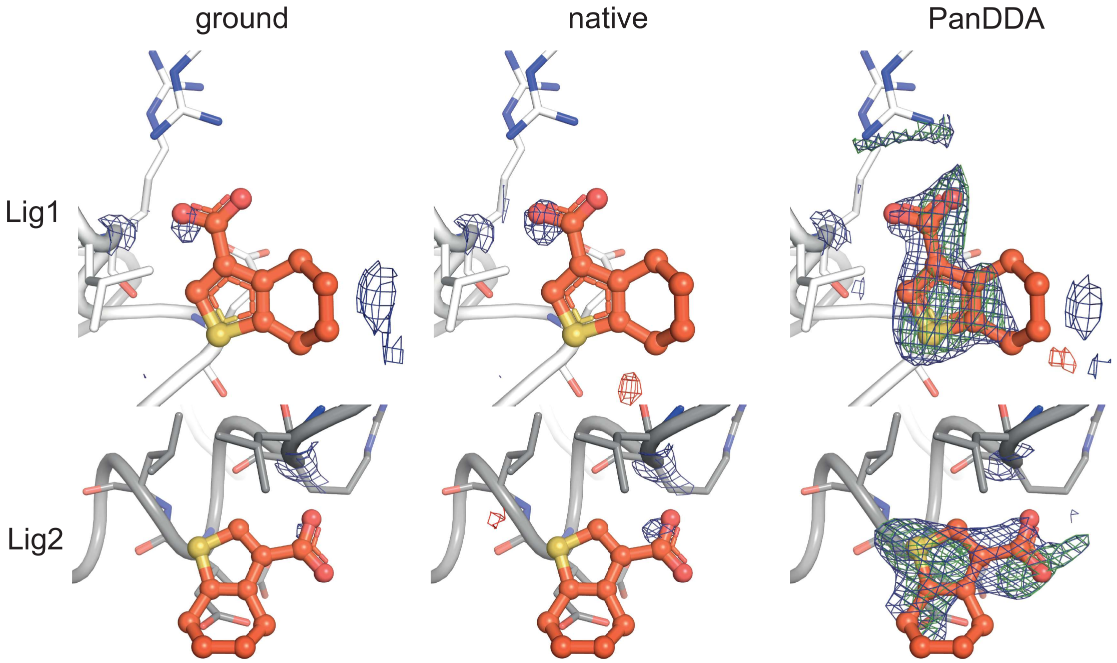

VT00447

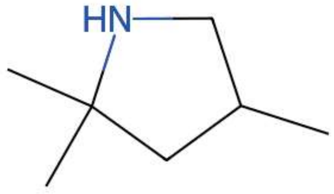

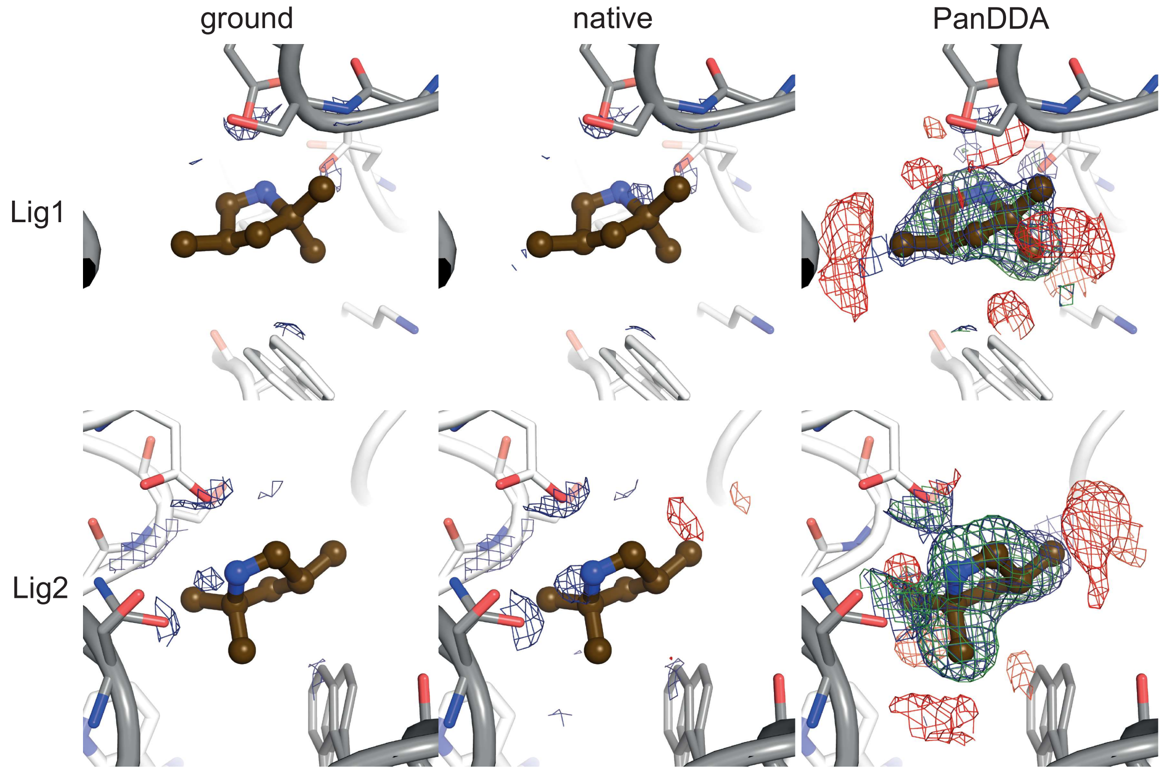

VT00451

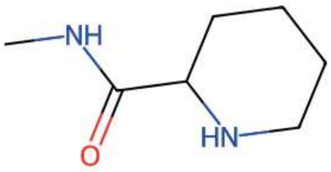

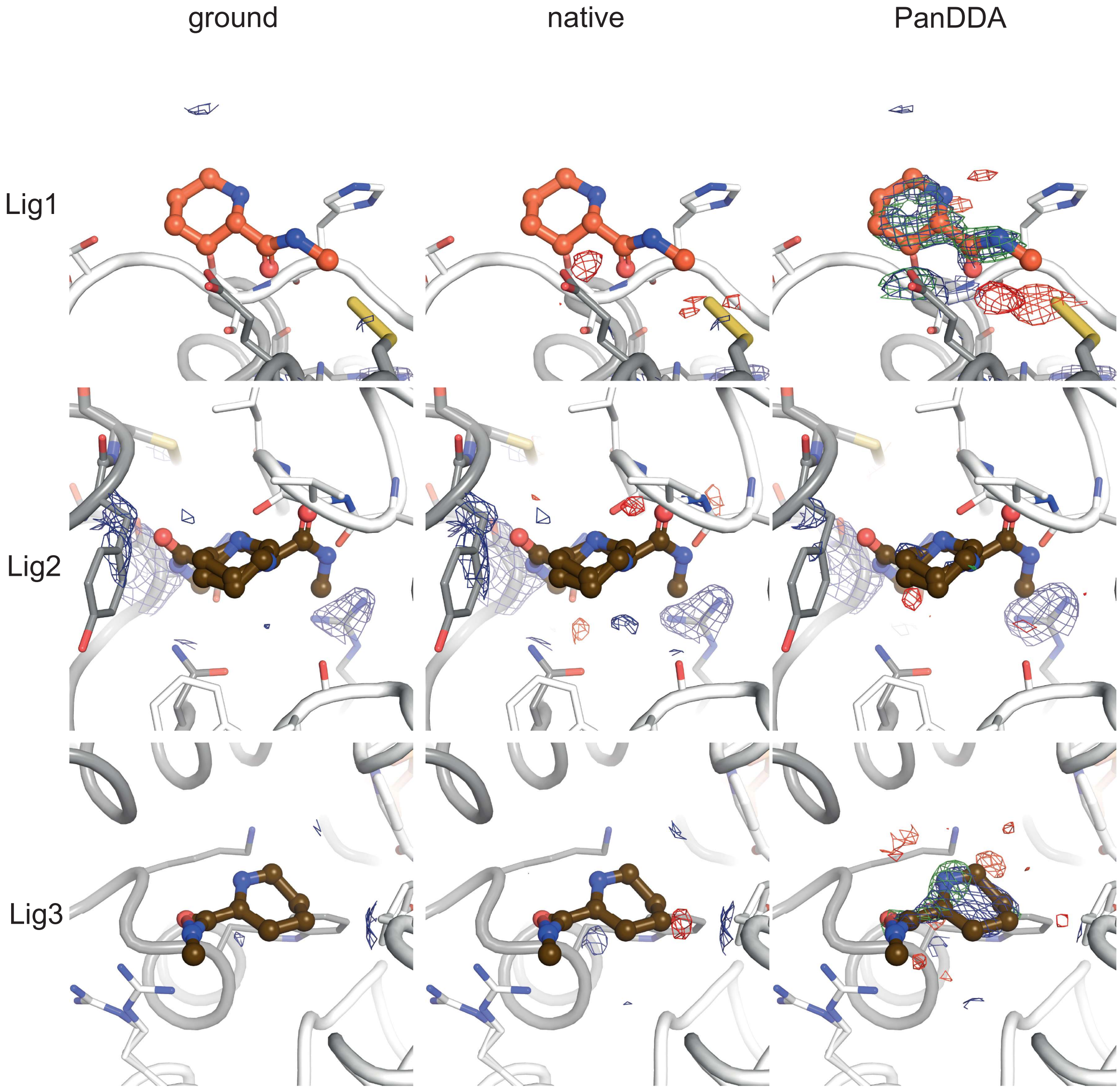

Supplement: Supplementary file 5 — Supplementary Data 2 [file 42004_2026_1897_MOESM5_ESM.pdf]
